# A post-transcriptional program of chemoresistance by AU-rich elements and TTP in quiescent leukemic cells

**DOI:** 10.1186/s13059-020-1936-4

**Published:** 2020-02-10

**Authors:** Sooncheol Lee, Douglas Micalizzi, Samuel S. Truesdell, Syed I. A. Bukhari, Myriam Boukhali, Jennifer Lombardi-Story, Yasutaka Kato, Min-Kyung Choo, Ipsita Dey-Guha, Fei Ji, Benjamin T. Nicholson, David T. Myers, Dongjun Lee, Maria A. Mazzola, Radhika Raheja, Adam Langenbucher, Nicholas J. Haradhvala, Michael S. Lawrence, Roopali Gandhi, Christopher Tiedje, Manuel D. Diaz-Muñoz, David A. Sweetser, Ruslan Sadreyev, David Sykes, Wilhelm Haas, Daniel A. Haber, Shyamala Maheswaran, Shobha Vasudevan

**Affiliations:** 1 Massachusetts General Hospital Cancer Center, Harvard Medical School, 185 Cambridge St, CPZN4202, Boston, MA 02114 USA; 2 Department of Medicine, Massachusetts General Hospital and Harvard Medical School, Boston, 02114 Massachusetts USA; 3 Center for Regenerative Medicine, Massachusetts General Hospital, Harvard Medical School, Boston, MA 02114 USA; 4 Harvard Stem Cell Institute, Harvard University, Cambridge, MA 02138 USA; 5 Laboratory of Oncology, Hokuto Hospital, Obihiro, Japan; 6 Cutaneous Biology Research Center, Massachusetts General Hospital and Harvard Medical School, Charlestown, MA 02129 USA; 7 Department of Molecular Biology, Massachusetts General Hospital, Harvard Medical School, Boston, MA 02114 USA; 8 Department of Convergence Medical Science, Pusan National University School of Medicine, Yangsan, 50612 1257-1258, South Korea; 9 Center for Neurological Diseases, Brigham & Women’s Hospital, Harvard Medical School, Boston, MA 02115 USA; 10 Department of Pathology, Massachusetts General Hospital, Harvard Medical School, Charlestown, MA 02129 USA; 11 Broad Institute of Harvard & MIT, Cambridge, MA 02142 USA; 12 Department of Cellular and Molecular Medicine, Center for Healthy Aging, University of Copenhagen, Blegdamsvej 3B, 2200 Copenhagen, Denmark; 13 Centre de Physiopathologie Toulouse-Purpan, INSERM UMR1043/CNRS U5282, Toulouse, France; 14 Department of Pediatrics, Divisions of Pediatric Hematology/Oncology and Medical Genetics, Massachusetts General Hospital, Harvard Medical School, Boston, MA 02114 USA; 15 Howard Hughes Medical Institute, Chevy Chase, MD 20815 USA; 16 Department of Surgery, Massachusetts General Hospital and Harvard Medical School, Charlestown, MA 02129 USA

**Keywords:** Quiescence, Chemoresistance, Post-transcriptional regulation, AU-rich elements, TTP

## Abstract

**Background:**

Quiescence (G0) is a transient, cell cycle-arrested state. By entering G0, cancer cells survive unfavorable conditions such as chemotherapy and cause relapse. While G0 cells have been studied at the transcriptome level, how post-transcriptional regulation contributes to their chemoresistance remains unknown.

**Results:**

We induce chemoresistant and G0 leukemic cells by serum starvation or chemotherapy treatment. To study post-transcriptional regulation in G0 leukemic cells, we systematically analyzed their transcriptome, translatome, and proteome. We find that our resistant G0 cells recapitulate gene expression profiles of in vivo chemoresistant leukemic and G0 models. In G0 cells, canonical translation initiation is inhibited; yet we find that inflammatory genes are highly translated, indicating alternative post-transcriptional regulation. Importantly, AU-rich elements (AREs) are significantly enriched in the upregulated G0 translatome and transcriptome. Mechanistically, we find the stress-responsive p38 MAPK-MK2 signaling pathway stabilizes ARE mRNAs by phosphorylation and inactivation of mRNA decay factor, Tristetraprolin (TTP) in G0. This permits expression of ARE mRNAs that promote chemoresistance. Conversely, inhibition of TTP phosphorylation by p38 MAPK inhibitors and non-phosphorylatable TTP mutant decreases ARE-bearing TNFα and DUSP1 mRNAs and sensitizes leukemic cells to chemotherapy. Furthermore, co-inhibiting p38 MAPK and TNFα prior to or along with chemotherapy substantially reduces chemoresistance in primary leukemic cells ex vivo and in vivo.

**Conclusions:**

These studies uncover post-transcriptional regulation underlying chemoresistance in leukemia. Our data reveal the p38 MAPK-MK2-TTP axis as a key regulator of expression of ARE-bearing mRNAs that promote chemoresistance. By disrupting this pathway, we develop an effective combination therapy against chemosurvival.

**Electronic supplementary material:**

**Supplementary information** accompanies this paper at 10.1186/s13059-020-1936-4.

## Background

Quiescent (G0) cells are an assortment of reversibly arrested cells, including dormant stem cells, which are found as a clinically relevant subpopulation in cancers [1–4]. Such cells are anti-proliferative, anti-differentiation, and anti-apoptotic and show distinct properties including resistance to harsh conditions [1, 2, 5–10]. G0 cells show specific gene expression that may underlie their resistance and other properties [1, 2, 8–10]. Analyses from multiple groups revealed some genes upregulated at the transcriptional level [1, 8, 11]. Altered polyadenylation site selection on mRNAs produces longer 3′-untranslated regions (3′UTRs) in G0 compared to proliferating cells—which increases 3′UTR elements that can mediate post-transcriptional gene expression regulation [12]. Our previous data demonstrated that translation mechanisms are distinct in G0 leukemic cells, with decreased canonical translation mechanisms and increase in alternative mechanisms that involve non-canonical translation initiation factors [13] and 3′UTR-mediated specific mRNA translation [14]. These data suggest that alternate post-transcriptional mechanisms in G0 cancer cells may regulate a distinct translatome to mediate their resistance. Translated genes in G0, the post-transcriptional mechanisms involved, and outcomes on cancer persistence remain to be investigated.

We analyzed the translatome and proteome of chemotherapy-surviving G0 cancer cells, focusing on acute monocytic leukemia (AML), to provide comprehensive information that complement and expand previous transcriptome analyses [1, 2, 8, 11, 15, 16], by uncovering critical genes that are post-transcriptionally regulated for chemosurvival. G0 can be induced by growth factor deprivation or serum starvation and other conditions that isolate dormant cancer stem cells in distinct cell types [1, 6, 7]. Our data demonstrate that serum starvation-induced G0 AML cells are chemoresistant—similar to surviving AML cells, isolated after chemotherapy. Chemoresistant cells isolated via serum starvation, or as surviving cells post-chemotherapy, show inhibition of canonical translation mechanisms, indicating that non-canonical mechanisms express specific mRNAs when these cells are chemoresistant. Consistently, the translatomes and proteomes of serum-starved G0 and chemosurviving cells show greater similarity than the transcriptomes alone. Our data reveal that DNA damage and stress signaling cause post-transcriptional alterations to produce a specialized gene expression program of pro-inflammatory, immune effectors that elicit chemosurvival.

## Results

### Serum starvation or AraC treatment induces a quiescent and chemoresistant state of leukemic cells

**Fig. 1:**
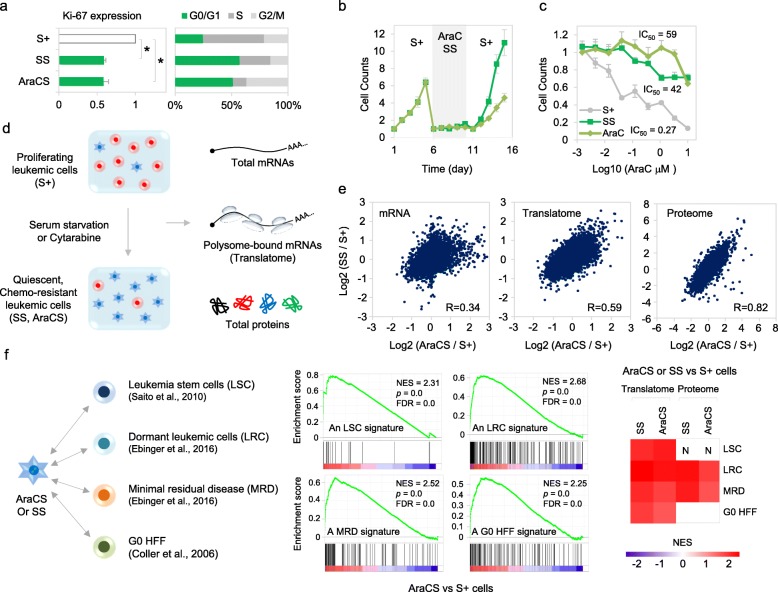
G0 leukemic cells induced by AraC or serum starvation are chemoresistant and recapitulate gene expression programs of in vivo chemoresistant and G0 models. **a** Ki67 translatome level and flow cytometric quantification of G0/G1, S, and G2/M phases, using BrdU and PI staining. Proliferating THP1 cells (S+ cells) were serum-starved for 4 days (SS cells) or treated with AraC for 3 days (AraCS cells). **b** Cell counting with trypan blue staining. THP1 cells were serum-starved or treated with AraC for days indicated. Then, serum was added to SS cells while AraCS cells were resuspended in fresh media. **c** S+, SS, and AraCS cells were treated with various concentration of AraC for 3 days. Viable THP1 leukemic cells were measured by cell counting using trypan blue staining and IC_50_ values of AraC are shown. **d** Transcriptome, translatome, and proteome analyses in proliferating and G0 leukemic cells. G0 cells (AraCS, SS cells) were induced by treatment of proliferating cells (S+) with AraC or serum starvation. Total RNAs, polysome-associated mRNAs, and protein were analyzed by comparative microarray and quantitative proteomics. **e** Comparison of transcriptomic, translatomic, and proteomic changes in response to SS and 5 μM AraC treatments. **f** Comparison of AraCS and SS with leukemic stem cells (LSC) [16] in AML, dormant leukemic cells (LRC) [15], minimal residual disease (MRD) [15] in ALL, and G0 fibroblasts [1]. GSEA analysis was performed to determine whether previously published transcriptome signatures of LSC, LRC, MRD, and G0 HFF are upregulated in AraCS and SS cells, compared to S+ cells. “N” marks the limited resolution of the proteome in the GSEA. *p ≤ 0.05. Data are represented as average ± SEM. See also Additional file 1: Figure S1 and Additional file 2: Table S1

To study clinical resistance in cancer, THP1 human AML cells were used as they show significant resistance to AraC [17] (cytosine arabinoside, Additional file 1: Figure S1A), a standard anti-leukemic chemotherapeutic that targets DNA replication and thus proliferating cells (referred to as S+). Our data and others find that serum starvation of THP1 [13] and other cell lines [1, 8, 11, 18] induces a transient G0 state with known G0 and cell cycle arrest markers expressed (Fig. 1a and Additional file 1: Figure S1B-C). Such serum starvation-induced G0 cells (referred to as SS) can be returned to the cell cycle upon serum addition (Fig. 1b), verifying that they are quiescent and transiently arrested, unlike senescence or differentiation that are not easily reversed [1]. We find that serum starvation-induced G0 SS cells show resistance to AraC chemotherapy. Serum-grown S+ cells show a dose-dependent decrease in cell viability with AraC as expected, while SS cells persist, indicating their chemoresistance (Fig. 1c). Chemoresistant cancer cells include cancer stem cells and are a subpopulation that can be isolated from cancers after treatment with chemotherapy [2, 6–10] that targets and eliminates S+ cells. We find that AraC-surviving THP1 (referred to as AraCS) cells are transiently arrested, like SS cells (Fig. 1b and Additional file 1: Figure S1B); both AraCS and SS cells survive chemotherapy (Fig. 1c). AraCS cells recover from their transient arrest upon AraC removal and proliferate (Fig. 1b), affirming the reversible G0 arrest state of chemoresistant cells, similar to SS cells [1, 2, 6–10].

### G0 cells induced by SS or AraC have similar translatomes and proteome features that recapitulate gene expression profiles of in vivo chemoresistant leukemic and G0 models

To study post-transcriptionally regulated genes in G0, we profiled S+ cells, SS cells, and AraCS cells at the proteome, translatome, and transcriptome levels using multiplexed quantitative proteomics [14], microarray analysis of heavy polysome-associated mRNAs [13, 14, 19], and total RNAs respectively (Fig. 1d and Additional file 1: Figure S1D-E). Notably, we find that AraCS and SS cells show more similar gene expression profiles at the proteome and translatome levels, compared to transcriptome levels (Fig. 1e). These data suggest that although these chemoresistant G0 cells are isolated via two different methods, they exhibit a common set of translatome and proteome, which could underlie their common characteristic of chemoresistance. These data indicate the relevance of examining both the translatome and transcriptome. Time-course translatome analysis revealed that SS G0 cells that were serum-starved for short periods (4 h and 1 day) are distinct from SS G0 cells that were serum-starved for long periods (2 days and 4 days) (Additional file 1: Figure S1F). This is consistent with G0 as a continuum of assorted, arrested states [1], with temporal differences in the underlying gene expression in early G0, compared to more homogeneity at late G0. SS and AraCS cells provide sufficient material to perform concurrent translatome, proteome, and transcriptome profiling, compared to limited cells from in vivo resistance models where only transcriptomes were profiled. To test whether our G0 leukemic cells are relevant models to study chemoresistance and G0, gene expression profiles of AraCS and SS cells were compared to published transcriptome profiles of leukemia stem cells (LSC) from AML [16], dormant leukemic cells (LRC), and minimal residual disease (MRD) from chemotherapy surviving patient samples with acute lymphocytic leukemia (ALL) [15], as well as SS G0 fibroblasts (G0 HFF) [1]. Importantly, we find that these published transcriptome signatures for in vivo chemoresistance and G0 models were significantly upregulated in our SS and AraCS cells (referred to as resistant G0 leukemic cells), compared to S+ cells (Fig. 1f and Additional file 1: Figure S1G). These data indicate that our resistant G0 leukemic cells are relevant models to study post-transcriptional regulation in chemoresistance as they have similar gene expression profiles to known transcriptional profiles from in vivo chemoresistance models.

### Inhibition of canonical translation initiation in resistant G0 leukemic cells

**Fig. 2:**
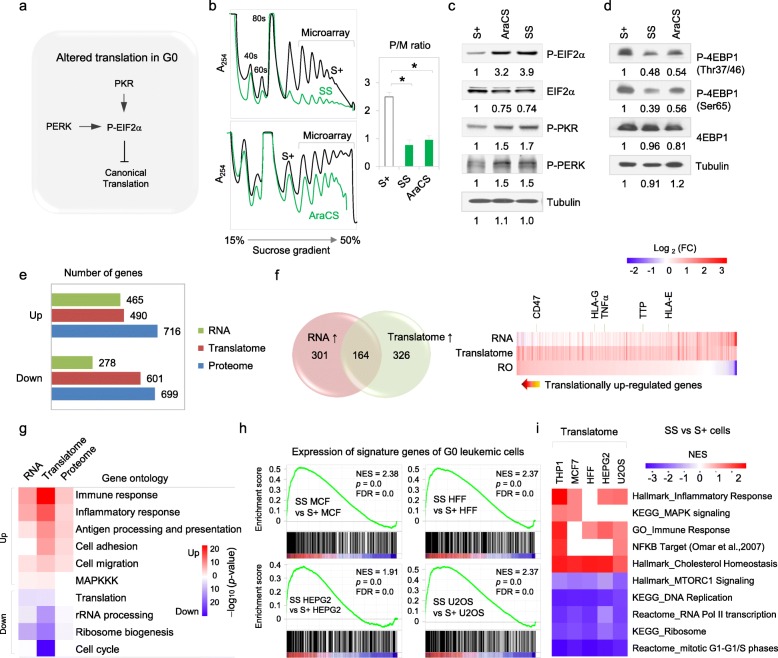
Inflammatory response mRNAs are selectively translated in G0 leukemic cells, where canonical translation is inhibited. **a** Repression of canonical translation. **b** Polysome profiles of S+, SS, and AraCS are shown. Polysome-associated mRNAs were isolated and analyzed by microarray. Graph of polysome to monosome (P/M) ratios in S+, SS, and AraCS. **c**, **d** Western analysis of translation initiation regulators: **c** eIF2α, phospho-eIF2α, and its regulators phospho-PERK and phospho-PKR, and **d** of translation regulator, eIF4EBP (4EBP) at Thr37/40 and Ser65 phosphorylation sites and total levels with quantification below. **e** Number of differentially expressed genes. **f** Venn diagram of transcriptionally and translationally upregulated genes in G0 cells induced by AraC and SS, compared to S+ cells, is shown on the left. Heatmap of gene expression changes at the transcriptome, translatome, and ribosome occupancy (RO) levels is shown on the right. See also Additional file 2: Table S1 for the 490 translationally upregulated genes and their RO changes. **g** Gene ontology (GO) analyses of differentially expressed genes shown in Fig. 2e. Statistical significance of enriched GO categories is shown as a heatmap. **h** Expression of signature genes of G0 leukemic cells in published transcriptomes of in vivo resistant leukemic and G0 models. **i** Translatome analysis of G0 cells from five different cell types. Heatmap of normalized enrichment score (NES) is shown. *p ≤ 0.05. Data are represented as average ± SEM. See also Additional file 1: Figures S1, S2 and Additional file 2: Table S1

Mechanistically, both rate-limiting steps in canonical translation initiation: recruitment of initiator tRNA, and mRNA cap recognition to recruit mRNAs to ribosomes, are inhibited in G0 leukemic cells (Fig. 2a–d) [13, 14]. We find overall protein synthesis is reduced at least twofold in AraCS, compared to S+ cells (Fig. 2b and Additional file 1: Figure S1D). Recruitment of initiator tRNA by eIF2 can be blocked by eIF2α phosphorylation as a stress response [13, 20–25]. We find that two eIF2 kinases, PKR and PERK, are activated and significantly increase eIF2α phosphorylation in SS and AraCS G0 leukemic cells (Fig. 2c, 5.3-fold (3.9/0.74) in SS and 4.2-fold (3.2/0.75) in AraCS cells of increase of phospho-eIF2α over total eIF2α, based on quantitation below the blots in Fig. 2c), which inhibits canonical translation initiation at one of the two rate-limiting steps. Consistent with our previous study [14], we observed moderate dephosphorylation in SS and AraCS G0 leukemic cells of eIF4EBP (4EBP, Fig. 2d) that can inhibit canonical translation initiation at the other rate-limiting step [26–28]. Decreased canonical translation by the above mechanistic changes can enable post-transcriptional regulation of specific genes, as observed previously [13, 14], and lead to survival of G0 leukemic cells.

Although 4EBP, a translation inhibitor that is a downstream target of mTOR signaling, is dephosphorylated in SS and AraCS cells (Fig. 2d), the effect is more pronounced for SS cells than AraCS cells (50–59% decreased phosphorylation of 4EBP in SS cells versus 31–33% in AraCS cells at two key sites; Thr37/40 and Ser65). This difference is reflected in the translation regulation of terminal oligopyrimidine tract (TOP) mRNAs, such as ribosomal protein mRNAs, that are regulated by mTOR [28–30]: TOP mRNAs are translationally decreased in SS cells (Additional file 1: Figure S1H) but not in AraCS cells. This indicates differences in SS and AraCS cells, in mTOR and other signals, which could differentially affect 4EBP [19, 28, 31] and other downstream effectors that are implicated in regulating TOP mRNAs and vary in distinct conditions [32–35]. Phosphorylation of the downstream mTOR target, RPS6 (S6), is moderately decreased in SS and AraCS leukemic cells (Additional file 1: Figure S1I, 36% in SS and 27% in AraCS cells). While the translation inhibitor, 4EBP that is downstream of the mTOR pathway, is dephosphorylated in these conditions and can partially reduce canonical translation (Fig. 2d), coordinate dephosphorylation of mTOR is not significantly observed (Additional file 1: Figure S1I) in SS (15% reduction at one site) and AraCS cells. This indicates that the mTOR pathway shows differences at different levels in SS and AraCS cells. This is likely due to feedback on mTOR from downstream S6K and other kinases, as observed in other systems [26, 36–41], while 4EBP is also known to be regulated by other kinases, independent of mTOR [42–45]. Thus, while the eIF2 pathway is strongly inhibited in SS and AraCS cells at similar levels (Fig. 2c), reducing canonical translation—and 4EBP is also moderately regulated with more dephosphorylation in SS than in AraCS cells, which could partially affect translation (Fig. 2d)—other levels of the mTOR pathway are not coordinately modulated or similarly altered in SS and AraCS cells.

### Global translatome analysis shows that inflammatory response genes are selectively translated in resistant G0 cancer cells

We measured the number of genes upregulated at the transcriptome, translatome, and proteome levels in resistant G0 leukemic cells, compared to S+ cells. A significant number of genes were upregulated in the transcriptome (465 genes), the translatome (490 genes, Additional file 2: Table S1), and proteome (716 genes) as shown in Fig. 2e. Importantly, 67% of upregulated genes were upregulated only at the translatome level (Fig. 2f) but not in the transcriptome, indicating post-transcriptional regulation. To investigate the biological function of these differentially expressed genes, gene ontology (GO) analysis was performed. Gene categories upregulated in G0 translatomes include inflammatory response (pro-inflammatory cytokines and inflammation regulators), immune response genes (immune modulators that are not pro-inflammatory cytokines or inflammation regulators, including interferon-stimulated genes, immune receptors, antigen presentation, and processing genes), cell adhesion, cell migration, lipid biosynthesis, and cholesterol pathway genes (Fig. 2g and Additional file 1: Figure S2A-B). Downregulated genes include RNA processing and ribosome genes (Fig. 2g). To identify translationally upregulated genes, we measured the change in ribosome occupancy (RO) which is the ratio of polysome-associated mRNA levels to total mRNA levels of each gene (Fig. 2f heatmap and Additional file 2: Table S1). RO values are increased for some genes, indicating translational upregulation. These genes include antigen processing and presentation genes (HLA-G) [46] and immune receptors (CD47, Fig. 2g and Additional file 1: Figure S2C) [47–49] that regulate antitumor immune response and are associated with leukemic stem cells and resistance [50, 51].

We asked if this specific gene expression profile in resistant G0 leukemic cells is conserved in G0 cells of other tumors and cell types. Therefore, global translatome profiling was conducted in G0 cells from four different cell lines: breast cancer (MCF7), liver cancer (HEPG2), and osteosarcoma (U2OS) as well as non-cancerous fibroblasts (HFF) (Additional file 1: Figure S2D-G). Their translatome profiles were compared with resistant G0 leukemic cells, using GSEA and DAVID tools (Fig. 2h, i and Additional file 1: Figure S2A). We find that the 490 signature genes (upregulated translatome) of resistant G0 leukemic cells (Additional file 2: Table S1) were highly upregulated at the translatome level in G0 cells of these other cell types (Fig. 2h). As expected for these arrested cells, genes related to cell cycle, ribosome biogenesis, and DNA replication were commonly downregulated (Fig. 2i and Additional file 1: Figure S2A). We focused on inflammatory response genes as these were commonly upregulated in G0 cells from cancer cell lines and do not significantly overlap with the senescence-associated secretory pathway (SASP) (Fig. 2i and Additional file 1: Figure S2H) [52, 53].

### Stabilization of ARE-bearing mRNAs is mediated by phosphorylation of TTP in resistant G0 leukemic cells

**Fig. 3:**
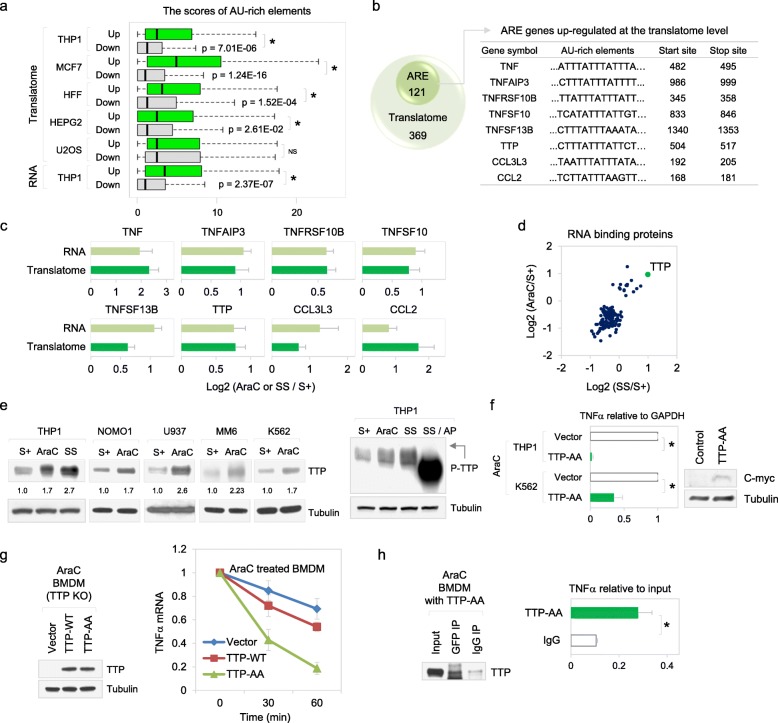
Phosphorylation of TTP stabilizes ARE-bearing TNFα in G0 leukemic cells. **a** Boxplot of ARE scores (SI methods) in the 3′UTRs of genes which are up- or downregulated at the translatome or RNA levels in G0 compared to S+ cells. **b** Venn diagram of genes that are upregulated at the translatome level and contain AREs (left) and examples of such genes (right). See also Additional file 3: Table S2 for a full list of genes. **c** Expression of ARE genes at the RNA and translatome levels. **d** Scatter plot showing the expression of RNA-binding protein genes from RBPDB database (SI methods). TTP is indicated with a green dot. **e** Western analysis of TTP in lysates from multiple leukemic cell lines in the absence or presence of alkaline phosphatase (AP). Phospho-TTP is indicated with an arrow. **f** Bar graph shows TNFα mRNA expression normalized to GAPDH mRNA upon overexpression of vector or c-myc tagged non-phosphorylatable mutant TTP (TTP-AA) in AraC-treated THP1 or K562 cells. Western analysis of TTP-AA with c-myc antibody (right). **g** Half-life of TNFα mRNA. TTP-deficient BMDM cells were transduced with doxycycline inducible plasmids that express GFP vector, TTP wild-type, or TTP-AA mutant. Cells were induced with 1 μg/ml doxycycline prior to 1 μM AraC treatment. Western analysis of induction of TTP protein. TNFα mRNA level was measured at indicated time points by qPCR after transcriptional arrest with 5 μg/ml actinomycin D treatment. **h** Association of TTP-AA with TNFα mRNA in AraCS cells. TTP-AA was immunoprecipitated with GFP antibody from AraC-treated BMDM cells expressing GFP-tagged TTP-AA (Western blot), followed by qPCR analysis of TNFα mRNA (graph). *p ≤ 0.05. Data are represented as average ± SEM. See also Additional file 1: Figure S3 and Additional file 3: Table S2

To identify *cis*-acting elements that mediate post-transcriptional regulation, the untranslated regions (UTRs) of differentially expressed genes were examined. We find that a GC-rich motif was enriched on 5′UTRs of translationally upregulated genes and an AU-rich motif, on 5′UTRs of downregulated genes, indicating that mRNAs with structured 5′UTRs are highly translated in G0 cells (Additional file 1: Figure S3A-B). Importantly, 3′UTR AU-rich elements (AREs) are significantly enriched in the upregulated translatome as well as transcriptome (Fig. 3a). Furthermore, 25% of the translatome signature of G0 leukemic cells bear AREs (Additional file 3: Table S2), including pro-inflammatory cytokines such as TNFα and chemokines (Fig. 3b, c) according to the ARE database [54]. AREs are important post-transcriptional regulatory elements that mediate rapid degradation and repression of mRNAs [30]. To understand how ARE mRNAs are highly expressed in G0 cells, we assessed the expression level of RNA-binding proteins. As expected, most ARE-binding proteins known to cause mRNA decay or translation repression [55, 56] are significantly reduced in G0 cells (Additional file 1: Figure S3C-D). Additionally, the exosome and proteasome complexes that are implicated in ARE mRNA decay [57, 58] are reduced (Additional file 1: Figure S3E-F). However, a key ARE mRNA decay factor, Tristetraprolin (TTP), was surprisingly increased in AraCS from multiple AML cell lines (Fig. 3d, e). However, we find that TTP is phosphorylated in SS and AraCS cells (Fig. 3e, right blot). TTP phosphorylation is established to increase its levels [59] and blocks its ability to destabilize ARE mRNAs, thus enabling ARE mRNA translation upon lipopolysaccharide (LPS) treatment in immune cells [60, 61]. To test whether phosphorylation of TTP was required for the increased expression of ARE mRNAs in G0 leukemic cells, we generated non-phosphorylatable mutant TTP with key phosphorylation sites (Ser 52, 178) replaced by alanine (TTP-AA). TTP-AA has been shown to mediate ARE mRNA decay activity and reduce pro-inflammatory cytokines like TNFα in immune cells, as it cannot be phosphorylated and inactivated [59–61]. Expression of myc-tagged TTP-AA significantly reduced TNFα mRNA in both THP1 and K562 AraCS cells (Fig. 3f), as it restored a form of TTP that cannot be phosphorylated and can pursue its decay function, unlike the endogenous TTP that gets phosphorylated and inactivated for its decay function. To determine the effect of TTP phosphorylation on the stability of ARE mRNAs, we measured the half-life of TNFα mRNA. Expression of TTP-AA mutant reduced the half-life of TNFα mRNA more significantly than TTP wild-type (TTP-WT) expressed in AraC-treated TTP-deficient cells (Fig. 3g). We compared the G0 translatome and transcriptome with TTP-CLIP datasets [61] to identify how many G0-expressed genes are TTP targets. The upregulated G0 translatome and RNA profiles (166 out of 490 translatome genes with *p* < 2.71e−21; 174 out of 465 RNA profile genes with *p* < 1.322e−26), and those with AREs (49–53%; 59 out of 121 translatome genes with *p* value < 7.302e−16; 75 out of 142 RNA profile genes with *p* < 1.535e−22) include known TTP targets (Additional file 1: Figure S3G, using hypergeometric probability test). Furthermore, immunoprecipitation demonstrated that TTP-AA was associated with TNFα mRNA in AraCS cells (Fig. 3h, GFP-tagged TTP-AA). In addition, to determine how many genes are regulated by TTP phosphorylation, we profiled AraC-treated cells that lack endogenous TTP but stably express TTP-AA or TTP-WT at the RNA level [61]. Expression of TTP-AA mutant downregulated 58 genes at the mRNA level compared to TTP-deficient cells. TTP-CLIP data suggests that 40% of mRNAs affected by TTP-AA (23 out of 58, *p* < 3.585e−05, Additional file 1: Figure S3Hi) are associated with TTP. The other RNAs may be indirect targets, not directly associated with TTP. Furthermore, these mRNAs are stabilized by phosphorylation of TTP; expression of TTP-WT that allows TTP phosphorylation upregulates 53% of genes downregulated by TTP-AA (31 out of 58, *p* value < 0.05, fold change > 1.5, Additional file 1: Figure S3Hii and S3Hiii). Of these 58, at least 18 genes have AREs that are recorded in the ARE database [54] and are also stabilized by phosphorylation of TTP (S3Hiv). These data indicate that inactivation of the ARE mRNA decay function of TTP by TTP phosphorylation [59, 61, 62] is a key regulator of expression of a pro-inflammatory gene, TNFα, in chemoresistant G0 cells. These results are consistent with our findings of increased levels and translation of ARE-bearing mRNAs due to decreased ARE mRNA decay activity in G0 cells (Fig. 3a–c and Additional file 1: Figure S3C-F).

### The p38 MAPK-MK2 pathway phosphorylates TTP to promote expression of ARE-bearing mRNAs in resistant G0 leukemic cells

**Fig. 4:**
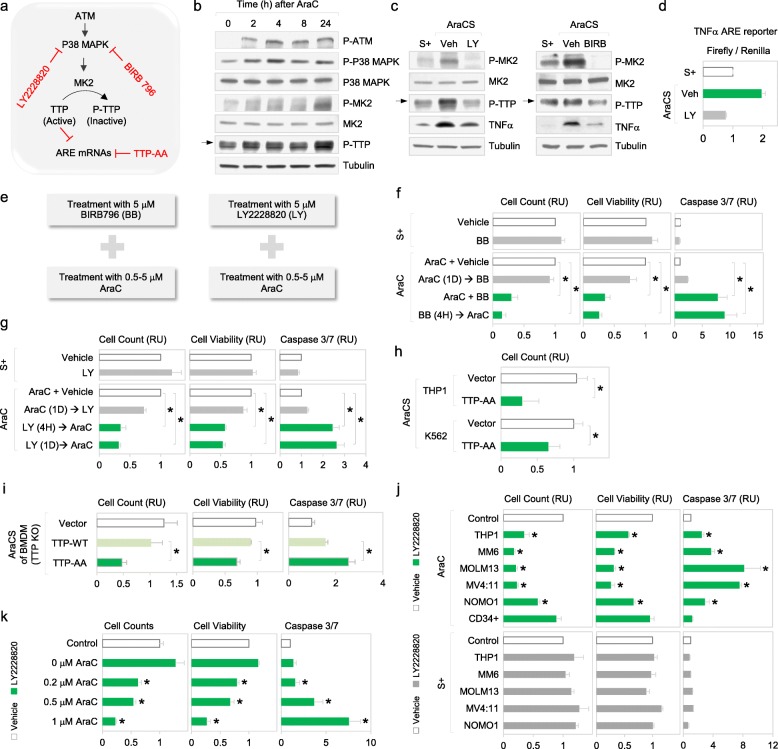
Phosphorylation of TTP by p38 MAPK-MK2 promotes chemoresistance. **a** The p38 MAPK (p38)-MK2 pathway enables stabilization and translation of ARE-bearing mRNAs via TTP phosphorylation and inactivation of its RNA decay function, in chemoresistant G0 cells. LY2228820 (LY) and BIRB396 (BB or BIRB) are p38 inhibitors. **b** Western analysis of in lysates from THP1 cells at indicated time points after AraC treatment. **c** Western analysis in S+ and AraCS cells treated with vehicle, 5 μM LY or 5 μM BB for 3 days. **d** Firefly luciferase activity of a reporter bearing TNFα ARE in its 3′UTR normalized to activity of co-transfected Renilla luciferase in S+ and AraCS cells treated with either vehicle, or 5 μM LY. **e** Sequential treatment with p38 inhibitors and AraC in leukemic cells. **f**, **g** Effect of p38 inhibition on survival of AraC-resistant cells after indicated treatments normalized to DMSO treatment (represented as a white bar); THP1 cells were treated with 5 μM BB, 5 μM LY, and vehicle in the absence (S+, top panels) or presence (AraC, bottom panels) of 5 μM AraC treatment for 3 days. Bar graphs show relative cell viability and death assessed by cell counting, MTS, and caspase 3/7 assays. In the presence of AraC, THP1 cells were treated with p38 inhibitors prior to AraC treatment (BB → AraC, LY → AraC), at the same time with AraC (AraC + BB) and 1 day after AraC (AraC → BB, AraC → LY). 4H and 1D indicate 4 h and 1 days, respectively. RU = relative units. **h**, **i** Effect of TTP-AA mutant on survival of AraC-resistant cells. TTP-AA mutant expression prior to 5 μM AraC treatment, which decreased TNFα in THP1 or K562 cells in Fig. 3f. Cell viability was assessed by cell count (H). TTP-AA, TTP wild-type, and vector were expressed in TTP-deficient BMDM cells prior to 1 μM AraC treatment. Bar graphs show relative cell viability and death (**i**). **j** Effect of p38 inhibition on resistant cells from five AML cell lines (M5 FAB subtype) after indicated treatments normalized to DMSO treatment for each cell line (represented as a white bar and set to 1). Cells were treated with 5 μM LY or vehicle 4 h prior to AraC treatment (top panel, AraC) or in the absence of AraC (bottom panel, S+). Human CD34+ cells from healthy donors were tested as a control. **k** Effect of p38 inhibition on survival of chemoresistant cells induced with various concentrations of AraC. MV4:11 leukemic cells were treated with 5 μM LY or vehicle prior to 0 μM, 0.2 μM, 0.5 μM, or 1 μM AraC for 3 days. *p ≤ 0.05. Data are represented as average ± SEM. See also Additional file 1: Figure S4

To investigate how TTP is phosphorylated in resistant G0 leukemic cells, we examined key signaling molecules involved in DNA-damage response (DDR) (Fig. 4a) that is induced by chemotherapies like AraC [63–66]. As expected, AraC treatment induced rapid phosphorylation and activation of ATM (Fig. 4b and Additional file 1: Figure S4A). Importantly, we find that these conditions lead to phosphorylation and activation of p38 MAPK and its downstream effector, MAPKAPK2 (MK2) [67, 68] (Fig. 4b). MK2 has been shown to phosphorylate TTP in macrophages treated with LPS [59, 61, 62]. To examine whether the p38 MAPK-MK2 pathway phosphorylates TTP in resistant G0 leukemic cells, two different inhibitors of p38 MAPK were tested. Treatment with p38 MAPKα/β inhibitor, LY2228820 (LY) [68, 69], or a pan-p38 MAPK inhibitor that targets all isoforms, BIRB796 (BIRB) [70], blocked phosphorylation of MK2 and prevented MK2-mediated TTP phosphorylation and reduces TNFα in AraCS cells (Fig. 4c). These results suggest that p38 MAPK-MK2 phosphorylates and inactivates TTP, resulting in enhanced expression of ARE mRNAs such as TNFα upon AraC treatment (Fig. 4a). To test if the p38 MAPK-MK2-TTP pathway regulates TNFα expression via its ARE, a firefly luciferase reporter bearing the 3′UTR ARE of TNFα, and as control, Renilla luciferase, were co-transfected. Luciferase activity of the ARE reporter increased by twofold in AraCS cells compared to S+ cells but not when p38 MAPK was inhibited (Fig. 4d). These data suggest that the p38 MAPK-MK2-TTP axis upregulates expression of specific genes via AREs in G0 leukemic cells.

### Phosphorylation of TTP induced by p38 MAPK-MK2 promotes chemoresistance

We noted that the p38 MAPK-MK2 pathway was rapidly activated to phosphorylate TTP within 1 day of SS or AraC treatment (Fig. 4b and Additional file 1: Figure S4A-B). To test the effect of inhibition of TTP phosphorylation on chemoresistance, p38 MAPK was inhibited *before* (or along with) as well as *after* treatment with AraC—and then, chemosurvival was measured using multiple assays, including cell death and two cell viability assays (Fig. 4e–g). Inhibition of p38 MAPK with BIRB or LY, 1 day *after* AraC treatment, when TTP was already phosphorylated, did not show any significant reduction in survival of AraC-resistant cells (Fig. 4f, g). Conversely, inhibition of p38 MAPK at earlier time points prior to AraC treatment, when TTP was not phosphorylated, increased apoptosis, and reduced survival of AraC-resistant cells (Fig. 4f, g). As a control, p38 MAPK inhibition alone does not affect viability of S+ cells that are not treated with AraC (Fig. 4f, g). These results suggest that p38 MAPK is rapidly activated upon AraC treatment to turn on downstream survival pathways such as phosphorylation of TTP. Thus, to inhibit phosphorylation of TTP and hence overcome AraC resistance effectively, p38 MAPK needs to be targeted at early time points.

To confirm that phosphorylation of TTP induces chemoresistance, we overexpressed TTP mutant (TTP-AA) that cannot be phosphorylated by p38 MAPK-MK2, followed by AraC treatment. Importantly, we find that TTP-AA mutant expression reduces survival of AraC-resistant cells in THP1 and K562 leukemic cell lines (Fig. 4h). Furthermore, TTP-AA mutant, expressed in TTP-knockout macrophages, induced apoptosis of AraC-surviving cells more significantly, compared to TTP wild-type (Fig. 4i). Consistently, in multiple AML cell lines, early inhibition of p38 MAPK showed dramatically reduced chemosurvival but not in non-cancerous CD34+ cells (Fig. 4j). When treated with p38 MAPK inhibitor alone, viability of S+ cells in multiple AML cell lines remained unchanged, indicating the synergism of AraC and p38 MAPK inhibitors (Fig. 4j). Interestingly, p38 MAPK inhibition eliminated resistant cells more significantly at increasing concentrations of AraC (Fig. 4k). This indicates that treatment with high concentrations of AraC would increase the number of cells induced into the resistant G0 state with strong phosphorylation of p38 MAPK-MK2-TTP. Conversely, even low concentrations of BIRB were sufficient to reduce chemoresistance (Additional file 1: Figure S4C). Unlike in solid tumors, where activation of p38 MAPK-MK2 induces resistance by arresting the cell cycle [30, 67, 68], p38 MAPK inhibition did not affect the cell cycle in AML cells (Additional file 1: Figure S4D). These data uncover rapid activation of a p38 MAPK-MK2 pathway that enables chemosurvival of G0 leukemic cells via inhibition of TTP activity.

### TNFα, induced by phosphorylation of TTP, promotes chemoresistance

**Fig. 5:**
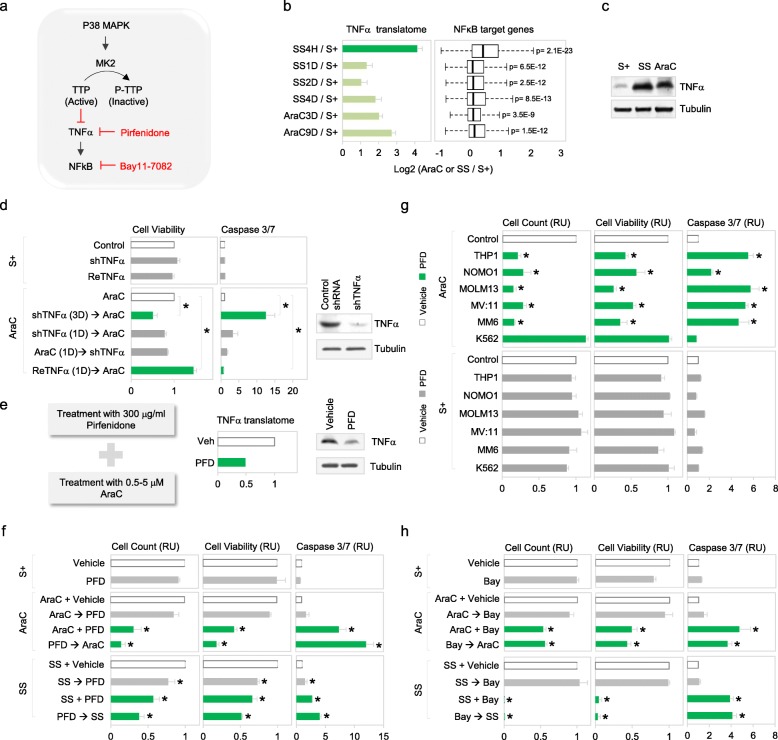
TNFα induced by phosphorylation of TTP promotes chemoresistance. **a** Phosphorylation of TTP by the p38-MK2 pathway inactivates its RNA decay function, which leads to stabilization of ARE-bearing TNFα mRNA, resulting in activation of NF-kB signaling in resistant G0 leukemic cells. TNFα expression is inhibited by TTP-AA mutant, pirfenidone (PFD) or shRNAs, and NF-kB signaling by NF-kB inhibitor, Bay11-7082. **b** Expression of TNFα and NF-kB target genes at the translatome level at indicated time points after SS or AraC treatment. **c** TNFα protein level in S+, SS, and AraCS cells. **d** Effect of TNFα on chemoresistance. THP1 cells were transduced with doxycycline inducible shRNA against TNFα or control shRNA. ShRNA against TNFα was induced prior to AraC (shTNFα → AraC) or after AraC (AraC → shTNFα) and recombinant TNFα protein was added 1 day prior to AraC (ReTNFα → AraC). Cell viability and western analysis of TNFα are shown. **e** Effect of 300 μg/ml of PFD treatment for 3 days on TNFα expression at the translatome (middle) and protein levels (right) in AraCS cells. **f** Effect of pharmacological inhibition of TNFα by PFD on AraC resistance. THP1 cells were treated with 300 μg/ml PFD or vehicle in the absence of AraC (S+, top panels), in the presence of AraC (AraC, middle panels), or on serum starvation (SS, bottom panels). Bar graphs show cell viability and death assessed by cell counting, MTS, and caspase 3/7 assays. In the middle or bottom panels, THP1 cells were treated with PFD 1 day prior to AraC or SS (PFD → AraC, PFD → SS), at the same time with AraC or SS (AraC + PFD, SS + PFD), and 1 day after AraC or SS (AraC → PFD, SS → PFD). **g** Effect of TNFα inhibition on AraC resistance from six different leukemic cell lines. Cells were treated with PFD or vehicle 1 day prior to AraC (AraC, top panels) or in the absence of AraC (bottom panels, S+). **h** Effect of NF-kB inhibition on AraC resistance. THP1 cells were treated with 10 μM Bay 11-7082 (Bay) or vehicle in the absence of AraC (S+, top panels), in the presence of AraC (AraC, middle panels) or under serum starvation (SS, bottom panels). In the middle or bottom panels, THP1 cells were treated with Bay11-7082, 1 day prior to AraC or SS (Bay → AraC, Bay → SS), at the same time with AraC or SS (AraC + Bay, SS + Bay), and 1 day after AraC or SS (AraC → Bay, SS → Bay). *p ≤ 0.05. Data are represented as average ± SEM. See also Additional file 1: Figure S5

We demonstrated that TTP inactivation in SS and AraCS cells regulates the stability of ARE mRNAs such as TNFα in AraCS cells (Figs. 3g and 5a). This allowed such resistant G0 leukemic cells to show elevated TNFα translatome and protein levels (Fig. 5b, c). To assess the effect of TNFα on chemoresistance, we altered TNFα levels genetically and phamacologically in G0 cells. Induction of TNFα depletion prior to AraC effectively reduced AraC resistance, compared to depleting TNFα after AraC treatment, while no effect was observed with TNFα depletion alone without AraC (Fig. 5d). In contrast, addition of recombinant TNFα enhanced survival of AraCS cells (Fig. 5d). TNFα-mediated chemoresistance is not due to arrested cell cycle as TNFα treatment without subsequent AraC does not alter the cell cycle (Additional file 1: Figure S5A). These data suggest that phosphorylation of TTP and subsequent expression of TNFα, which are induced by p38 MAPK-MK2, are responsible for survival of G0 leukemic cells.

TNFα can also be inhibited pharmacologically with the drug pirfenidone (PFD) that can block TNFα translation in RAW264.7 cells and is used to treat idiopathic pulmonary fibrosis [68, 71, 72]. In G0 leukemic cells, PFD reduced TNFα translatome and protein levels but not mRNA levels (Fig. 5e and Additional file 1: Figure S5B). PFD treatment at least 18 h *prior to* or along with AraC or SS significantly reduced viability of G0 leukemic cells but failed to reduce resistance when added *after* AraC treatment (Fig. 5f and Additional file 1: Figure S5C). As observed with p38 MAPK-MK2 activation (Fig. 4a, b), TNFα translatome level also is rapidly and dramatically increased upon SS treatment (Fig. 5b). These data indicate that activation of TNFα is an early event in G0 induction, which leads to resistance, and needs to be inhibited early to preclude downstream survival regulators. PFD treatment alone does not affect the viability of untreated S+ cells, indicating that the cytotoxic effect of PFD is specific to G0 leukemic cells (Fig. 5f). PFD treatment reduced chemotherapy survival in multiple AML cell lines (Fig. 5g). Similar results were observed in MCF7 cells, where PFD reduced doxorubicin resistance (Additional file 1: Figure S5D).

TNFα activates the NFκB pathway that increases anti-apoptotic gene expression to promote cell survival [73–75]. Our observation of early activation of p38 MAPK-MK2 (Fig. 4a, b) suggested that TNFα could be rapidly upregulated upon G0 induction. Time-course translatome analysis affirmed that TNFα is highly increased (16-fold) at the earliest time point of 4 h after serum starvation or AraC treatment (Fig. 5b) along with its receptors, leading to rapid elevation of downstream NFκB target genes including antiapoptotic BCL family members [75–77] (Fig. 5b and Additional file 1: Figure S5E-F). Similar to our observations with TNFα inhibitor PFD (Fig. 5f), NFκB inhibitor, BAY11-7082 [78], prior to or along with AraC or SS decreases the viability of G0 cells, while treatment after AraC or SS had no effect (Fig. 5h). TNFα shRNA (Fig. 5d) or inhibition (Fig. 5f), and NFkB inhibition (Fig. 5h), effectively reduce survival of resistant cells as noted by viability assays. Apoptosis or caspase 3/7 activity mediated by TNFα shRNA or TNFα inhibitor, PFD, and NFkB inhibitor, BAY 11-7082, vary, although they all cause significant decrease in chemoresistant cell viability. While other pathways downstream of TNFα could affect apoptosis [79, 80], the differences in caspase activity can be due to differences in inhibition by shRNA depletion versus drug effects, as BAY 11-7082 can mediate NFκB-independent pathways and non-apoptotic cell death mechanisms [81, 82]. These data suggest that the TNFα-NFκB inflammatory pathway is upregulated as an early survival pathway in G0 cells.

### TTP regulates a pro-apoptotic JNK pathway via targeting DUSP1

**Fig. 6:**
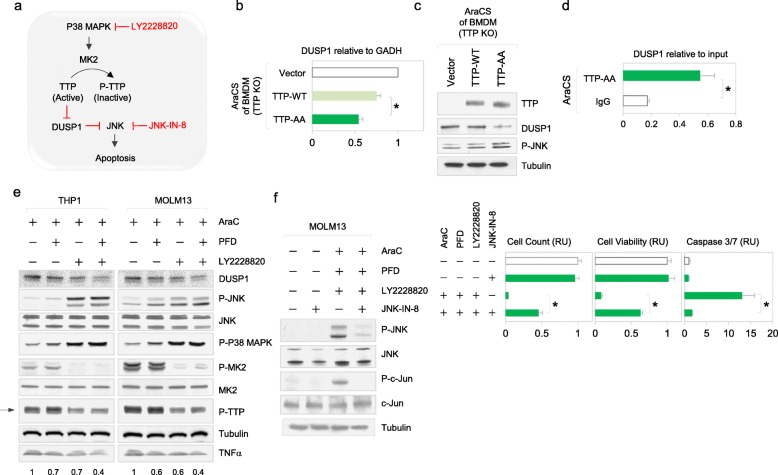
TTP regulates a pro-apoptotic JNK pathway via targeting DUSP1. **a** Phosphorylation of TTP allows expression of the ARE-bearing mRNA of DUSP1 that inhibits JNK and hence blocks JNK-mediated apoptosis. JNK pathway is blocked by the inhibitor JNK-IN-8. **b**–**d** Effect of TTP-AA mutant on DUSP1 and phosphorylation of JNK. BMDM TTP-deficient cells were treated with doxycycline to express TTP-AA and TTP wild-type prior to AraC treatment. **b** DUSP1 mRNA level was measured by qPCR and is shown relative to GAPDH mRNA. **c** Western analyses of TTP, DUSP1, and phospho-JNK are shown. **d** TTP-AA (GFP tagged) was immunoprecipitated with GFP antibody, followed by qPCR analysis for DUSP1 mRNA. **e** Western analyses in THP1 and MOLM13 cells treated with indicated drug combinations for 1 day (150 μg/ml PFD and 2.5 μM LY2228820, which are half the amounts used in Figs. 4g and 5f). Phospho-TTP is indicated with an arrow and quantitation of TNFα protein is shown below. **f** JNK pathway mediates apoptosis. MOLM13 cells treated with indicated drug combinations. JNK pathway was inhibited with 1 μM JNK-IN-8. Western analyses of phospho-JNK, phospho-c-Jun, and c-Jun shown on the left; associated cell viability and death graphed on the right. Data are represented as average ± SEM

We asked what other ARE mRNAs are targeted by TTP and affect cell survival. DUSP1 mRNA contains AREs in its 3′ UTR. TTP has been shown to target DUSP1 mRNA for degradation upon LPS treatment of macrophages or dendritic cells [60, 61, 83]. Consistently, DUSP1 in AraC treatment in both THP1 and MOLM13 cells is decreased upon treatment with BIRB (Additional file 1: Figure S5G), indicating its regulation by p38 MAPK. To determine if TTP phosphorylation regulates DUSP1 in AraCS, we expressed TTP-AA mutant that is not phosphorylated in cells that lack TTP (Fig. 6a). Expression of TTP-AA mutant more significantly reduced DUSP1 mRNA and protein levels compared to cells expressing TTP wild-type (Fig. 6b, c). Immunoprecipitation showed that TTP-AA associated with DUSP1 mRNA in AraCS cells (Fig. 6d), similar to its association with TNFα mRNA (Fig. 3h). Furthermore, inhibition of phosphorylation of TTP by p38 MAPK inhibitor decreased DUSP1 protein level (Fig. 6e). DUSP1 is a MAPK phosphatase which dephosphorylates JNK [84]. In AraCS cells, DUSP1 protein level is negatively correlated with phosphorylated JNK (Fig. 6e), consistent with DUSP1-mediated suppression of JNK [84]. To determine the effect of JNK on survival of leukemic cells, JNK inhibitor, JNK-IN-8 was used (Fig. 6a). Importantly, JNK inhibition reversed apoptosis of leukemic cells treated with AraC, LY and PFD, but did not affect the viability of untreated cells (Fig. 6f, graph), indicating that inhibition of JNK pathway contributes to chemoresistance. Together, these results suggest that TTP-DUSP1 axis promotes chemoresistance via suppressing JNK-mediated apoptosis (Fig. 6a).

### Co-inhibition of p38 MAPK and TNFα sensitizes resistant leukemic cells to AraC treatment

**Fig. 7:**
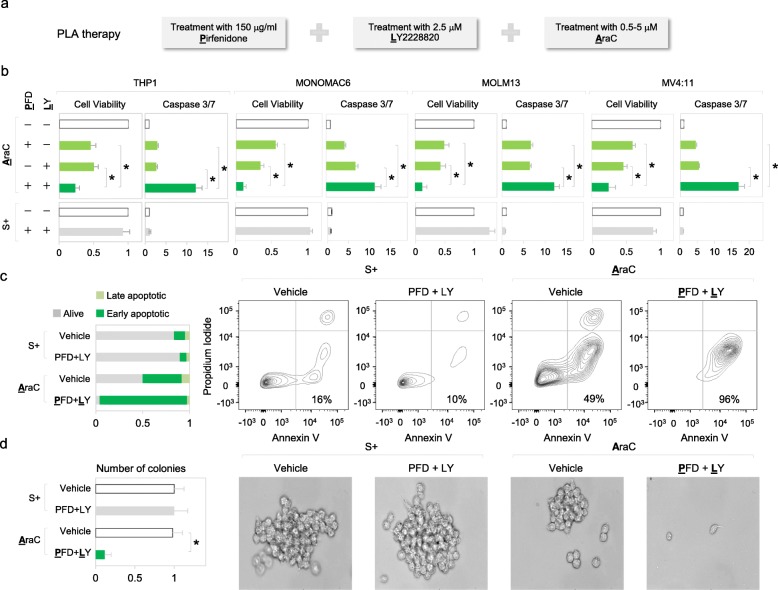
PLA therapy decreases AraC-resistant cells in AML cell lines. **a** PLA therapy, involves pre-treatment of leukemic cells with *P*FD and *L*Y followed by *A*raC treatment, using half of the concentrations used for individual drugs in Figs. 4g and 5f. **b** Three different AML cell lines apart from THP1 were sequentially treated with indicated drugs, followed by assessment of cell viability and death. **c**, **d** Viability of MOLM13 cells treated with indicated drug combinations. Flow cytometric profiles of cells stained with annexin V and propidium iodide are shown (**c**). Cells were plated on methylcellulose media for colony formation, to test survival in the presence of drug combinations. Representative colony images and quantification of colonies are shown (**d**). *p ≤ 0.05. Data are represented as average ± SEM. See also Additional file 1: Figure S5

Although chemoresistant cells are sensitive to individual inhibition of either TNFα or p38 MAPK by PFD or LY respectively, a substantial proportion of cells still survived (Figs. 4g and 5f). Therefore, we asked if co-inhibition of p38 MAPK and TNFα with LY *and* PFD respectively, could eliminate the remaining resistant cells (Fig. 7a). We find that individual treatment with either of LY or PFD (at half the dosages used in Figs. 4g and 5f) prior to or along with AraC, reduces approximately 50% of surviving leukemic cells (Fig. 7b). Importantly, this combination of *P*FD and *L*Y2228820 prior to *A*raC treatment—called PLA therapy—eliminates about 90% of chemoresistant cells in multiple AML cell lines and not just THP1 cells (Fig. 7a–c). Furthermore, PLA therapy decreased colony formation of leukemic cells on methylcellulose by 10-fold, compared to AraC treatment alone (Fig. 7d). These data indicate decreased survival of leukemic cells treated with PLA therapy. In contrast, in the absence of AraC treatment, the combination of PFD and LY2228820 did not affect cell viability, apoptosis and colony formation, indicating the synergistic effect between AraC and anti-inflammatory drugs (Fig. 7b–d). Despite the fact that stromal niche cells have been shown to protect leukemic cells from chemotherapy [85], we find that AML cells co-cultured with stromal cells remained sensitive to PLA therapy (Additional file 1: Figure S5H). We examined the molecular mechanism by which PLA therapy enhanced chemosensitivity. We find that LY treatment destabilizes TNFα mRNAs by TTP dephosphorylation [59] (Figs. 3g and 4c), while PFD suppresses translation of TNFα mRNA [72] (Fig. 5e and Additional file 1: Figure S5B). Therefore, in PLA therapy, TNFα remains more effectively blocked, compared to individual drug treatments (Figs. 6e and 7b). Furthermore, a pro-apoptotic JNK pathway was more significantly activated in cells treated with PLA therapy than single-drug treatments (Fig. 6e). Together, these results suggest that PLA therapy reduces TNFα and promotes a pro-apoptotic JNK pathway, leading to apoptosis of chemoresistant cells.

### PLA therapy reduces chemoresistance in primary AML cells ex vivo and in vivo

**Fig. 8:**
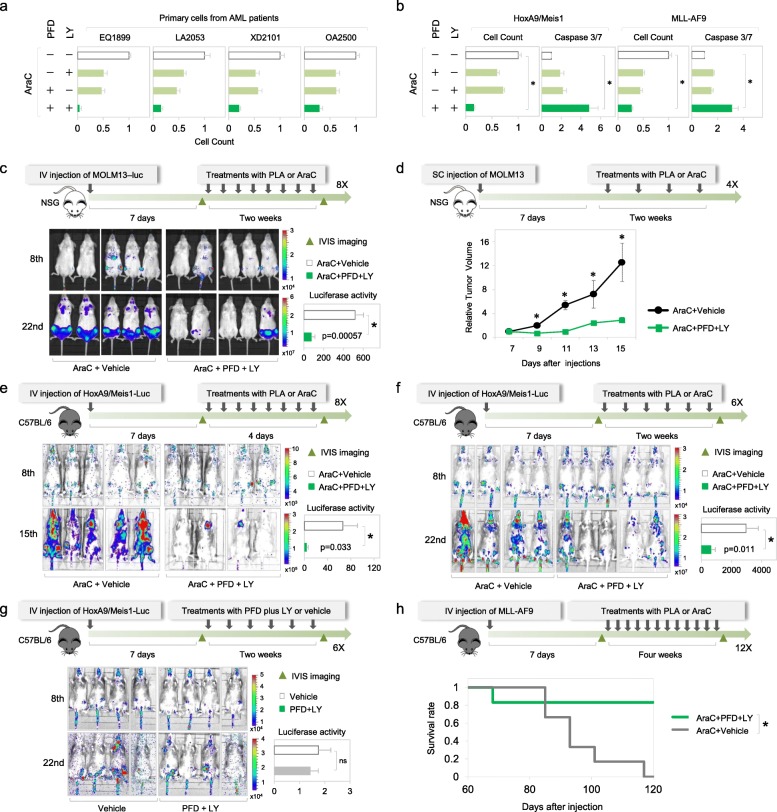
PLA therapy significantly reduces AraC resistance in primary AML cells ex vivo and in vivo. **a** Viability of primary cells from four AML patients after indicated treatments normalized to vehicle+AraC treatment for each patient sample (represented as a white bar and set to 1); other patient samples and combinations as well as normal CD34+ cells from healthy donors after indicated treatments are shown in Additional file 1: Figure S5I. **b** Viability and death of primary cells from AML mouse models driven by HoxA9/Meis1 and MLL-AF9 after indicated treatments. **c–g** Bioluminescence images and quantification of tumor growth in NSG mice engrafted with MOLM13 cells and at the indicated days after engraftment, were treated with PLA therapy or AraC (**c**, **d**) and in C57BL/6 mice engrafted with primary HoxA9-Meis1/luciferase cells and treated with PLA therapy or AraC with two different dosage schedules (8e: treated 8 times with drugs over 4 days versus 8f: 6 times with drugs over 2 weeks) or treated with PFD plus LY or vehicle as a control (**g**). **h** Kaplan-Meier survival curves of MLL-AF9 engrafted C57BL/6 mice, treated with PLA therapy or AraC. The number of drug injections in **c**–**h** is marked: 8X (**c**, **e**), 4X (**d**), 6X (**f**, **g**), and 12X (**h**). For **c**, **e**–**g**, relative luciferase activity was quantified and plotted as bar graphs to represent tumor survival. *p ≤ 0.05. Data are represented as average ± SEM. See also Additional file 1: Figures S5-S6

To test the anti-leukemic activity of PLA therapy in primary AML [86], primary cells from AML patients (Fig. 8a and Additional file 1: Figure S5I) as well as two murine AML models driven by Hoxa9/Meis1 or MLL-AF9 (Fig. 8b), were used. When either p38 MAPK or TNFα was inhibited prior to AraC treatment, moderate apoptosis of chemoresistant cells was observed in primary AML cells (Fig. 8a, b and Additional file 1: Figure S5I). Importantly, co-inhibition of p38 MAPK and TNFα by PLA therapy (pre-treatment before AraC) significantly reduced AraC resistance in AML patient samples (Fig. 8a and Additional file 1: Figure S5I) as well as in primary cells from two AML mouse models ex vivo (Fig. 8b). In contrast, the viability of normal CD34+ cells from healthy donors was not affected by treatment with LY or PFD (Fig. 4j and Additional file 1: Figure S5I), consistent with clinical studies that have shown that PFD and LY have acceptable safety and tolerance [69, 71]. To further investigate the therapeutic potential of PLA therapy in vivo, human AML cells expressing luciferase (MOLM13-Luc) were intravenously or subcutaneously injected into NSG mice. After confirmation of engraftment by measuring tumor volume or bioluminescent imaging (BLI), the mice were treated with PLA therapy or AraC for two weeks. Consistent with ex vivo results (Fig. 7b), PLA therapy significantly decreased the leukemic burden and tumor volume by 6-fold, compared to AraC treatment alone (Fig. 8c, d). Next, primary Hoxa9/Meis1 or MLL-AF9 leukemia cells were generated as described previously [87], and transplanted to second recipient mice. These mice were treated with PLA therapy or AraC, with two different dosage schedules. Consistently, BLI shows that PLA therapy eliminated 78% or 96% of chemoresistant cells in a dosage-dependent manner (Fig. 8e, f; 8 times with drugs over 4 days versus 6 times with drugs over 2 weeks). In the absence of AraC treatment, the combination of PFD and LY2228820 did not affect leukemic burden, suggesting that cytotoxic effects of this combination are limited to AraC-resistant cells, rather than proliferating cells (Fig. 8g). Correspondingly, PLA therapy extended mice survival (Fig. 8h and Additional file 1: Figure S5J). Together, these results suggest PLA therapy has potential for improving AraC-mediated apoptosis in AML.

## Discussion

G0 cells are a transiently arrested, clinically relevant subpopulation in cancers [1, 2, 5–10]. Our previous data and others, revealed altered gene expression mechanisms in G0 leukemic cells, at the post-transcriptional [8, 12] and translational levels [13, 14, 18]. This would lead to a distinct gene expression profile to enable G0 cell survival in harsh conditions. G0 cells are resistant to stress conditions like serum starvation, with transient inhibition of apoptosis, and proliferation [1, 11, 18]. Importantly, we find that serum-starved leukemic SS G0 cells exhibit chemoresistance (Fig. 1c); consistently, true chemosurviving AraCS cells are transiently arrested and chemoresistant (Fig. 1a, b and Additional file 1: Figure S1B). In accord, we find that SS cells are similar in translatome and proteome to AraCS cells (Fig. 1e), indicating that consistent with their common features of G0 arrest and chemosurvival, they show similar post-transcription gene expression. Published transcriptional signatures of in vivo chemoresistance leukemic models [1, 2, 8, 11, 15, 16], are also highly expressed in SS and AraCS cells (Fig. 1f and Additional file 1: Figure S1G). Thus, the common G0 resistance gene expression profile observed in AraCS and SS G0 cells likely comprises genes that control survival and resistance. These data revealed that in addition to known transcriptional profiles, altered post-transcriptional mechanisms in G0 resistant cells contribute to their unique gene expression profile that underlies their chemoresistance.

Our findings reveal the importance of DNA damage and stress signaling that can initiate a pro-inflammatory response that causes survival (Fig. 4). Differential genomic instability in cancers would lead to subpopulations within a tumor with disparate DDR and stress signaling [63–65] that we find, enables their chemotherapy survival via pro-inflammatory cytokines. Cytokines upregulated in SS and AraCS cells include some SASP factors but also other unique cytokines [52, 53] (Additional file 1: Figure S2H). This is consistent with similarities and differences between G0 and senescence [1]: both show inhibition of the cell cycle but—unlike in senescence—G0 shows reversible cell cycle arrest (Fig. 1a, b and Additional file 1: Figure S1B-C), increased stem cell markers (Fig. 1f and Additional file 1: Figure S1G), markers of maintenance of G0 such as HES1 (Additional file 1: Figure S1C, S2D) [14] that are not expressed in senescence [1], low p53 [18], and lack of common senescence markers (Additional file 1: Figure S2H) [18, 52, 53]. These data indicate that a quiescence- and resistance-specific set of pro-inflammatory and signaling genes are expressed in these resistant cells (Fig. 2g). These include inflammatory cytokine, TNFα, & its receptors that promote downstream NFκB activated pro-survival target genes [73–75] including BCL family members of antiapoptotic genes [75–77] (Fig. 5a–c and Additional file 1: Figure S5E-F). Treatment with anti-inflammatory reagents *after* chemotherapy is not very effective as the downstream survival effectors have already been induced (Additional file 1: Figure S5F); thus, targeting their upstream cytokine regulators would not be effective at this later time (Figs. 4f, g and 5f–h and Additional file 1: Figure S5C). Therefore, treatment with reagents that block these resistance pathways *prior to* (and continued with) or along with chemotherapy, enables the most effective reduction of resistance, as they prevent further enrichment of such resistant cells by blocking induction of pro-survival signaling.

Increasing AraC, a nucleotide analog that inhibits replication [17], would activate DDR and downstream p38 MAPK signaling [63–65] and should lead to more cells expressing this inflammatory pathway that enables resistance. Consistently, increased AraC treatment leads to more cells in the inflammatory phase that can be targeted by LY to curb resistance (Fig. 4k). Non-cancerous cells are not affected by these inhibitors (Fig. 4j and Additional file 1: Figure S5I). These data suggest that certain chemotherapies and stresses like serum starvation induce stress signaling (Fig. 4a–c and Additional file 1: Figure S4A-B) and enrich for resistant G0 cells—in addition to pre-existing subpopulations with genomic instability that trigger DDR and stress [63–65]. Importantly, this resistance mechanism can be blocked, not only in different AML cell lines (Figs. 4j, 5g, and 7b) but also in vivo (Fig. 8c–h) and in multiple patient-derived primary AML—without affecting normal cells (Fig. 8a and Additional file 1: Figure S5I)—supporting their potential applicability as a therapeutic against chemoresistance in AML.

We find key signaling pathways induced by AraCS and SS treatments, which alter post-transcriptional and translational gene expression to enable resistance. These include: 1. DNA damage ATM [63–65] and stress activated p38 MAPK that in turn promotes MK2 [67, 68], to post-transcriptionally upregulate ARE-bearing mRNAs [59, 61, 62]. The expressed mRNAs include ARE-bearing pro-inflammatory cytokine TNFα [73, 74] & its receptors that activates downstream anti-apoptosis signals (Fig. 4a–d and Additional file 1: Figure S4A-B, Fig. 5a–c and Additional file 1: Figure S5E-F) [75–77], and ARE-bearing signaling regulator DUSP1 [83, 84] that blocks JNK-mediated apoptosis (Fig. 6), to promote resistance. 2. UPR and PKR stress signaling are induced downstream of p38 MAPK [88] and DNA damage [89, 90], and inhibit canonical translation via PERK and PKR phosphorylation of eIF2α (Fig. 2a–c). This enables non-canonical translation of specific mRNAs when this rate-limiting step of canonical translation initiation is reduced [89, 90]. 3. In addition, DNA damage signaling can also cause suppression of the other rate-limiting step of canonical translation initiation, by dephosphorylation of 4EBP [63, 64]. Consistently, 4EBP dephosphorylation is observed here, although more moderately in AraCS cells compared to SS cells (Fig. 2d), and moderately in both conditions for S6, a second canonical translation regulator (Additional file 1: Figure S1I). These changes in post-transcriptional and translational mechanisms allow specific translation of pro-inflammatory cytokines [14] (Fig. 3a–c) and immune modulators [46] (HLA-G, CD47, Fig. 2f–g and Additional file 1: Figure S2C) [47–49] that regulate antitumor immune response and resistance [50, 51]. While the translation inhibitor, 4EBP that is downstream of the mTOR pathway, is dephosphorylated in these conditions and can reduce canonical translation (Fig. 2d), mTOR phosphorylation was not coordinately altered (Additional file 1: Figure S1I), indicating that the mTOR pathway shows differences at different levels in SS and AraCS cells. This is likely due to feedback regulation from S6K and other downstream kinases that can affect mTOR, as observed in other systems [26, 36–41], while 4EBP is also known to be regulated by other kinases, independent of mTOR [42–45].

Blocking the p38 MAPKα/β pathway with LY [68, 69] (Fig. 4c), in combination with the anti-inflammatory PFD [68, 71, 72] that precludes downstream TNFα expression [71, 72] (Fig. 5e)—prior to (and continued with) AraC chemotherapy—lead to effective loss of chemoresistance in multiple AML cell lines (Fig. 7b), in tumors in vivo in AML mouse models (Fig. 8c–h), and in patient samples (Fig. 8a and Additional file 1: Figure S5I), validating their ability to reduce resistance and tumors in vitro and in vivo. LY destabilizes TNFα mRNA by TTP dephosphorylation (Fig. 3g and 4c) [59], while PFD suppresses TNFα selectively at the translation level [72] (Fig. 5e and Additional file 1: Figure S5B) and thus enables PLA combination therapy to more effectively curb resistance than the individual drugs (Figs. 7b and 8a, b). Apart from its effect on TNFα translation, PFD blocks inflammation regulator p38 MAPKγ [91, 92] that can be increased upon p38MAPKα/β inhibition, preventing feedback reactivation of inflammation, and enabling PLA combination therapy to remain more efficacious than the individual drugs. Therefore, the combination of PFD and LY suppresses the inflammatory and stress response more effectively in vitro and in vivo (Figs. 7 and 8). Upon inhibition of p38 MAPK, in addition to reduction of TNFα and its downstream antiapoptotic signals, we find the ARE-bearing DUSP1 is reduced, leading to activation [83, 84] of the JNK pathway [93] to promote apoptosis (Fig. 6e, f). These data indicate that blocking pro-inflammatory effectors—that are induced by chemotherapy mediated DNA damage and stress signaling—leads to increased chemosensitivity and decreased resistant cell survival.

Our findings revealed that these pro-inflammatory and signaling genes upregulated in G0, have AREs and other UTR sequences that regulate mRNA levels and translation (Fig. 3a–c and Additional file 1: Figure S3A-B). The ATM-p38 MAPK-MK2 axis stabilizes these ARE-bearing pro-inflammatory cytokine and signaling mRNAs by phosphorylating ARE-binding mRNA decay factor, TTP, to prevent its mRNA decay activity on pro-inflammatory cytokine TNFα (Figs. 3e–h and 4c, d, h, i) and signaling regulator, DUSP1 (Fig. 6a–e and Additional file 1: Figure S5G). There may be additional contributors to TTP besides MK2: p38 MAPK also directly phosphorylates TTP in macrophages [94, 95] while MEKK1 can act as a TTP kinase with TNF receptor-associated factor 2 (TRAF2) [96]. These could be involved in prolonged serum starvation, as phosphorylation of p38 MAPK is reduced after 1 day of serum starvation. In addition, protein phosphatase (PP2A) is known to dephosphorylate TTP. If PP2A activity is reduced in prolonged serum starvation, TTP can remain phosphorylated.

In support of the critical role of TTP regulation in chemoresistance, overexpression of TTP-AA—that cannot be phosphorylated and is a dominant active form that restores ARE mRNA decay [59–61]—decreases TNFα and DUSP1 expression (Figs. 3f–h and 6a–d), and thereby reduces chemoresistance (Figs. 4h, i and 6e, f). This is consistent with previous studies on AREs in cancers [14, 30, 59, 97–100]. These data suggest that phospho-TTP level or TTP activity is an important regulator of inflammatory response-mediated chemoresistance, which can be harnessed as a marker and target against AML resistance. Consistently, published in vivo leukemia resistance models show increased expression of TTP and ARE-bearing genes [15, 101], similar to our studies (Fig. 3a–e). Our studies on TTP and ARE regulated immune and signaling modulators that promote chemoresistance, are consistent with recent findings of TTP regulation of PDL1 to mediate immuno-resistance in solid tumors [102]. Importantly, inhibition of these pathways curtails chemoresistance and tumor survival in vivo in primary AML patients and tumor models (Fig. 8 and Additional file 1: Figure S5I-J). Together, these pathways that are upregulated in resistant cells (Figs. 4a, 5a, and 6a) via chemotherapy and stress-induced signaling—decrease canonical translation and permit non-canonical post-transcriptional regulation of specific genes (Additional file 1: Figure S6)—to promote chemosurvival of G0 cancer cells.

## Conclusions

Our studies reveal that G0 leukemic cells are chemoresistant, indicating their clinical importance in cancer persistence. We find a specific proteomic and translation profile that is induced commonly between G0 cells and chemosurviving leukemic cells. We uncovered critical genes that are upregulated post-transcriptionally for cell survival in these conditions by key, survival signaling pathways. These studies reveal the significance of post-transcriptional regulation of pro-inflammatory genes and signaling modulators in chemoresistance in leukemia. Our data enabled the development of a new combination therapy to effectively reduce resistance in cancer cell lines, in tumors in vivo, and in patient tumor samples.

## Methods

### Overview, aim, design, and setting

Therapeutic targeting of minimal residual disease or chemoresistant, leukemic stem cells in leukemias, particularly acute myeloid leukemia, has been ineffective thus far and refractory leukemia is fatal. The mechanisms of translation and post-transcriptional control, and the critical translation profile that control the ultimate, specific protein profile, and thereby survival of such clinically resistant cells, are largely undiscovered. Therefore, we globally analyzed gene expression at every level—RNA levels, translatome, and proteome—in chemotherapy-surviving G0 cancer cells in acute monocytic leukemia and other cancers, the specialized post-transcriptional and translational mechanistic changes, and their key signaling regulatory pathways, as well as developed a new, resistance-gene expression targeting therapy to understand and reduce chemoresistance.

Detailed description of characteristics, materials used, methods, and statistical analyses including cell culture, patient samples, tumor models, profiling, plasmids, cell viability assays, flow cytometry, protein analysis, drugs, and motif analysis is described in detail below.

### Cell culture

THP1 cells were cultured in Roswell Park Memorial Institute (RPMI)1640 media supplemented with 10% fetal bovine serum (FBS), 2 mM l-glutamine, 100 μg/mL streptomycin, and 100 U/ml penicillin at 37 °C in 5% CO_2_. SS THP1 cells were prepared by washing with PBS followed by serum starvation at a density of 2 × 10^5^ cells/mL and AraCS cells, by treatment with 5 μM AraC for 3 days or 9 days. MCF7, HFF, HEPG2, and U2OS cells were cultured in Dulbecco’s modified Eagle’s medium (DMEM) media with 10% FBS, 2 mM l-glutamine, 100 μg/mL streptomycin, and 100 U/ml penicillin, as done previously [13, 14]. MCF7 cells were serum-starved or treated with 150 μM doxorubicin. THP1 (TIB-202), MV4:11 (CRL-9591), K562 (CCL243), HFF (SCRC-1041), MCF7 (HTB-22), U2OS (HTB-96), and HEPG2 (HB-8065) were obtained from ATCC. MOLM13 (ACC554), NOMO1 (ACC542) and MONOMAC6 (ACC124) were obtained from DSMZ. Cell lines kindly provided by David Scadden [87] and MOLM13-GFP-Luc by Monica Guzman [103]. As previously described [60, 61], we used bone marrow-derived macrophages (BMDMs) transduced with plasmids coding for doxycycline-inducible GFP-TTP, GFP-TTP-AA, or GFP. Cell lines were tested for Mycoplasma (Promega) and authenticated by the ATCC Cell Authentication Testing Service [87].

### Primary AML patient samples and human monocytes

All human samples (de-identified) were handled in accordance with IRB protocols to SV (2015P000998/MGH), approved by the Partners Human Research Committee Institutional Review Board (IRB)/MGH IRB, to DAS and to TG (DF/HCC 13-583), approved by DF/HCC Office for Human Research Studies. AML samples used in this study were obtained by DAS including the following: MGH15—bone marrow 60% blasts, karyotype 46, XX, t(9;11)(p22;q23)[20/20]; MGH22—peripheral blood, 60% blasts, karyotype 46,XX,t(3;21)(q26;q22),t(9;22)(q34;q11.2) [18]/46,XX [2]; and MGH25—bone marrow, 90% blasts, karyotype 46,XX [20] and by JL-S and TG including bone marrow samples: EQ1899, CI2095, PO2038, LA2053, NC1866, GO1122, CM2164, MV2192, VD2160, XD2101, VL2317, and OA2500. Bone marrow or peripheral blood mononuclear cells were isolated from de novo AML patients by ficoll density gradient centrifugation and cryopreserved with DMSO in a liquid nitrogen tank. Thawed cells were maintained in RPMI media with 10% FBS for several days before drug treatment and analyses. Human CD34+ monocytes (2 M-101) were obtained from Lonza. Primary cells from MLL-AF9, HoxA9/Meis1 mouse models were provided by DS [3]. Mouse primary cells were maintained in RPMI media with 10% FBS, 2 mM l-glutamine, 100 μg/mL streptomycin, 100 U/ml penicillin, 5 ng/ml murine IL-3, and 25 ng/ml murine stem cell factor (SCF).

### In vivo AML mouse models

AML mouse models have been shown to predict therapy response accurately [104]. C57BL/6 and NSG were obtained from MGH Cox-7 Gnotobiotic animal facility of the AAALAC-accredited Center for Comparative Medicine and Services at MGH. C57BL/6 or NSG mice were injected intravenously or subcutaneously with MOLM13 cells expressing luciferase or intravenously with HoxA9/Meis1 or MLL-AF9 [103, 105]. IVIS imaging system (Perkin Elmer) were used to confirm engraftment of AML cells. Mice were intraperitoneally injected with 200 μl of luciferase substrate D-Luciferin (15 mg/ml) and anesthetized. Images were taken 5 or 10 min after D-Luciferin injection. After confirmation of engraftment by IVIS imaging, mice were randomly assigned to two groups and treated with pirfenidone (100 mg/kg, intraperitoneally), LY2228820 (20 mg/kg, intraperitoneally), AraC (30 mg/kg, intraperitoneally), or saline according at indicated combinations and dosages. Tumor volumes were measured by IVIS imaging at indicated time points.

### Polysome profiling with microarray

Sucrose was dissolved in lysis buffer containing 100 mM KCl, 5 mM MgCl_2_, 100 μg/ml cycloheximide, 2 mM DTT, and 10 mM Tris-HCl (pH 7.4). Sucrose gradients from 15 to 50% were prepared in ultracentrifuge tubes (Beckman) as previously described [13, 106–108]. Cells were treated with 100 μg/mL cycloheximide at 37 °C for 5 min before collecting them. Harvested cell were rinsed with ice-cold PBS having 100 μg/mL cycloheximide and then were resuspended in lysis buffer with 1% Triton X-100 and 40 U/mL murine (New England Biolabs) for 20 min. After centrifugation of cell lysates at 12,000×*g* for 20 min, supernatants were loaded onto sucrose gradients followed by ultracentrifugation (Beckman Coulter Optima L90) at 34,000 × rpm at 4 °C for 2 h in the SW40 rotor. Samples were separated by density gradient fractionation system (Teledyne Isco). RNAs were purified by using TRIzol (Invitrogen) from heavy polysome fractions and whole cell lysates. The synthesized cDNA probes from WT Expression Kit (Ambion) were hybridized to Gene Chip Human Transcriptome Array 2.0 (Affymetrix) and analyzed by the Partners Healthcare Center for Personalized Genetic Medicine Microarray and BUMC facilities. Gene ontology analysis for differentially expressed translatome or proteome was conducted by DAVID 6.7 tools [109, 110]. Molecular signatures enriched in AraCS or SS were identified by Gene Set Enrichment Analysis (GSEA) [111].

### Plasmids

TRIPZ plasmids expressing shRNA against human TNFα (V2THS_111606), and miR30a primiR sequences used as control (RHS4750), were obtained from Open Biosystems and MGH cancer center, respectively. Stable cell lines were constructed as described by Open Biosystems. The stable cells expressing shRNA against TNFα were induced with 1 μg/mL doxycycline at indicated time points to knockdown TNFα. Luciferase reporters to test ARE expression were previously described [106]. Cells were treated with 10 ng/ml recombinant TNFα (R&D Systems) to activate the NFκB pathway. Myc-tagged TTP-AA [112, 113] was a gift from Nancy Kedersha and Shawn Lyons from Paul Anderson’s lab.

### MTS assay

MTS assay, a colorimetric quantification of viable cells, was conducted as described by the manufacturer, Promega. A volume of 100 μl cells was placed in a 96-well plate after drug treatment. A volume of 20 μl MTS reagent (CellTiter 96® Aqueous Non-Radioactive Cell Proliferation Assay) was added to each well followed by incubation at 37 °C for 1 h. Absorbance was measured at 490 nm by using a microplate reader.

### Caspase 3/7 assay

After drug treatment, cell death was measured by using caspase-glo® 3/7 assay kit (Promega) according to the protocol provided by the manufacturer. The equal volume of caspase-glo reagent was added to cells, and samples were gently mixed with pipetting. The plates were incubated at room temperature in the dark for 2 h. The luminescence of each sample was measured in a luminometer (Turner BioSystems).

### Flow cytometry and cell cycle analysis

Cell proliferation was determined by flow cytometry of cells labeled with propidium iodide and bromodeoxyuridine (BrdU). The cells were incubated with 10 μM BrdU for 90 min at 37 °C in 5% CO_2_ before harvesting. Collected cells were fixed in ice-cold 70% ethanol overnight. Cells were washed in PBS and treated with 2 M HCl for 30 min. Cells were incubated for 1 h with anti-BrdU antibody conjugated to FITC (eBioscience) in the dark, washed, and stained with propidium iodide. Samples were filtered through a nylon mesh filter and cell cycle analysis performed on the flow cytometry [114].

### Western blot analysis

Cells were collected and resuspended in lysis buffer containing 40 mM Tris-HCl (pH 7.4), 6 mM MgCl_2_, 150 mM NaCl, 0.1% NP-40, 1 mM DTT, and protease inhibitors (Roche). Samples containing 80 μg of protein were loaded onto 10% or 12% SDS-PAGE (Bio-Rad), transferred to Nitrocellulose membranes and processed for immunoblotting. Antibodies against p27 (#06–445) and tubulin (#05–829) were obtained from Millipore. Antibodies against HES1 (#sc-25,392), eIF2α (#sc-11,386), and GFP (#sc-9996) were from Santa Cruz. Antibodies against phospho-ATM (#ab81292), phospho-PKR (#ab32036), DUSP1 (#ab138265), and phospho-IRE1 (#ab124945) were from Abcam. Antibody against RPS6 (#66886-1-Ig) was from Proteintech. Antibody against phospho-PERK (#649401) was from Biolegend. Antibodies against phospho-mTOR (Ser2448, #2971), phospho-mTOR (Ser2481, #2974), mTOR (#2983), phospho-4E-BP1 (Thr37/46, #2855), phospho-4E-BP1 (Ser65, #9451), phospho-S6 ribosomal protein (Ser235/236, #2211),TNFα (#3707), phospho-p38 MAPK (#4511), phospho-MK2 (#3007), phospho-eIF2α (#9721), TTP (#71632), JNK (#9252), phospho-JNK (#9251), and 4EBP1 (#9452) were from Cell Signaling Technology.

### qPCR

Total RNA was isolated using TRIzol (Invitrogen) according to the manufacturer’s instructions. The cDNA was synthesized from 1 μg of RNA using M-MuLV Reverse Transcriptase (NEB) and random hexamer primer (Promega). qPCRs were run on LightCycler® 480 Instrument II (Roche) using 2X SYBR green mix (Bio-rad). The primers used in the qPCR were as follows: mouse TNF-α sense 5′-GCCTCTTCTCATTCCTGCTTG-3′, antisense 5′-CTGATGAGAGGGAGGCCATT-3′; mouse Gapdh sense 5′-CATGGCCTTCCGTGTTCCT-3′, antisense 5′-TGATGTCATCATACTTGGCAGGTT-3′; Dusp1 sense 5′-GGCCAGCTGCTGCAGTTTGAG-3′, antisense 5′-AGGTGCCCCGGTCAAGGACA-3′.

### Apoptosis analysis

Leukemic cells were treated with indicated drug combinations. Annexin V FITC/PI staining was performed with FITC Annexin V Apoptosis Detection Kit I (BD Pharmingen). Flow cytometry analysis and FlowJo software were used to quantify the percentages of apoptotic cells.

### Colony-forming assay

After treatment with indicated drug combinations, the same number of cells was plated in methylcellulose-based media with human recombinant cytokines (stem cell technology, MethoCult™ H4435). Number of colonies was quantified in each plate after 10 days.

### Mass spectrometry

Multiplex quantitative proteomics analysis was conducted, as performed previously [115], in S+-, SS-, and AraC-treated THP1 leukemic cells.

### Immunoprecipitation

Expression of GFP-tagged TTP-AA mutant was induced with 1 μg/ml doxycycline prior to 1 μM AraC treatment in TTP-deficient BMDM cells. The cells were cross-linked with UV 254 nm. Cells were lysed in lysis buffer (20 mM Tris, pH 7.5, 150 mM NaCl, 1 mM EDTA, 1 mM EGTA, 1% Triton X-100, 2.5 mM sodium pyrophosphate, 1 mM β-glycerophosphate, 1 mM Na3VO4, protease inhibitor, RNase inhibitor). Cell lysates were incubated overnight at 4 °C with either IgG control or GFP antibody. Protein G agarose (Santa Cruz) was used to pull down antibody bound RNA-protein complexes.

### Inhibitors

Pirfenidone (10 to 300 μg/ml [71, 116–118]) was obtained from Chemietek. AraC (1 to 10 μM [119, 120]), LY2228820 (0.03 to 2 μM [68, 69, 121, 122]), BIRB796 (BIRB, 5 μM [70, 123–126]), and JNK-IN-8 (1 μM [127] were from Selleckchem. KU55933 (10 μM [128], tested but was toxic for the cells tested), BAY 11-7082 (10 μM [78]), and D-luciferin were from Cayman Chemical and doxorubicin (10 to 500 nM [129]) was from Tocris Bioscience.

### Motif, AREs, RNA-binding proteins, ribosome occupancy, and GSEA analysis

The Multiple Em for Motif Elicitation (MEME) software was used to search for cis-elements enriched in 5′ UTR of translationally regulated genes [130]. Human 5′ UTR sequences were retrieved from UCSC table browser [131]. In a discriminative mode, 5′ UTR sequences of translationally up- or downregulated genes were used as the primary sequences and 5′ UTR sequences of translationally unchanged genes, the control sequences. Motifs were found in the given strand with 6–30 nt motif width. We compared polysome-associated mRNAs with their total RNA levels in serum-starved and AraCS cells to generate the change in ribosome occupancy (RO) [132–134]—which is the ratio of the level of mRNA that is associated with heavy polysomes compared to the total mRNA level of each gene (Fig. 2f, heat map, Additional file 2: Table S1). ARE Score algorithm [135] was used to assess scores of AU-rich elements quantitatively. The list of RNA-binding protein genes were obtained from RBPDB database [136]. Gene Set Enrichment Analysis (GSEA) was performed using all 50 gene sets of the Hallmarks, and gene sets from KEGG, reactome, and GO pathways from the Molecular Signatures Database (MSigDB) [111, 137] with our transcriptome, translatome, and proteome datasets.

### Statistical tests and differential gene expression analyses

All experiments were performed with at least three replicates except for experiments with AML patients. Sample sizes were estimated on the basis of availability and previous experiments [13, 14]. No samples were excluded from analyses. Statistical methods were not used to pre-determine sample size. Two-tailed *t*-test and Wilcoxon rank sum test were performed for statistical tests. SEM (standard error of mean) values are shown as error bars in all figures. Means were used as center values in box plots. *p* values less than 0.05 were indicated with an asterisk. *E*-values were used for the statistical significance in the motif analysis. Affymetrix microarray data were normalized and summarized using the RMA method implemented in the affy R package [138]. Genes with small variation or a consistently low signal across samples were filtered by the varFilter function in the genefilter package. A robust linear regression model was then used to fit to the probe intensities using the lmFit function, followed by the detection of differentially expressed genes using the eBayes function in the limma R package [139]. Differentially expressed genes were identified using *p* < 0.05 and log2 fold change of ± 0.585 (1.5-fold change). The statistical significance of overlaps between two different groups of genes was assessed using hypergeometric probability test (http://nemates.org/MA/progs/overlap_stats.cgi), (Additional file 1: Figure S3G), with the total number of proteins being 26,809, based on our arrays.

## Supplementary information

Additional file 1. Figure S1. Related to main Fig. 1. Figure S2. Related to main Fig. 2. Figure S3. Related to main Fig. 3. Figure S4. Related to main Figs. 4, 5 and 6. Figure S5. Related to main Figs. 5, 6 and 7. Figure S6. Related to main Figs. 1, 2, 3, 4, 5, 6, 7 and 8.

Additional file 2. Table S1. Related to main Figs. 1, 2, 3, 4, 5, 6, 7 and 8. RNAs up-regulated at the translatome level and their RO changes in G0 cells induced by SS or AraC.

Additional file 3. Table S2. Related to main Figs. 1, 2, 3, 4, 5, 6, 7 and 8. RNAs bearing AU-rich elements (AREs) and up-regulated at the translatome level in G0 cells induced by SS or AraC.

Additional file 4. Uncropped Western blots.

Additional file 5. Review history.

## Data Availability

Raw datasets are available on the public repository, GEO, with series accession numbers GSE141075 and GSE141329 which are included in Super Series GSE141332 [140]. All materials will be made available publicly on publication and on request.
