# Supplementary material for: A post-transcriptional program of chemoresistance by AU-rich elements and TTP in quiescent leukemic cells
Source: Genome Biol. 2020 Feb 10;21:33. doi: 10.1186/s13059-020-1936-4 (PMC7011231; doi:10.1186/s13059-020-1936-4)
Supplement: Supplementary file 1 — Figure S1. Related to main Fig. 1. Figure S2. Related to main Fig. 2. Figure S3. Related to main Fig. 3. Figure S4. Related to main Figs. 4, 5 and 6. Figure S5. Related to main Figs. 5, 6 and 7. Figure S6. Related to main Figs. 1, 2, 3, 4, 5, 6, 7 and 8. [file 13059_2020_1936_MOESM1_ESM.pdf]

Additional File 1

A post-transcriptional program of chemoresistance by AU-rich elements and TTP in quiescent leukemic cells

List of Additional Files

1. Additional File 1:

Supplemental Figures S1-S6 and legends

Supplemental Tables legends (for Additional File 2: Table S1 and Additional File 3: Table S2)

References

2. Additional File 2: Table S1

3. Additional File 3: Table S2

4. Additional File 4: uncropped Western blots for Main and Supplemental Figures

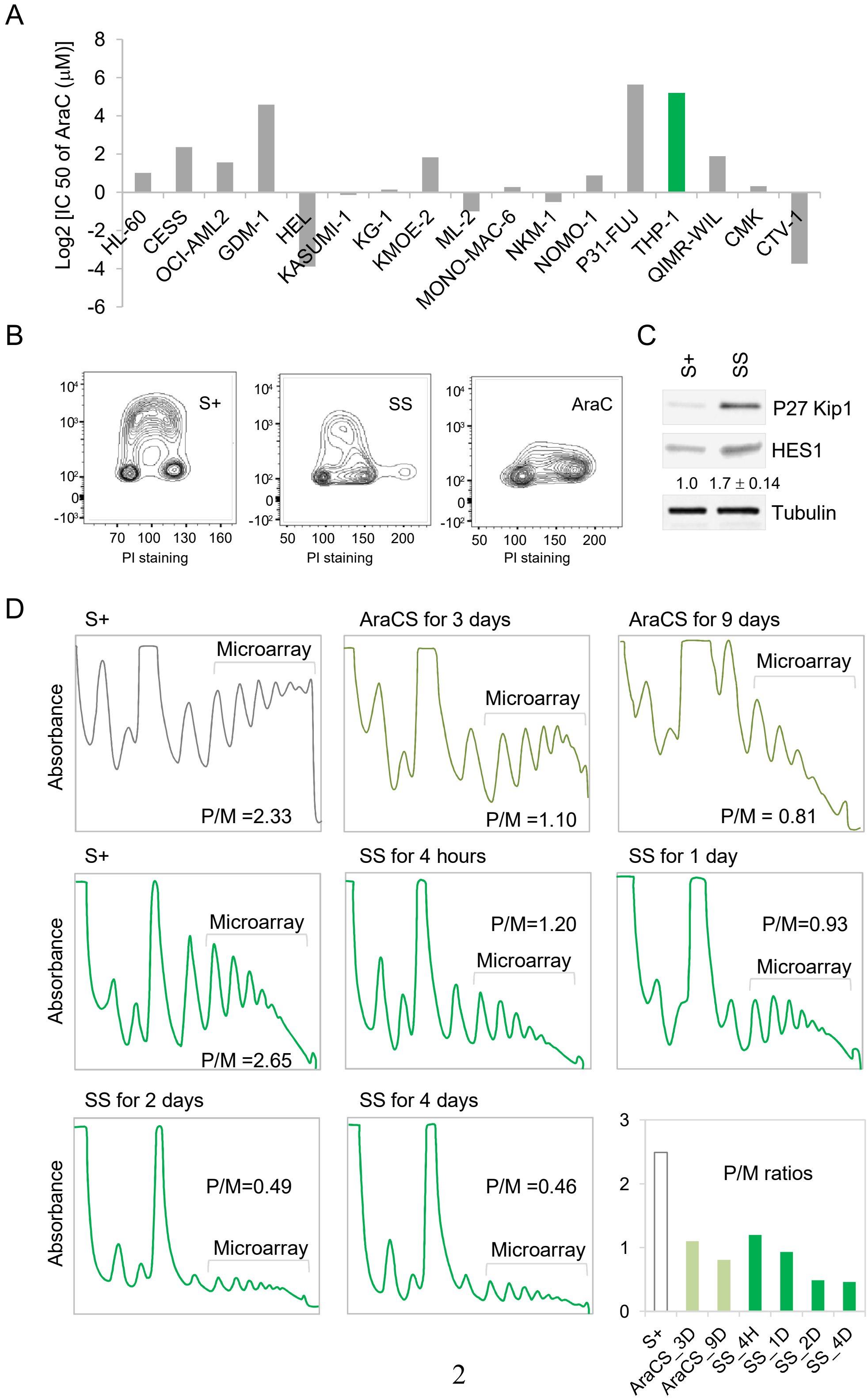
A
Log2 [IC 50 of AraC (μM)]
8
6
4
2
0
-2
-4
-6
HL-60
CESS
OCI-AML2
GDM-1
HEL
KASUMI-1
KG-1
KMOE-2
ML-2
MONO-MAC-6
NKM-1
NOMO-1
P31-FUJ
THP-1
QIMR-WIL
CMK
CTV-1
B
S+
SS
AraC
PI staining
C
S+
SS
P27 Kip1
HES1
1.0
1.7 ± 0.14
Tubulin
D
S+
AraCS for 3 days
AraCS for 9 days
Microarray
Absorbance
P/M =2.33
P/M =1.10
P/M = 0.81
SS for 4 hours
SS for 1 day
P/M =2.65
P/M=1.20
P/M=0.93
SS for 2 days
SS for 4 days
P/M=0.49
P/M =0.46
P/M ratios
3
2
1
0
S+
AraCS_3D
AraCS_9D
SS_4H
SS_1D
SS_2D
SS_4D

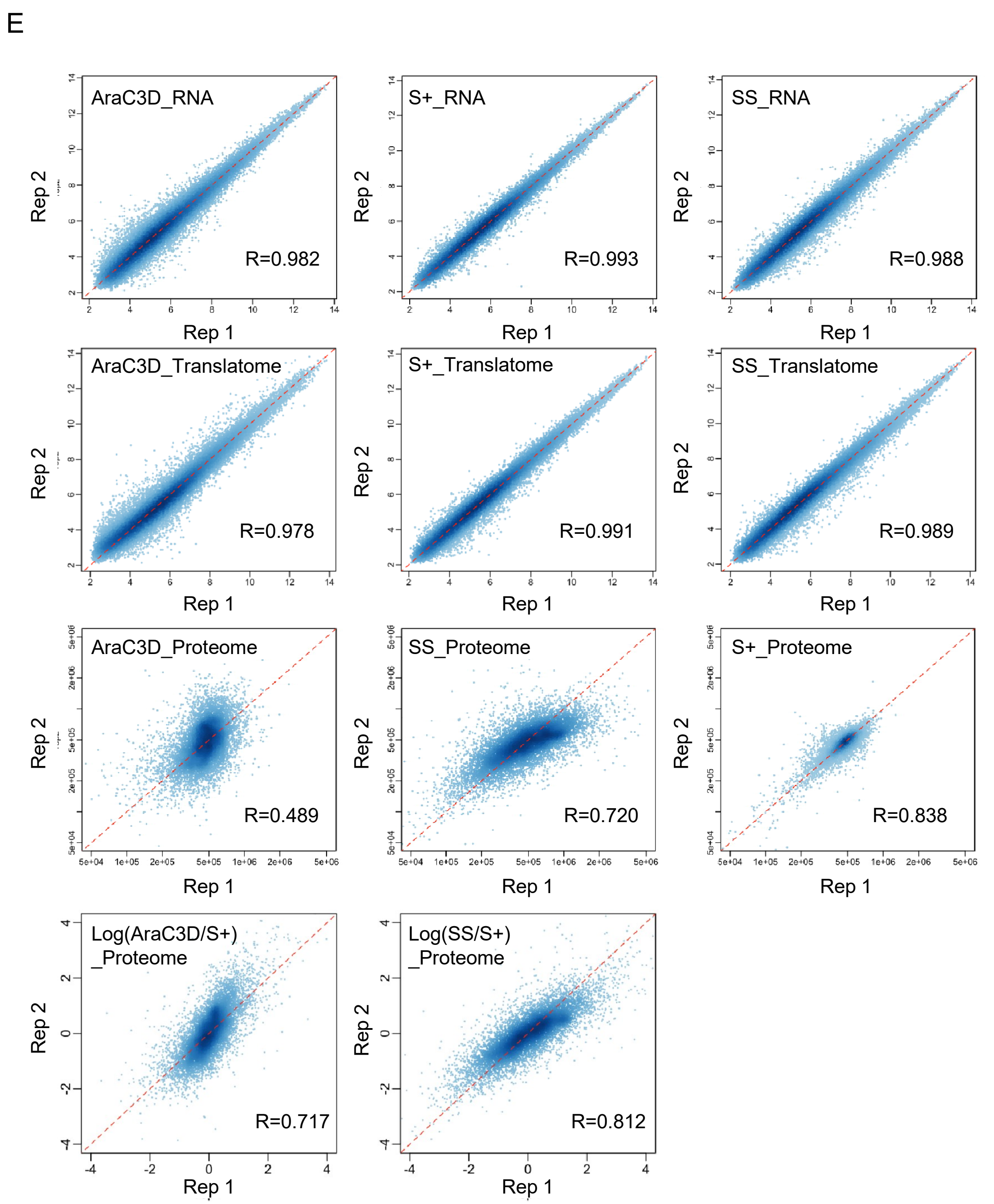
E
AraC3D_RNA
R=0.982
S+_RNA
R=0.993
SS_RNA
R=0.988
AraC3D_Translatome
R=0.978
S+_Translatome
R=0.991
SS_Translatome
R=0.989
AraC3D_Proteome
R=0.489
SS_Proteome
R=0.720
S+_Proteome
R=0.838
Log(AraC3D/S+)
_Proteome
R=0.717
Log(SS/S+)
_Proteome
R=0.812
Rep 1
Rep 2

F

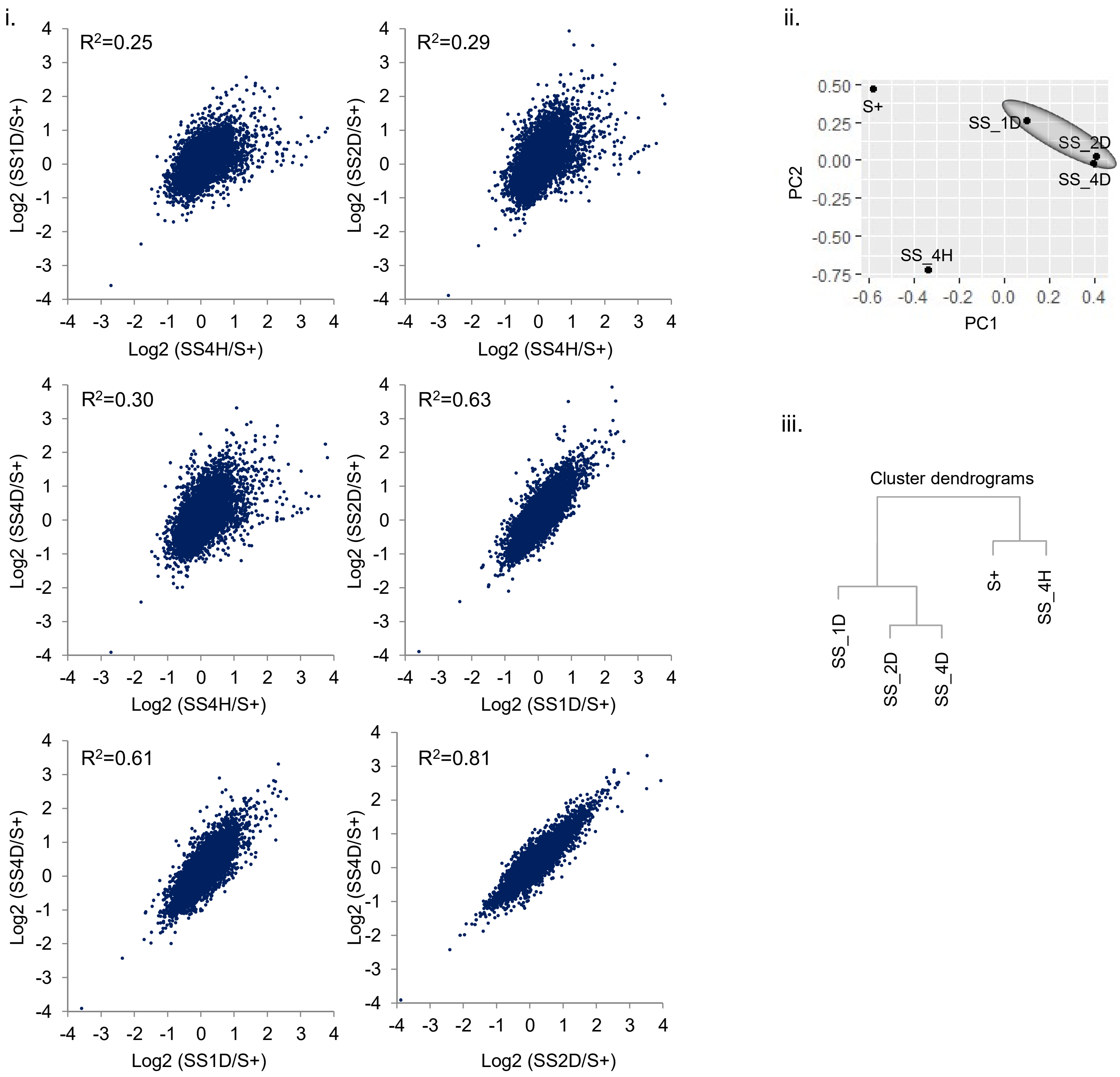
i.
R^2=0.25
Log2 (SS1D/S+)
Log2 (SS4H/S+)
R^2=0.29
Log2 (SS2D/S+)
Log2 (SS4H/S+)
R^2=0.30
Log2 (SS4D/S+)
Log2 (SS4H/S+)
R^2=0.63
Log2 (SS2D/S+)
Log2 (SS1D/S+)
R^2=0.61
Log2 (SS4D/S+)
Log2 (SS1D/S+)
R^2=0.81
Log2 (SS4D/S+)
Log2 (SS2D/S+)
ii.
S+
SS_1D
SS_2D
SS_4D
SS_4H
PC1
PC2
iii.
Cluster dendrograms
SS_1D
SS_2D
SS_4D
S+
SS_4H

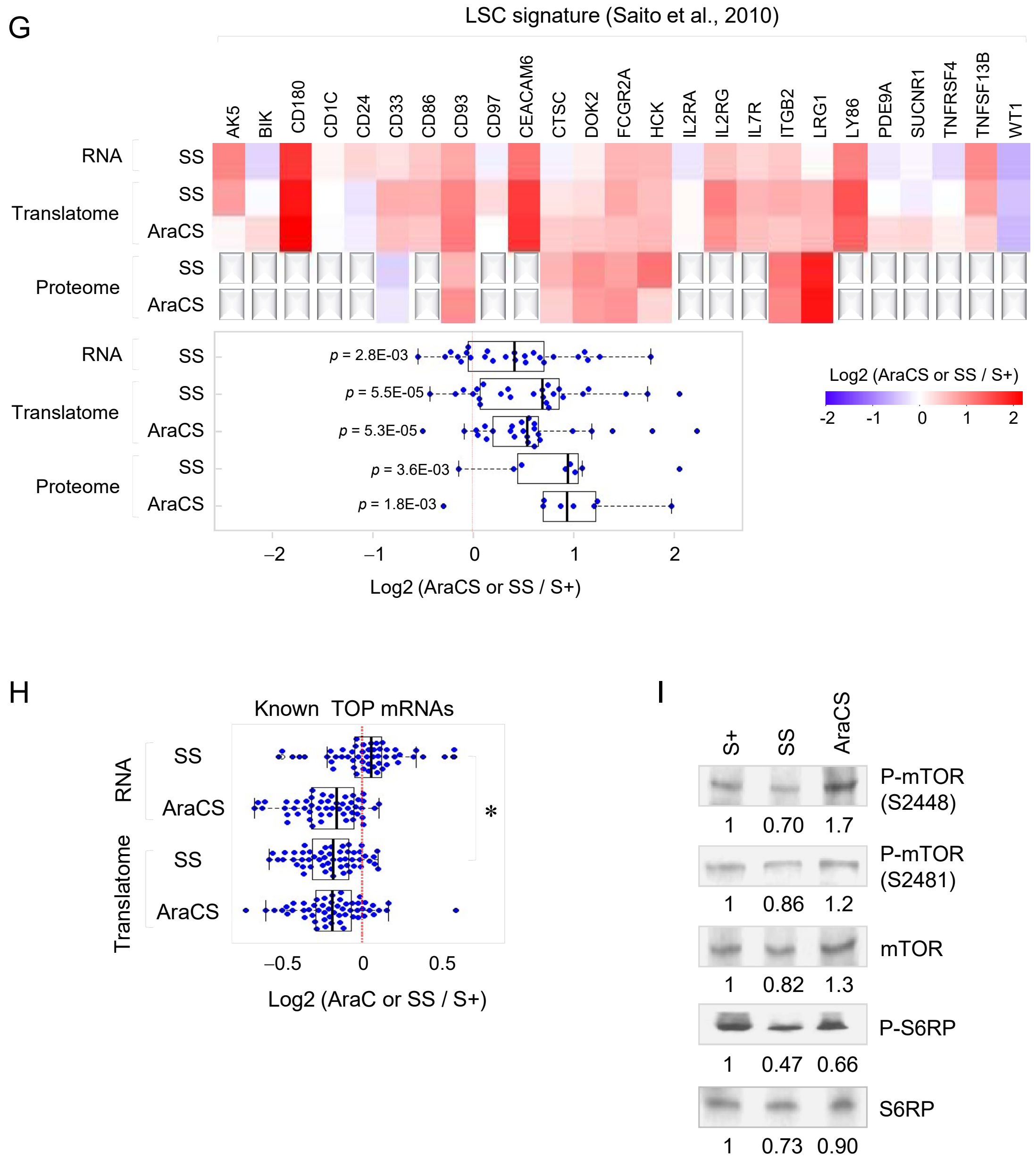
G
LSC signature (Saito et al., 2010)
AK5
BIK
CD180
CD1C
CD24
CD33
CD86
CD93
CD97
CEACAM6
CTSC
DOK2
FCGR2A
HCK
IL2RA
IL2RG
IL7R
ITGB2
LRG1
LY86
PDE9A
SUCNR1
TNFRSF4
TNFSF13B
WT1
RNA
SS
Translatome
SS
AraCS
Proteome
SS
AraCS
p = 2.8E-03
p = 5.5E-05
p = 5.3E-05
p = 3.6E-03
p = 1.8E-03
Log2 (AraCS or SS / S+)
-2 -1 0 1 2
−2 −1 0 1 2
Log2 (AraCS or SS / S+)
H
Known TOP mRNAs
RNA
Translatome
SS
AraCS
SS
AraCS
*
−0.5 0 0.5
Log2 (AraC or SS / S+)
I
S+
SS
AraCS
P-mTOR (S2448)
1 0.70 1.7
P-mTOR (S2481)
1 0.86 1.2
mTOR
1 0.82 1.3
P-S6RP
1 0.47 0.66
S6RP
1 0.73 0.90

Additional File 1: Fig. S1. Related to main figure 1. A. IC50 values of standard anti-leukemic chemotherapy, AraC, in AML cell lines (1). THP1 cell line was selected for this study as it shows strong resistance to AraC. **B.** Flow cytometric profiles of S+, SS (serum starvation for 4 days) and AraCS (5 μM AraC treatment for 3 days), using BrdU and PI staining. **C.** G0 arrest of serum-starved THP1 is assessed by Western analysis of p27 and Hes1 levels. **D.** Polysome profiles of S+, SS (serum starvation for 4 hours, 1 day, 2 days and 4 days) and AraCS (5 μM AraC treatment for 3 days and 9 days) THP1 cells. Heavy polysomes (≥3 ribosomes)-associated mRNAs were analyzed by microarray. 'P/M' indicates polysome to monosome ratios. **E.** Scatter plots and correlation coefficients between biological replicates for S+, SS and AraCS cells in the transcriptome, translatome, and proteome levels. Additionally, two plots at the bottom show correlation between two replicates from AraCS and SS normalized by the corresponding S+ replicates of the proteome profiles. **F.** Scatter plots and correlation coefficients (i), principal component analysis (PCA) analysis (ii) and unbiased hierarchical clustering (iii) of the translatomes of cells that were serum-starved for indicated times. Heatmap and boxplot of the expression of LSC gene signature (2) in AraCS and SS cells. **H.** Boxplot of the transcriptome and translatome changes of known TOP mRNAs in response to SS or AraC treatment. **I.** Western blot of mTOR phosphorylation (Serine 2481 and 2448 sites), compared to total mTOR, and of phospho-S6 and total S6, in SS and AraCS cells (3-13).

Figure S2

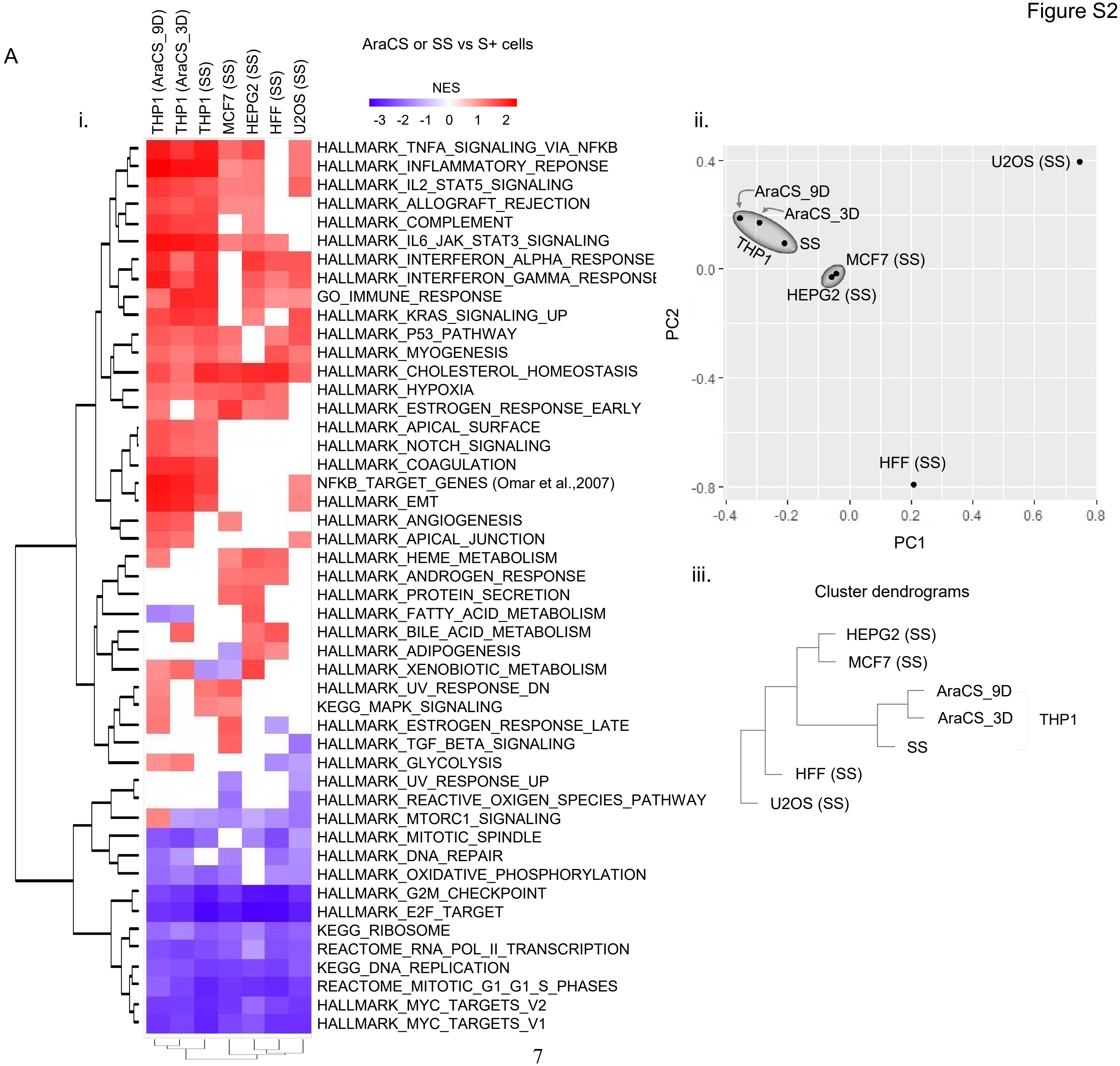
A
i.
THP1 (AraCS_9D)
THP1 (AraCS_3D)
THP1 (SS)
MCF7 (SS)
HEPG2 (SS)
HFF (SS)
U2OS (SS)
AraCS or SS vs S+ cells
NES
-3 -2 -1 0 1 2
HALLMARK_TNFA_SIGNALING_VIA_NFKB
HALLMARK_INFLAMMATORY_REPONSE
HALLMARK_IL2_STAT5_SIGNALING
HALLMARK_ALLOGRAFT_REJECTION
HALLMARK_COMPLEMENT
HALLMARK_IL6_JAK_STAT3_SIGNALING
HALLMARK_INTERFERON_ALPHA_RESPONSE
HALLMARK_INTERFERON_GAMMA_RESPONSE
GO_IMMUNE_RESPONSE
HALLMARK_KRAS_SIGNALING_UP
HALLMARK_P53_PATHWAY
HALLMARK_MYOGENESIS
HALLMARK_CHOLESTEROL_HOMEOSTASIS
HALLMARK_HYPOXIA
HALLMARK_ESTROGEN_RESPONSE_EARLY
HALLMARK_APICAL_SURFACE
HALLMARK_NOTCH_SIGNALING
HALLMARK_COAGULATION
NFKB_TARGET_GENES (Omar et al.,2007)
HALLMARK_EMT
HALLMARK_ANGIOGENESIS
HALLMARK_APICAL_JUNCTION
HALLMARK_HEME_METABOLISM
HALLMARK_ANDROGEN_RESPONSE
HALLMARK_PROTEIN_SECRETION
HALLMARK_FATTY_ACID_METABOLISM
HALLMARK_BILE_ACID_METABOLISM
HALLMARK_ADIPOGENESIS
HALLMARK_XENOBIOTIC_METABOLISM
HALLMARK_UV_RESPONSE_DN
KEGG_MAPK_SIGNALING
HALLMARK_ESTROGEN_RESPONSE_LATE
HALLMARK_TGF_BETA_SIGNALING
HALLMARK_GLYCOLYSIS
HALLMARK_UV_RESPONSE_UP
HALLMARK_REACTIVE_OXIGEN_SPECIES_PATHWAY
HALLMARK_MTORC1_SIGNALING
HALLMARK_MITOTIC_SPINDLE
HALLMARK_DNA_REPAIR
HALLMARK_OXIDATIVE_PHOSPHORYLATION
HALLMARK_G2M_CHECKPOINT
HALLMARK_E2F_TARGET
KEGG_RIBOSOME
REACTOME_RNA_POL_II_TRANSCRIPTION
KEGG_DNA_REPLICATION
REACTOME_MITOTIC_G1_G1_S_PHASES
HALLMARK_MYC_TARGETS_V2
HALLMARK_MYC_TARGETS_V1
ii.
U2OS (SS)
AraCS_9D
AraCS_3D
SS
THP1
MCF7 (SS)
HEPG2 (SS)
HFF (SS)
PC2
PC1
0.4
0.0
-0.4
-0.8
-0.4 -0.2 0.0 0.2 0.4 0.6 0.8
iii.
Cluster dendrograms
HEPG2 (SS)
MCF7 (SS)
AraCS_9D
AraCS_3D
THP1
SS
HFF (SS)
U2OS (SS)

Fig. S2

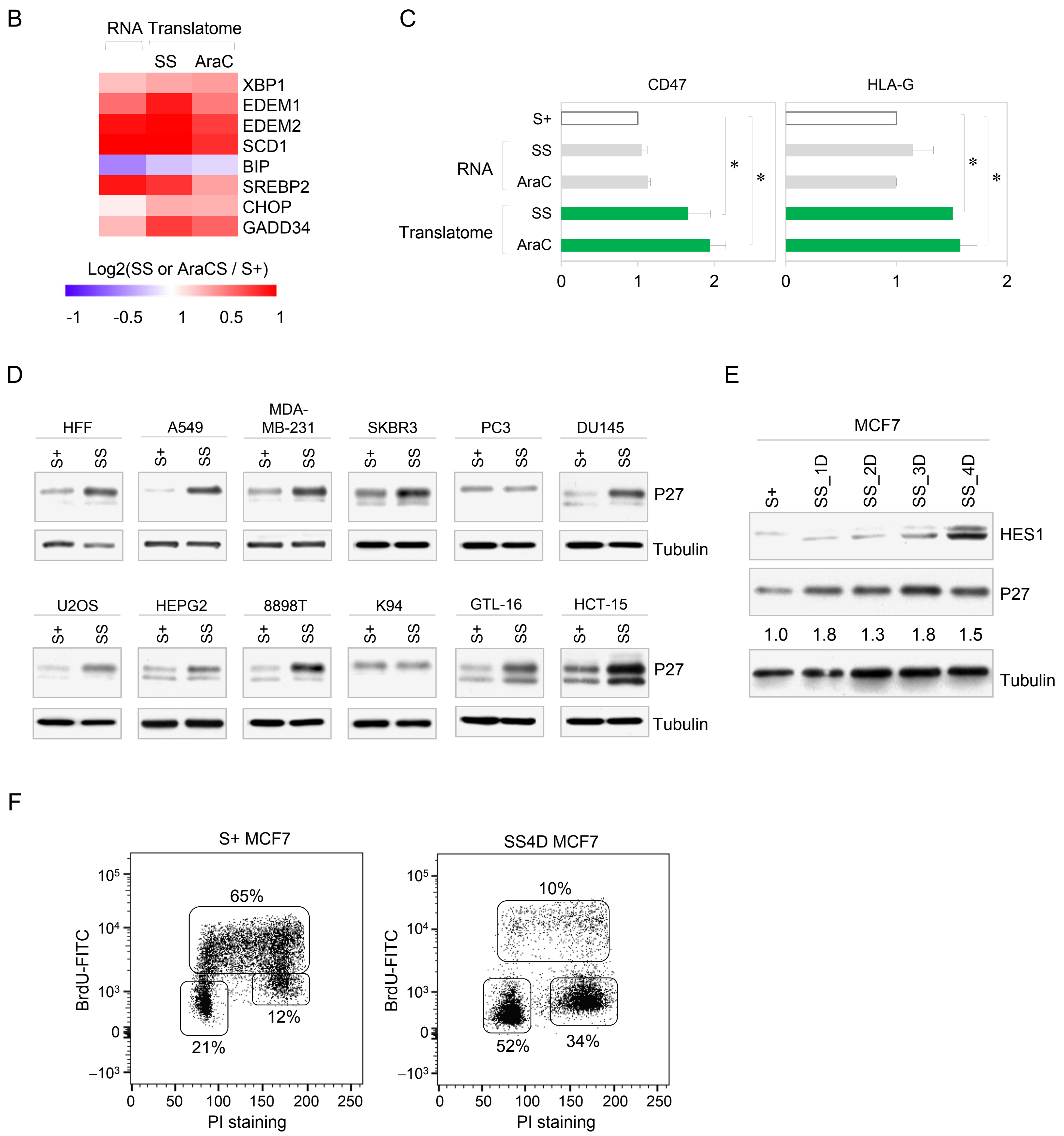
B
RNA Translatome
SS AraC
XBP1
EDEM1
EDEM2
SCD1
BIP
SREBP2
CHOP
GADD34
Log2(SS or AraCS / S+)
-1 -0.5 1 0.5 1
C
CD47
HLA-G
S+
RNA
SS
AraC
Translatome
SS
AraC
*
*
0 1 2
D
HFF
A549
MDA-MB-231
SKBR3
PC3
DU145
S+ SS
P27
Tubulin
U2OS
HEPG2
8898T
K94
GTL-16
HCT-15
E
MCF7
S+
SS_1D
SS_2D
SS_3D
SS_4D
HES1
P27
1.0 1.8 1.3 1.8 1.5
Tubulin
F
S+ MCF7
SS4D MCF7
BrdU-FITC
PI staining
65%
12%
21%
10%
52%
34%
0 50 100 150 200 250

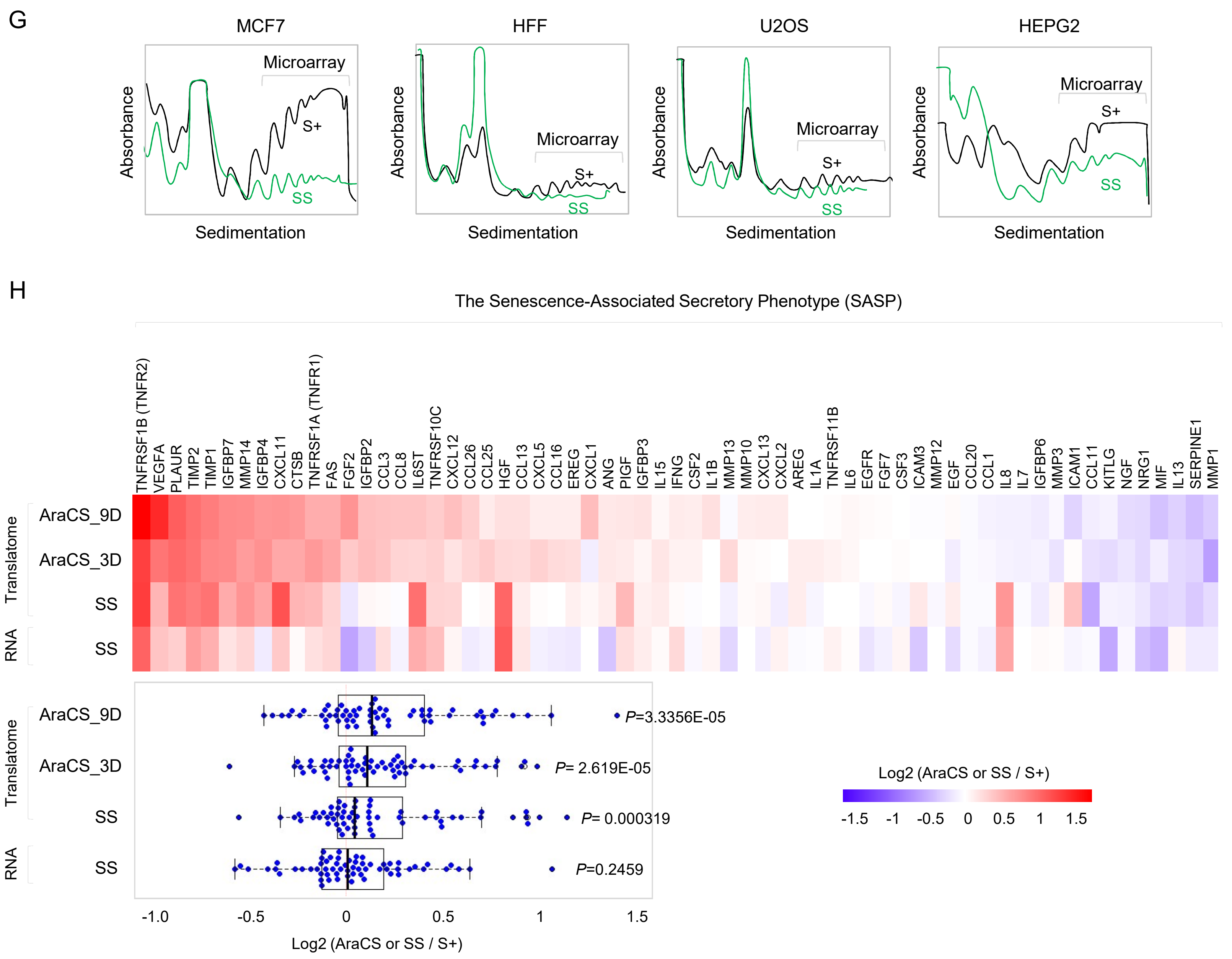
G
MCF7
HFF
U2OS
HEPG2
Microarray
S+
SS
Absorbance
Sedimentation
H
The Senescence-Associated Secretory Phenotype (SASP)
TNFRSF1B (TNFR2)
VEGFA
PLAUR
TIMP2
TIMP1
IGFBP7
MMP14
IGFBP4
CXCL11
CTSB
TNFRSF1A (TNFR1)
FAS
FGF2
IGFBP2
CCL3
CCL8
IL6ST
TNFRSF10C
CXCL12
CCL26
CCL25
HGF
CCL13
CXCL5
CCL16
EREG
CXCL1
ANG
PIGF
IGFBP3
IL15
IFNG
CSF2
IL1B
MMP13
MMP10
CXCL13
CXCL2
AREG
IL1A
TNFRSF11B
IL6
EGFR
FGF7
CSF3
ICAM3
MMP12
EGF
CCL20
CCL1
IL8
IL7
IGFBP6
MMP3
ICAM1
CCL11
KITLG
NGF
NRG1
MIF
IL13
SERPINE1
MMP1
Translatome
RNA
AraCS_9D
AraCS_3D
SS
P=3.3356E-05
P= 2.619E-05
P= 0.000319
P=0.2459
Log2 (AraCS or SS / S+)
-1.5
-1
-0.5
0
0.5
1
1.5
-1.0
-0.5
0
0.5
1
1.5

Additional File 1: Fig. S2. Related to main figure 2. A. Comparison of translatomes of G0 cells from five different cell lines. GSEA was performed using all 50 gene sets of the Hallmark, KEGG, reactome, and GO pathways from the Molecular Signatures Database (MSigDB). Heatmap of normalized enrichment score (NES) for (i) each gene set , (ii) PCA analysis , and (iii) unbiased hierarchical clustering of translatomes are shown. **B**. Heatmap of the expression of ER stress related genes in SS and AraCS cells. **C**. Bar graphs showing the expression of CD47 and HLA-G in S+, SS and AraCS cells. **D.** Western analysis of p27KIP1 (p27) in S+ and SS cells as a marker for G0/G1 arrest, shows that G0 cells are induced by serum-starvation in a number of cancer cell lines. **E.** G0 arrest of serum-starved MCF7 is assessed by Western analysis of p27 and Hes1 levels. **F**. Flow cytometric analysis of S+ and SS cells from MCF7 using BrdU and PI staining. **G.** Polysome profiles of S+ and SS cells from MCF7, U2OS, HEPG2 and non-cancerous HFF fibroblasts cell lines. Heavy polysomes ($\geq$ 3 ribosomes) were analyzed by microarray. **H**. Heatmap and boxplot of the expression of SASP signature genes in SS and AraCS THP1 cells. * $p \leq 0.05$ Data are represented as average ± SEM.

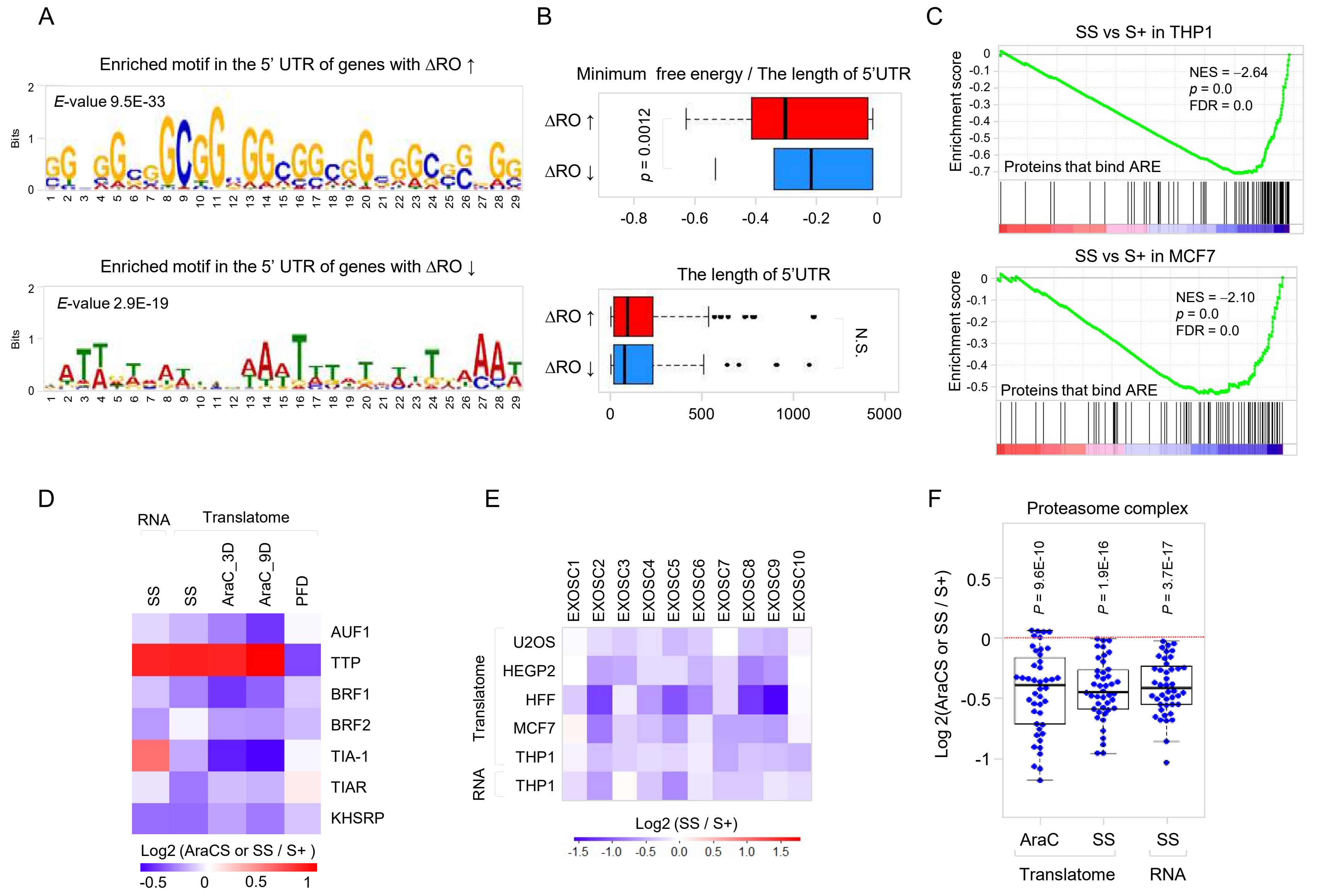
A
Enriched motif in the 5' UTR of genes with ΔRO ↑
E-value 9.5E-33
Bits
Enriched motif in the 5' UTR of genes with ΔRO ↓
E-value 2.9E-19
B
Minimum free energy / The length of 5'UTR
ΔRO ↑
ΔRO ↓
p = 0.0012
-0.8 -0.6 -0.4 -0.2 0
The length of 5'UTR
N.S.
0 500 1000 5000
C
SS vs S+ in THP1
Enrichment score
NES = –2.64
p = 0.0
FDR = 0.0
Proteins that bind ARE
SS vs S+ in MCF7
NES = –2.10
p = 0.0
FDR = 0.0
Proteins that bind ARE
D
RNA
Translatome
SS
SS
AraC_3D
AraC_9D
PFD
AUF1
TTP
BRF1
BRF2
TIA-1
TIAR
KHSRP
Log2 (AraCS or SS / S+)
-0.5 0 0.5 1
E
EXOSC1 EXOSC2 EXOSC3 EXOSC4 EXOSC5 EXOSC6 EXOSC7 EXOSC8 EXOSC9 EXOSC10
Translatome
U2OS
HEGP2
HFF
MCF7
THP1
RNA
THP1
Log2 (SS / S+)
-1.5 -1.0 -0.5 0.0 0.5 1.0 1.5
F
Proteasome complex
Log 2(AraCS or SS / S+)
P = 9.6E-10
P = 1.9E-16
P = 3.7E-17
AraC
SS
SS
Translatome
RNA

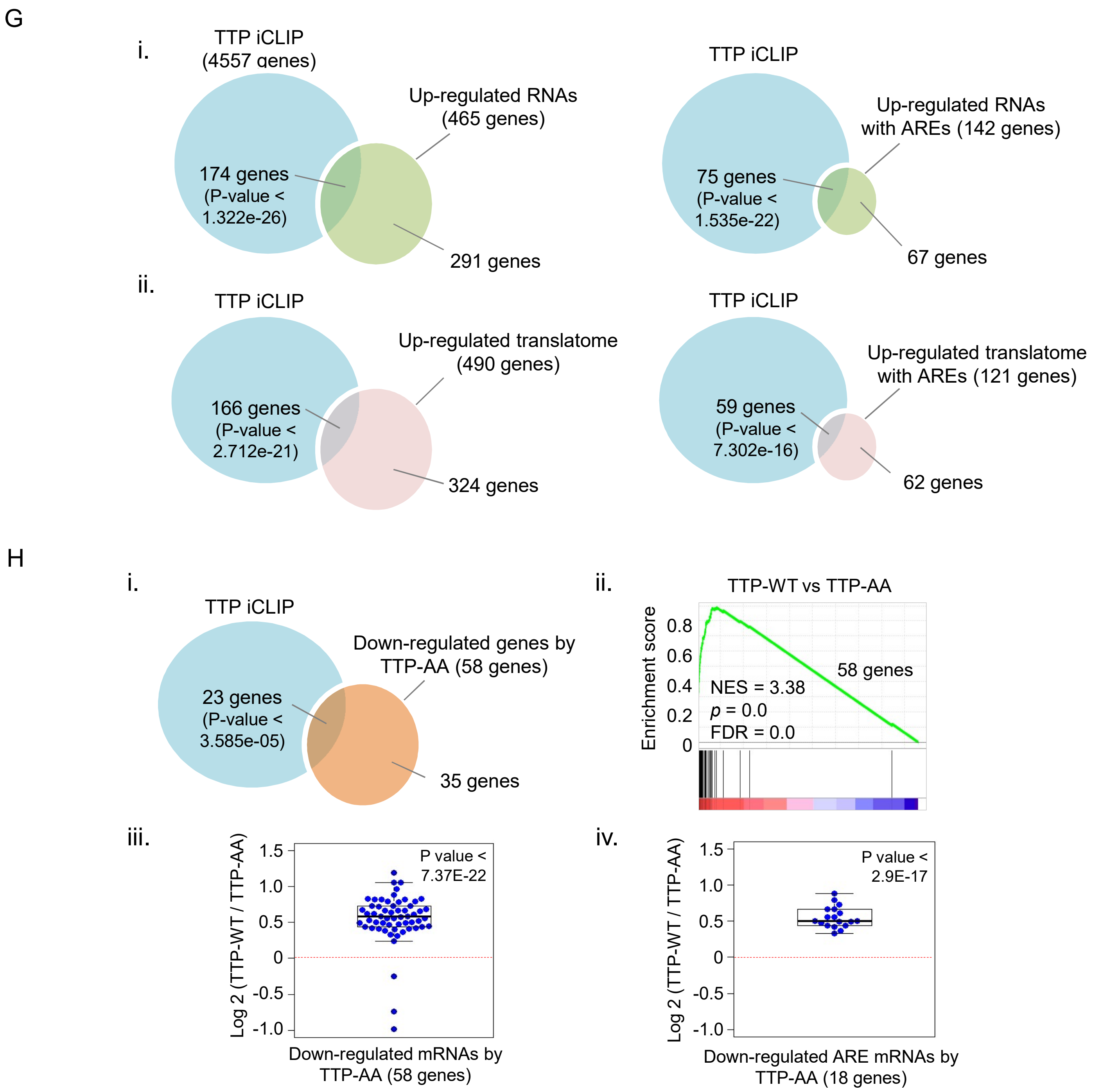
G
i.
TTP iCLIP
(4557 genes)
Up-regulated RNAs
(465 genes)
174 genes
(P-value <
1.322e-26)
291 genes
TTP iCLIP
Up-regulated RNAs
with AREs (142 genes)
75 genes
(P-value <
1.535e-22)
67 genes
ii.
TTP iCLIP
Up-regulated translatome
(490 genes)
166 genes
(P-value <
2.712e-21)
324 genes
TTP iCLIP
Up-regulated translatome
with AREs (121 genes)
59 genes
(P-value <
7.302e-16)
62 genes
H
i.
TTP iCLIP
Down-regulated genes by
TTP-AA (58 genes)
23 genes
(P-value <
3.585e-05)
35 genes
ii.
TTP-WT vs TTP-AA
Enrichment score
0.8
0.6
0.4
0.2
0
58 genes
NES = 3.38
p = 0.0
FDR = 0.0
iii.
Log 2 (TTP-WT / TTP-AA)
1.5
1.0
0.5
0
-0.5
-1.0
P value <
7.37E-22
Down-regulated mRNAs by
TTP-AA (58 genes)
iv.
Log 2 (TTP-WT / TTP-AA)
1.5
1.0
0.5
0
-0.5
-1.0
P value <
2.9E-17
Down-regulated ARE mRNAs by
TTP-AA (18 genes)

Additional File 1: Fig. S3. Related to main Figure 3. A. Distinct motifs enriched in 5' UTRs of genes where ribosome occupancy is significantly increased (ΔRO↑, top panel) or decreased (ΔRO↓, bottom panel) in G0 chemoresistant cells. **B**. Minimum free energy of RNA secondary structure and length of 5'UTRs of genes. **C**. Expression of genes involved in the decay of ARE mRNAs in SS THP1 (top) or SS MCF7 (bottom) compared to S+ cells is shown by GSEA. **D.** Expression of ARE-binding proteins in G0 leukemic cells are shown as a heatmap. These proteins are known to cause ARE mRNA decay or translation repression. **E.** Heatmap of the expression of exosome complex genes (3'-5' exonuclease RNA decay and processing complex) in G0 cancer cells. **F.** Boxplot showing reduced expression of proteasome complex genes in G0 leukemic cells. **G.** (i) Venn diagram comparison of TTP targets (TTP-iCLIP) (14) with the translatome or ARE-bearing translatome up-regulated in G0 cells and (ii) of TTP targets with the mRNAs or ARE mRNAs up-regulated in G0 cells. **H.** Transcriptome profiles using microarray, of AraC-treated cells knocked out for endogenous TTP and expressing either TTP-AA or TTP-WT. (i) Venn diagram of TTP targets and the transcriptome downregulated by TTP-AA compared to TTP-deficient cells in AraCS cells. (ii) GSEA of 58 genes down-regulated by TTP-AA in cells expressing TTP-WT compared to TTP-AA. (iii) Relative RNA levels of 58 genes targeted by TTP-AA in cells expressing TTP-WT compared to TTP-AA. (iv) Relative RNA levels of 18 ARE-bearing genes targeted by TTP-AA in cells expressing TTP-WT compared to TTP-AA. Data are represented as average ± SEM.

Figure S4

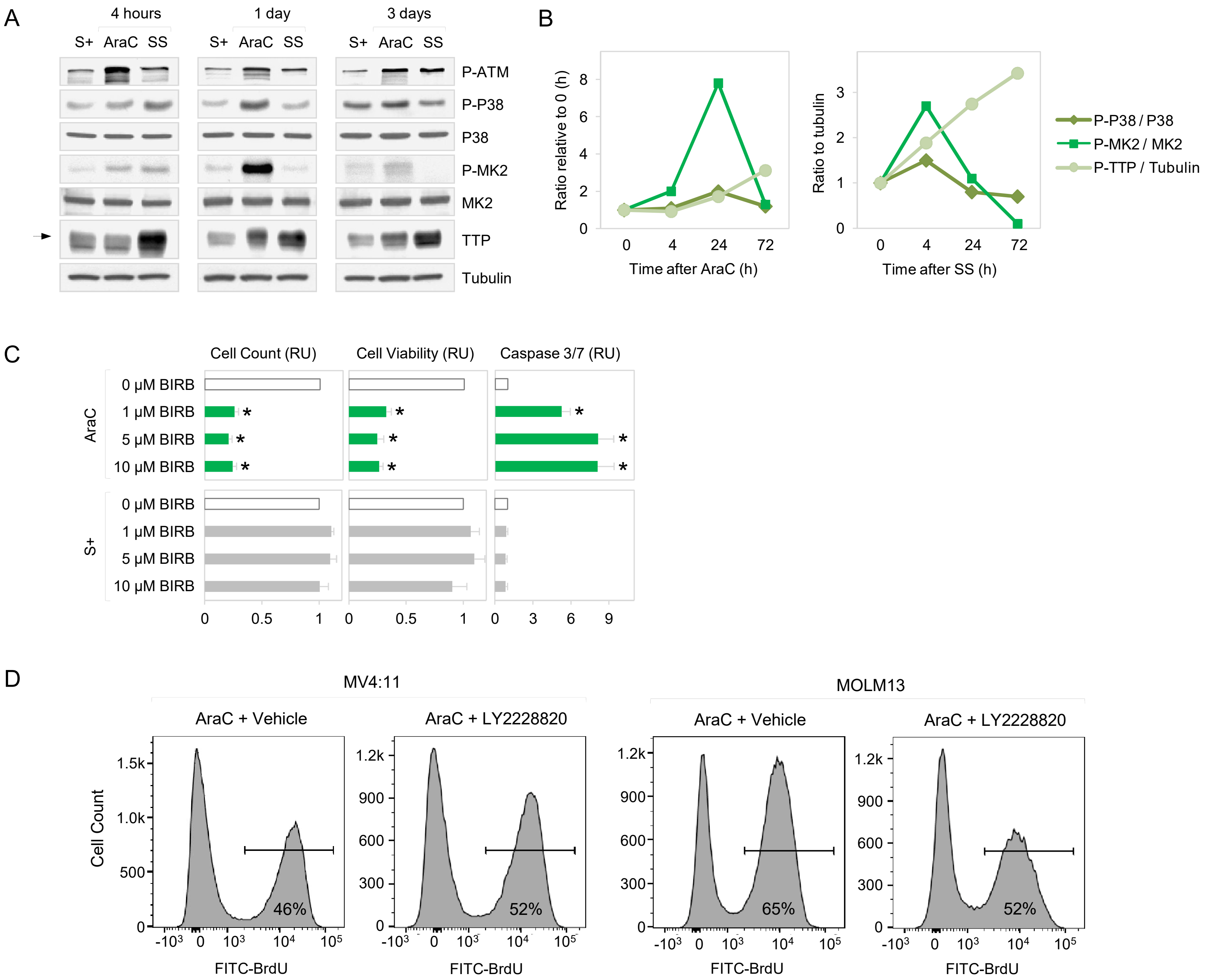
A
4 hours
1 day
3 days
S+ AraC SS
P-ATM
P-P38
P38
P-MK2
MK2
TTP
Tubulin
B
Ratio relative to 0 (h)
Time after AraC (h)
Ratio to tubulin
Time after SS (h)
P-P38 / P38
P-MK2 / MK2
P-TTP / Tubulin
C
Cell Count (RU)
Cell Viability (RU)
Caspase 3/7 (RU)
AraC
S+
0 μM BIRB
1 μM BIRB
5 μM BIRB
10 μM BIRB
D
MV4:11
MOLM13
AraC + Vehicle
AraC + LY2228820
Cell Count
FITC-BrdU
46%
52%
65%
52%

Additional File 1: Fig. S4. Related to main Figures 4-6. A. Western analysis of indicated proteins in THP1 cells at time points after serum starvation or 5 μM AraC treatment. **B**. Relative ratio of phospho-p38 MAPK to total p38 MAPK, phospho-MK2 to total MK2, or TTP to tubulin (loading control), at indicated time points upon AraC or SS treatment. **C**. Effect of p38 MAPK inhibition on survival of AraC-resistant cells. MOLM13 leukemic cells were pre-treated with various concentrations of BIRB796 (BIRB or BB), followed by AraC or vehicle treatment. Cell viability and death were assessed by cell counting, MTS and caspase 3/7 assays. RU=relative units. **D.** Flow cytometric profiles of MV4:11 or MOLM13 cells treated with AraC or AraC plus LY2228820 for one day. Percentages of BrdU-positive cells are shown. *$p \leq 0.05$ Data are represented as average ± SEM.

Figure S5

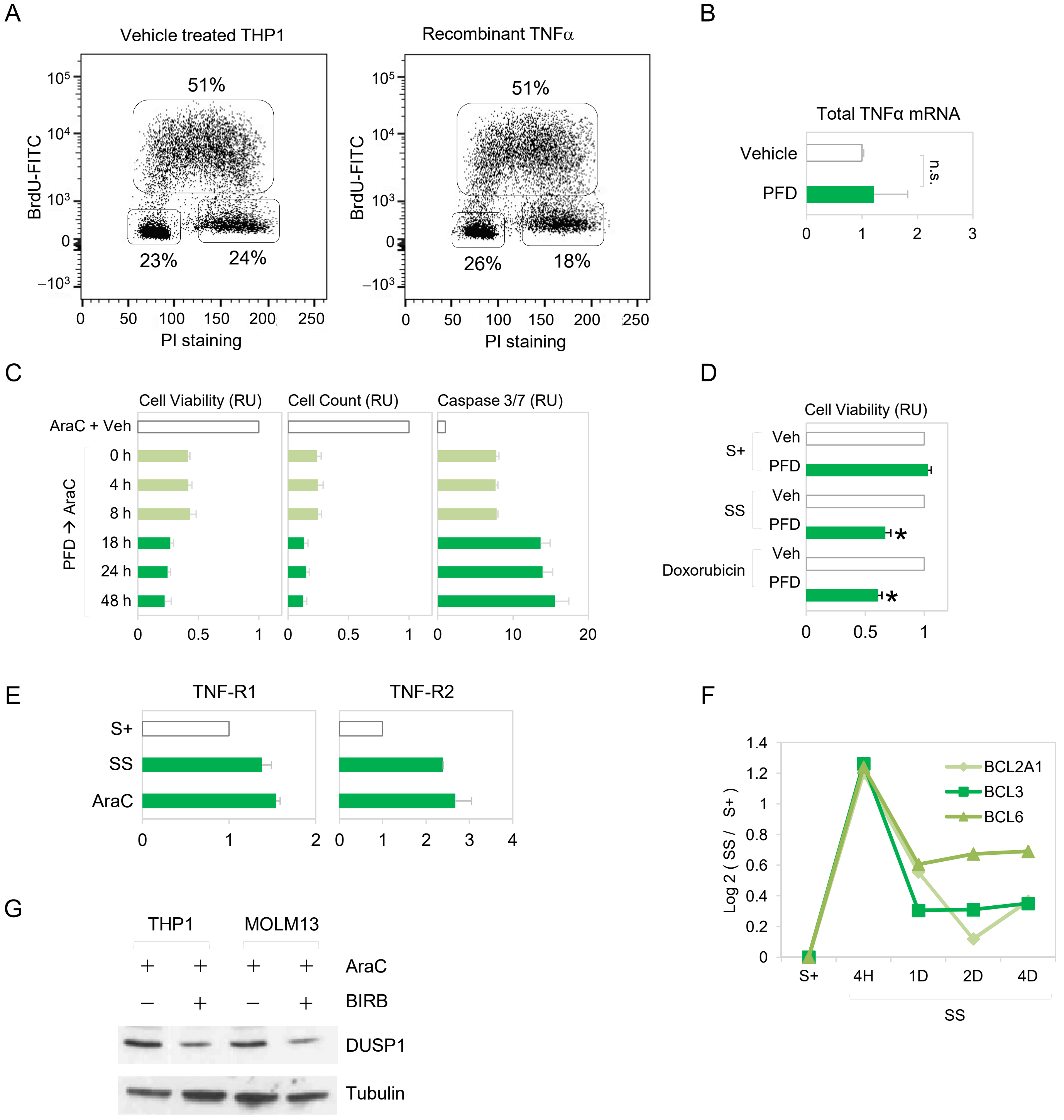
A
Vehicle treated THP1
Recombinant TNFα
51%
23%
24%
51%
26%
18%
BrdU-FITC
PI staining
B
Total TNFα mRNA
Vehicle
PFD
n.s.
C
Cell Viability (RU)
Cell Count (RU)
Caspase 3/7 (RU)
AraC + Veh
0 h
4 h
8 h
18 h
24 h
48 h
PFD → AraC
D
Cell Viability (RU)
S+
SS
Doxorubicin
Veh
PFD
E
TNF-R1
TNF-R2
S+
SS
AraC
F
Log 2 (SS / S+)
BCL2A1
BCL3
BCL6
S+
4H
1D
2D
4D
SS
G
THP1
MOLM13
AraC
BIRB
DUSP1
Tubulin

H

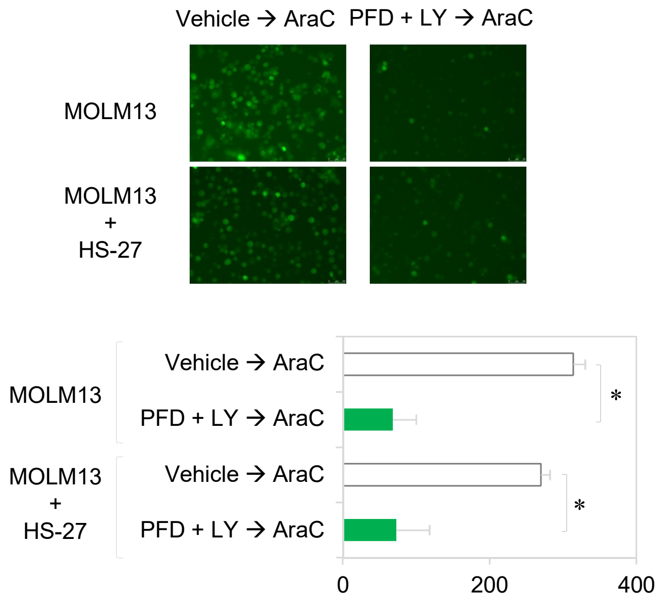
Vehicle → AraC
PFD + LY → AraC
MOLM13
MOLM13
+
HS-27
Vehicle → AraC
PFD + LY → AraC
Vehicle → AraC
PFD + LY → AraC
*
*
0
200
400

I

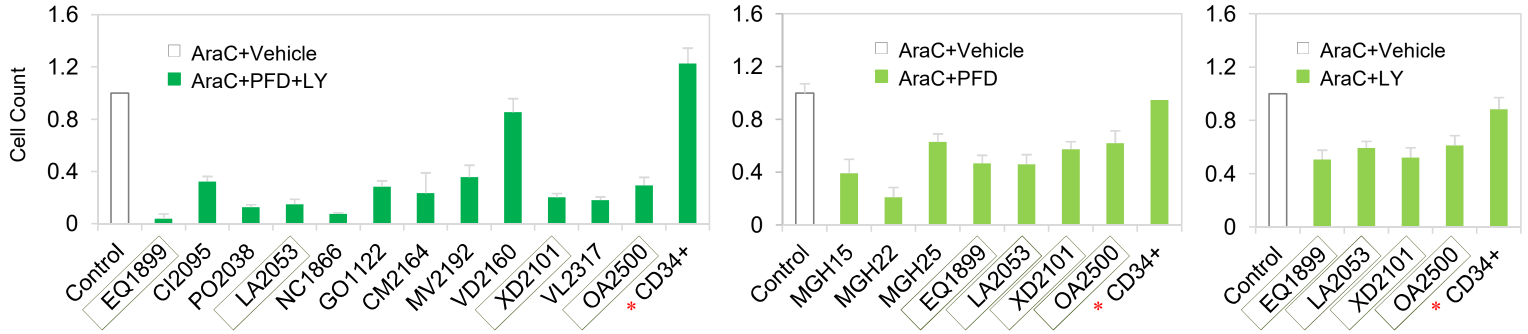
Cell Count
1.6
1.2
0.8
0.4
0
AraC+Vehicle
AraC+PFD+LY
Control
EQ1899
CI2095
PO2038
LA2053
NC1866
GO1122
CM2164
MV2192
VD2160
XD2101
VL2317
OA2500
CD34+
*
AraC+Vehicle
AraC+PFD
Control
MGH15
MGH22
MGH25
EQ1899
LA2053
XD2101
OA2500
CD34+
*
AraC+Vehicle
AraC+LY
Control
EQ1899
LA2053
XD2101
OA2500
CD34+
*

J

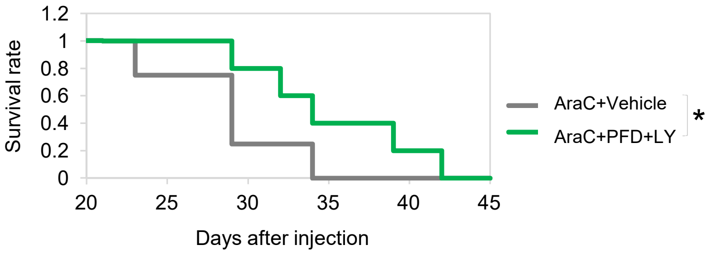
Survival rate
1.2
1
0.8
0.6
0.4
0.2
0
20
25
30
35
40
45
Days after injection
AraC+Vehicle
AraC+PFD+LY
*

Additional File 1: Fig. S5. Related to main Figures 5-7. **A**. Flow cytometric profiles of THP1 cells treated with vehicle or recombinant TNFα. **B**. TNFα RNA levels in SS cells treated with vehicle or PFD. **C**. Effect of PFD on survival of AraC-resistant cells. THP1 cells pre-treated with 300 μg/ml PFD for indicated time followed by 5 μM AraC. Cell viability and death were measured. **D.** MCF7 cells were pre-treated with 300 μg/ml PFD or vehicle 1 day before treatment with serum starvation or 150 nM doxorubicin. Cell viability of PFD-treated compared to vehicle-treated cells is shown. **E.** TNF-R1 and TNF-R2 expression at the translatome level. **F**. Translatome expression of BCL2A1, BCL3 and BCL6 at indicated time points after serum starvation. **G.** Western blot of DUSP1 in AraCS THP1 and MOLM13 cells treated with BIRB or vehicle. Tubulin serves as loading control. **H.** MOLM13-GFP-luciferase cells were cultured with or without HS-27 cells and treated with PLA therapy or AraC. Representative microscopic images (top panel) and quantification of luciferase activity (bottom panel) are shown. **I**. Viability of primary cells from AML patients and normal CD34+ cells (marked by a red asterisk) from healthy donors after indicated treatments, normalized to DMSO+AraC treatment for each patient sample (represented as a white bar and set to 1). Patient samples that were compared with individual treatments and PLA and shown in Fig. 8A are marked by a box. **J**. Kaplan-Meier survival curves of C57BL/6 mice engrafted with primary HOXA9/Meis1 cells and treated with PLA therapy or AraC in figure 8F. $^*p \leq 0.05$ Data are represented as average ± SEM.

Figure S6

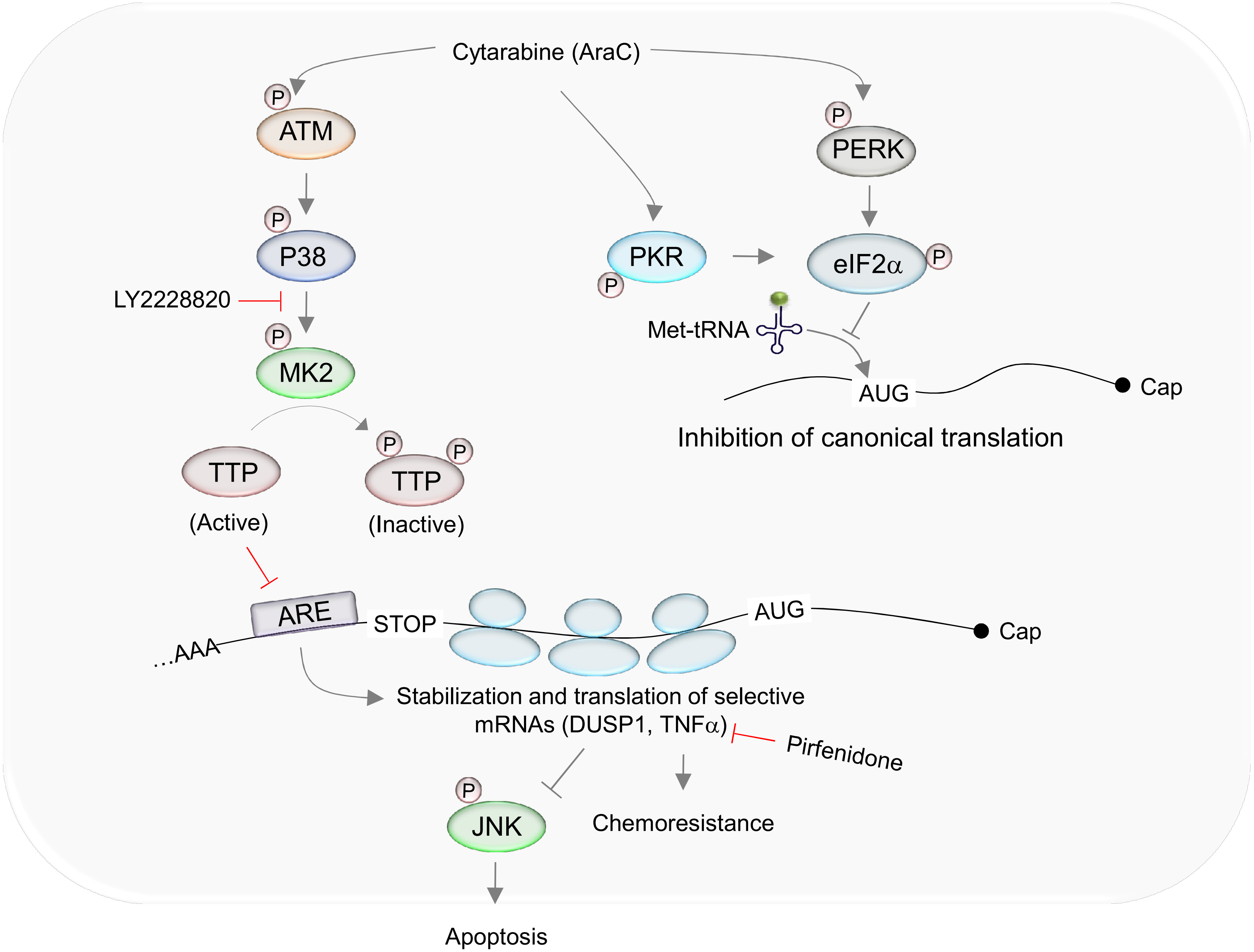

Cytarabine (AraC)
P
ATM
P
P38
LY2228820
P
MK2
P
PKR
P
PERK
eIF2α
P
Met-tRNA
AUG
Cap
Inhibition of canonical translation
TTP
(Active)
P
P
TTP
(Inactive)
ARE
...AAA
STOP
AUG
Cap
Stabilization and translation of selective
mRNAs (DUSP1, TNFα)
Pirfenidone
P
JNK
Chemoresistance
Apoptosis

Additional File 1: Fig. S6. Related to main Figures 1-8. A model for chemoresistance in AML. Post-transcriptional and translational regulation of gene expression leads to chemoresistance and G0 cell survival, and is regulated by DNA damage and stress signaling, triggered in subpopulations of cancer by chemotherapy and serum-starvation. AraC or SS treatment induces and enriches for quiescent, chemoresistant leukemic cells. Even though canonical translation is inhibited, ARE-bearing mRNAs are increased and highly translated in G0 cells. Mechanistically, the p38 MAPK-MK2 pathway stabilizes ARE mRNAs such as TNFα and DUSP1 via phosphorylation of TTP and inactivation of its RNA decay activity. DUSP1 inhibits JNK-mediated apoptosis and TNFα increases anti-apoptotic genes and cell survival, leading to chemoresistance. Inhibition of ARE mRNA expression by p38 MAPK and TNFα inhibitors or TTP-AA mutant sensitizes resistant leukemic cells to AraC treatment.

Legend for Additional File 2: Table S1. Related to main Figures 1-8. RNAs up-regulated at the translatome level and their RO changes in G0 cells induced by SS or AraC.

Legend for Additional File 3: Table S2. Related to main Figures 1-8. RNAs bearing AU-rich elements (AREs) and up-regulated at the translatome level in G0 cells induced by SS or AraC.

References

1. Forbes SA, Beare D, Gunasekaran P, Leung K, Bindal N, Boutselakis H, et al. COSMIC: exploring the world's knowledge of somatic mutations in human cancer. Nucleic Acids Res. 2015;43(Database issue):D805-D11.

2. Saito Y, Kitamura H, Hijikata A, Tomizawa-Murasawa M, Tanaka S, Takagi S, et al. Identification of therapeutic targets for quiescent, chemotherapy-resistant human leukemia stem cells. Sci Transl Med. 2010;2(17):17ra9.

3. Hay N, Sonenberg N. Upstream and downstream of mTOR. Genes Dev. 2004;18(16):1926-45.

4. Koritzinsky M, Magagnin MG, van den BT, Seigneuric R, Savelkouls K, Dostie J, et al. Gene expression during acute and prolonged hypoxia is regulated by distinct mechanisms of translational control. EMBO J. 2006;25(5):1114-25.

5. Sonenberg N, Hinnebusch AG. Regulation of translation initiation in eukaryotes: mechanisms and biological targets. Cell. 2009;136(4):731-45.

6. Villalonga P, Fernandez de Mattos S, Ridley AJ. RhoE inhibits 4E-BP1 phosphorylation and eIF4E function impairing cap-dependent translation. J Biol Chem. 2009;284(51):35287-96.

7. Caron E, Ghosh S, Matsuoka Y, Ashton-Beaucage D, Therrien M, Lemieux S, et al. A comprehensive map of the mTOR signaling network. Mol Syst Biol. 2010;6:453.

8. Foster KG, Fingar DC. Mammalian target of rapamycin (mTOR): conducting the cellular signaling symphony. J Biol Chem. 2010;285(19):14071-7.

9. Levin VA, Panchabhai SC, Shen L, Kornblau SM, Qiu Y, Baggerly KA. Different changes in protein and phosphoprotein levels result from serum starvation of high-grade glioma and adenocarcinoma cell lines. Journal of proteome research. 2010;9(1):179-91.

10. Pirkmajer S, Chibalin AV. Serum starvation: caveat emptor. American journal of physiology Cell physiology. 2011;301(2):C272-9.

11. Nawroth R, Stellwagen F, Schulz WA, Stoehr R, Hartmann A, Krause BJ, et al. S6K1 and 4E-BP1 Are Independent Regulated and Control Cellular Growth in Bladder Cancer. PLOS ONE. 2011;6(11):e27509.

12. Batool A, Aashaq S, Andrabi KI. Reappraisal to the study of 4E-BP1 as an mTOR substrate - A normative critique. Eur J Cell Biol. 2017;96(4):325-36.

13. Bodur C, Kazyken D, Huang K, Ekim Ustunel B, Siroky KA, Tooley AS, et al. The IKK-related kinase TBK1 activates mTORC1 directly in response to growth factors and innate immune agonists. Embo j. 2018;37(1):19-38.

14. Tiedje C, Diaz-Munoz MD, Trulley P, Ahlfors H, Laaß K, Blackshear PJ, et al. The RNA-binding protein TTP is a global post-transcriptional regulator of feedback control in inflammation. Nucleic Acids Research. 2016;44(15):7418-40.
