# Supplementary material for: A post-transcriptional program of chemoresistance by AU-rich elements and TTP in quiescent leukemic cells
Source: Genome Biol. 2020 Feb 10;21:33. doi: 10.1186/s13059-020-1936-4 (PMC7011231; doi:10.1186/s13059-020-1936-4)
Supplement: Supplementary file 4 — Uncropped Western blots. [file 13059_2020_1936_MOESM4_ESM.pptx]

## Slide 1
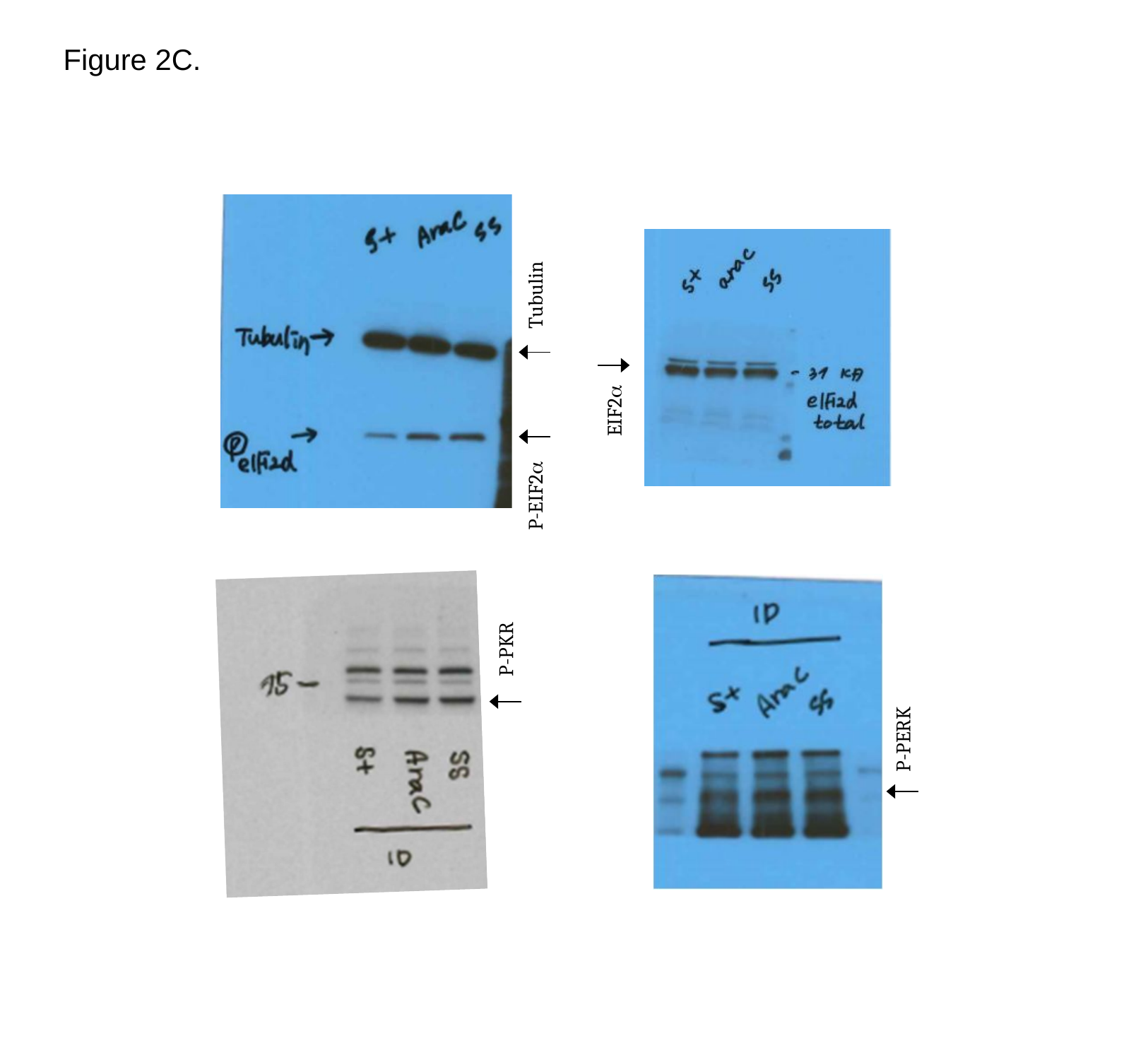

Figure 2C.
Tubulin
EIF2a
P-EIF2a
P-PKR
P-PERK

## Slide 2
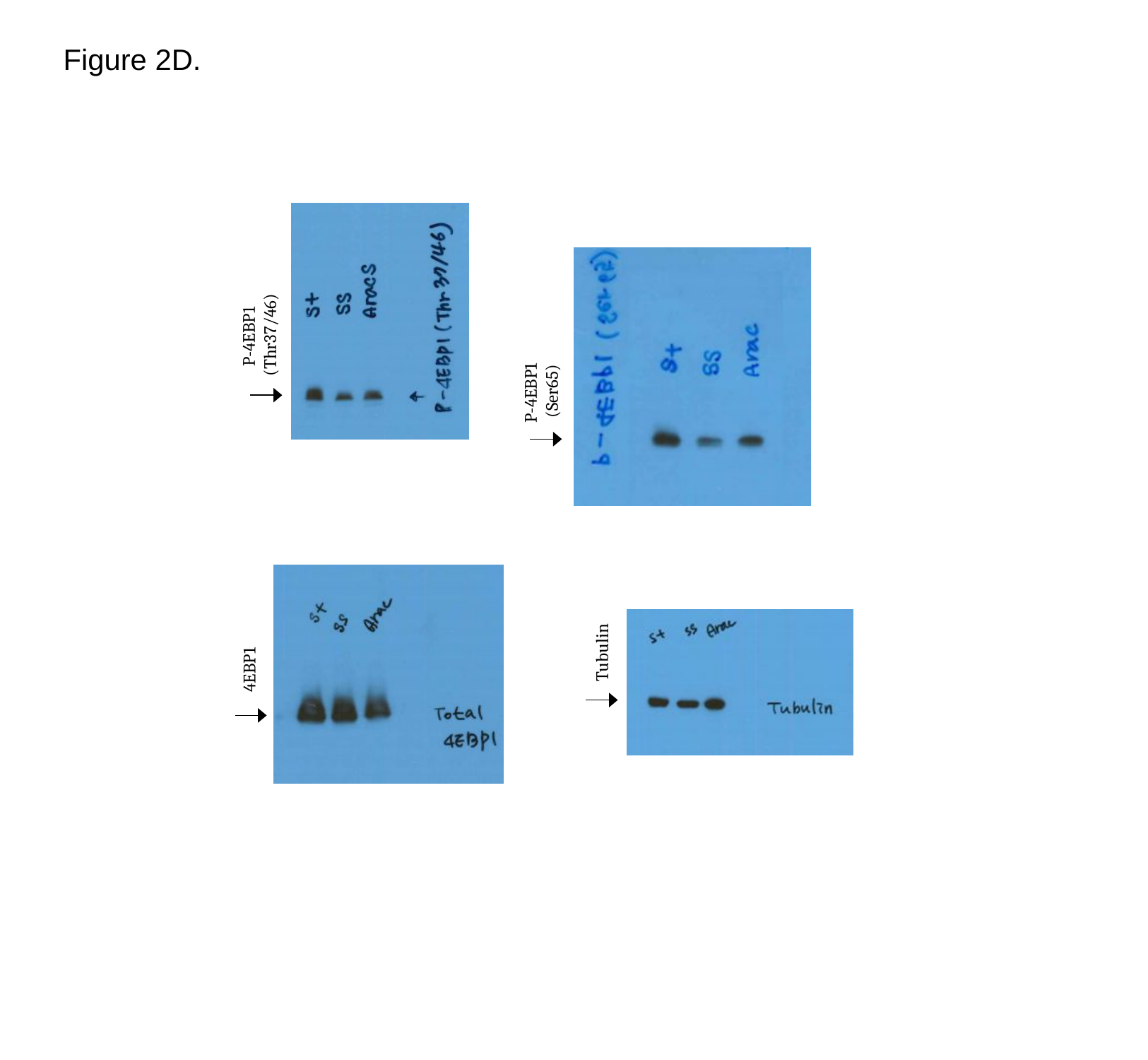

Figure 2D.
P-4EBP1
(Thr37/46)
P-4EBP1
(Ser65)
Tubulin
4EBP1

## Slide 3
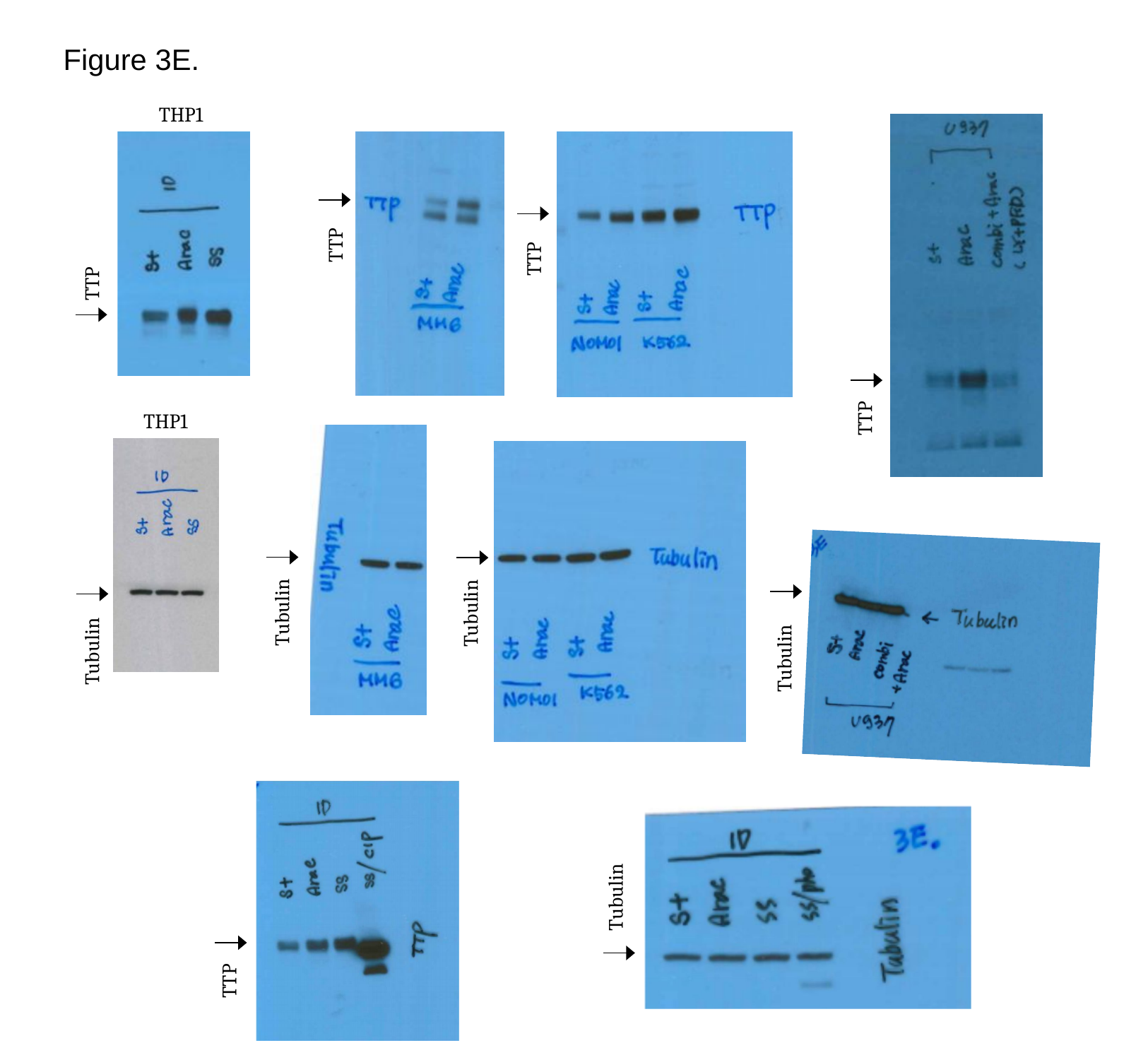

Figure 3E.
THP1
TTP
TTP
TTP
TTP
THP1
Tubulin
Tubulin
Tubulin
Tubulin
Tubulin
TTP

## Slide 4
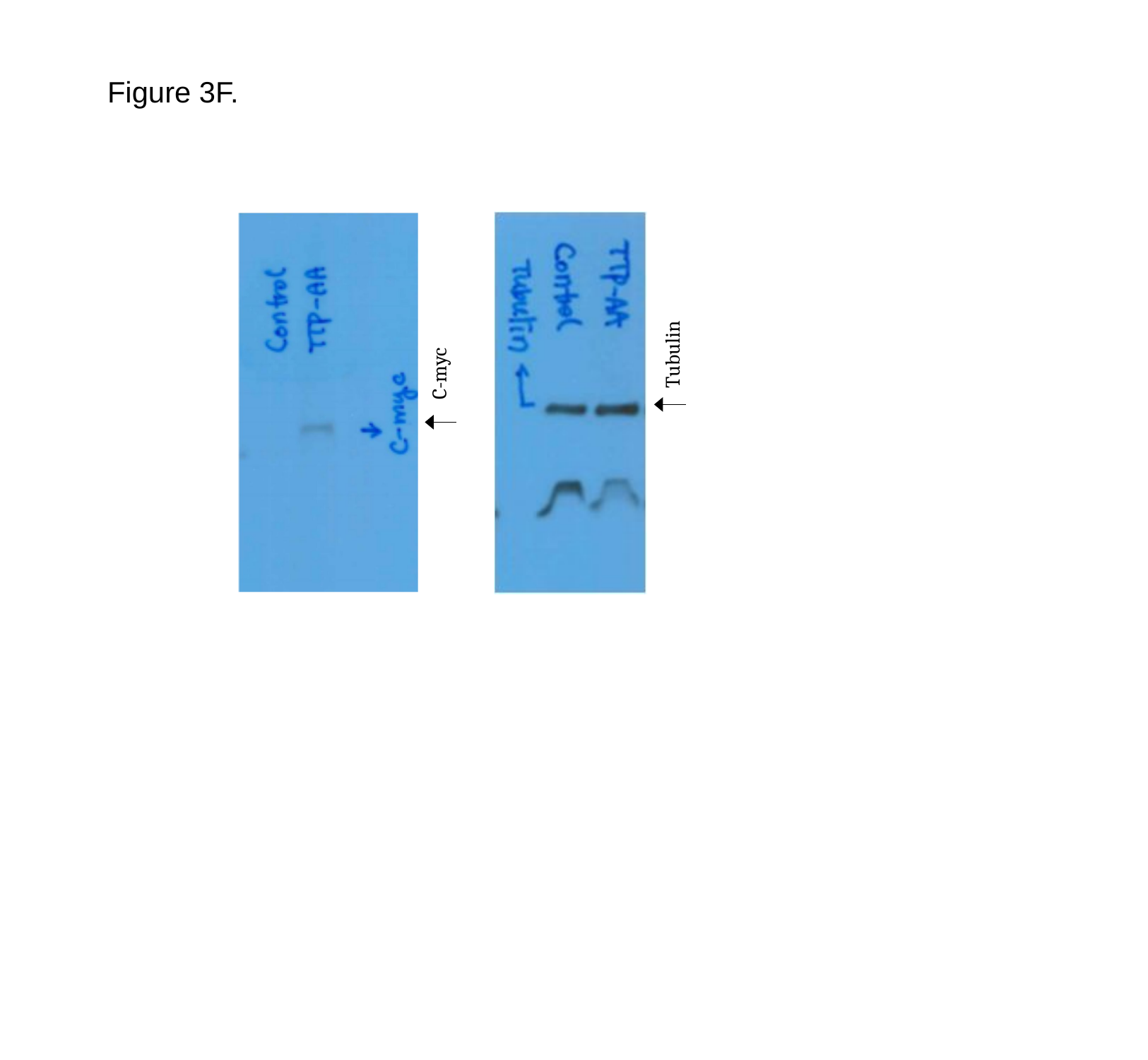

Figure 3F.
Tubulin
C-myc

## Slide 5
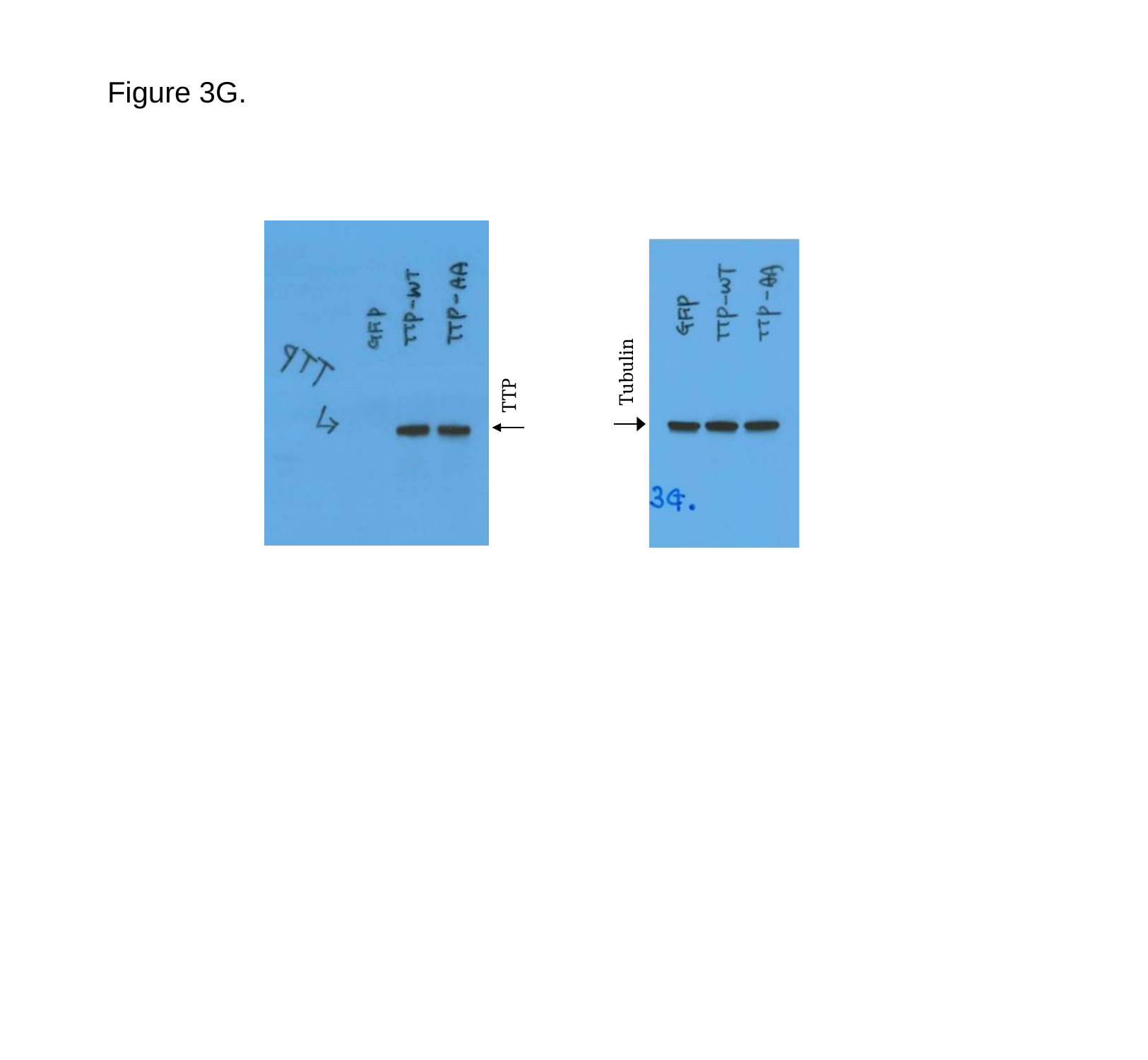

Figure 3G.
Tubulin
TTP

## Slide 6
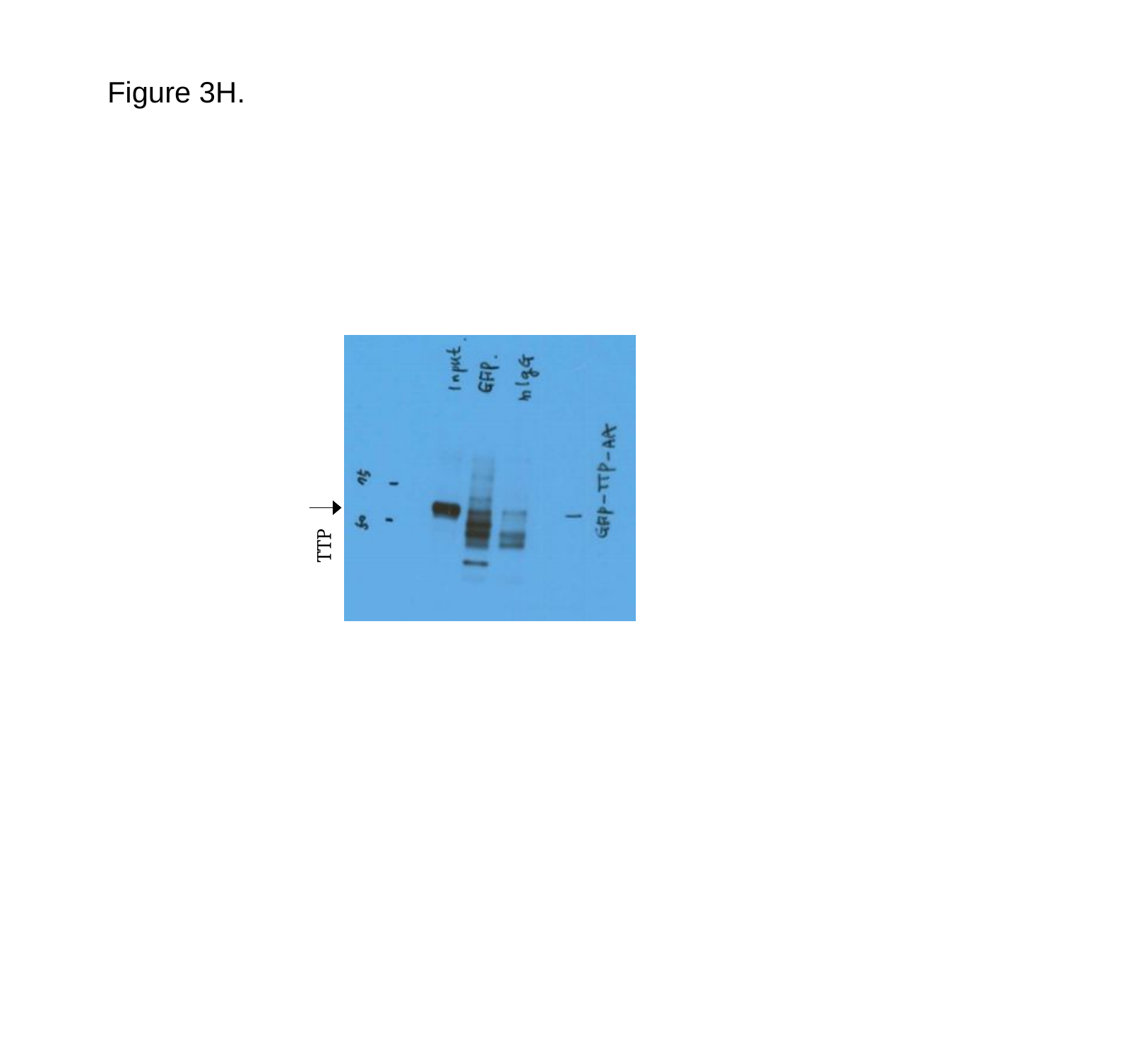

Figure 3H.
TTP

## Slide 7
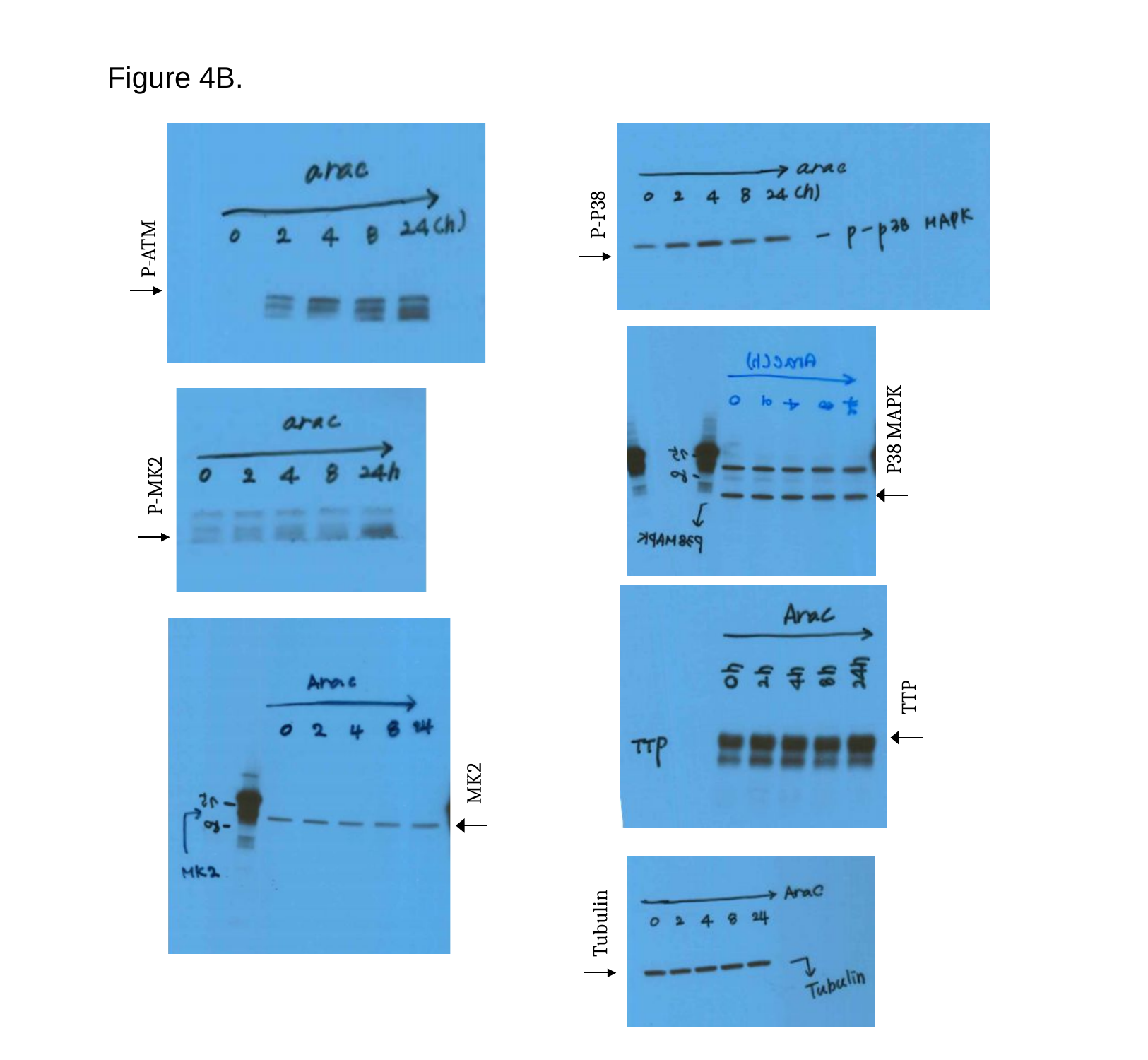

Figure 4B.
P-P38
P-ATM
P38 MAPK
P-MK2
TTP
MK2
Tubulin

## Slide 8
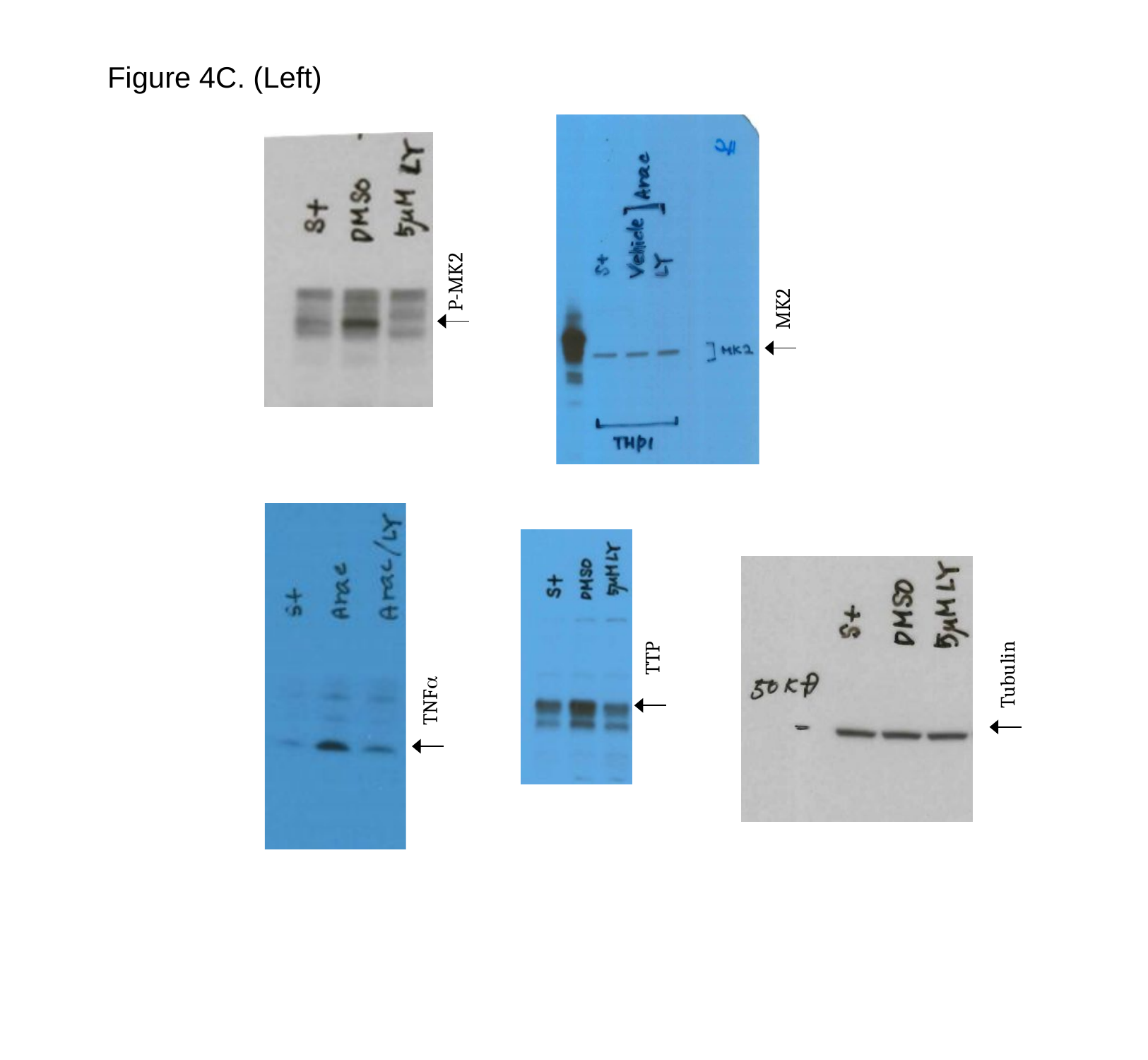

Figure 4C. (Left)
P-MK2
MK2
TTP
Tubulin
TNFa

## Slide 9
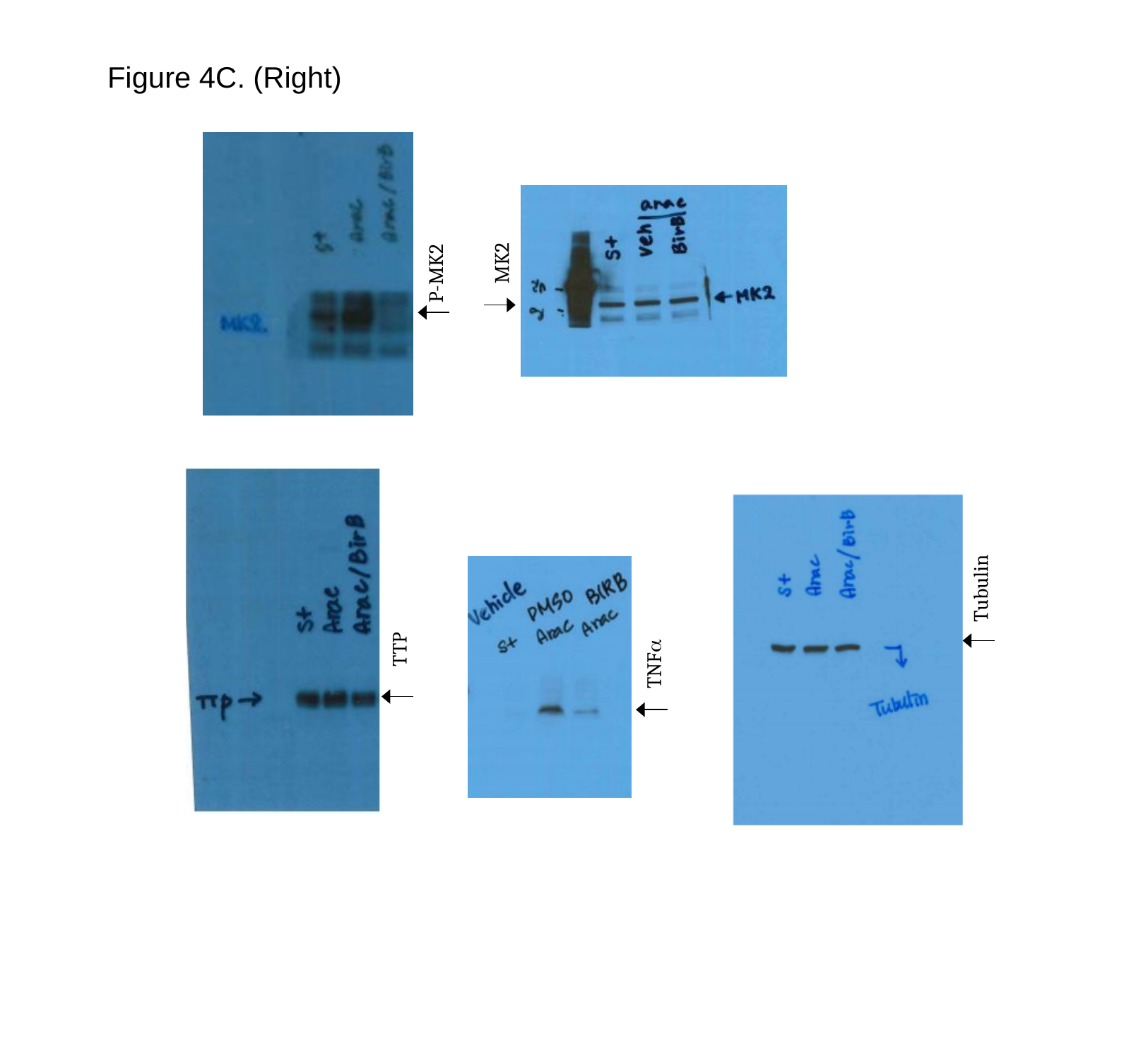

Figure 4C. (Right)
MK2
P-MK2
Tubulin
TTP
TNFa

## Slide 10
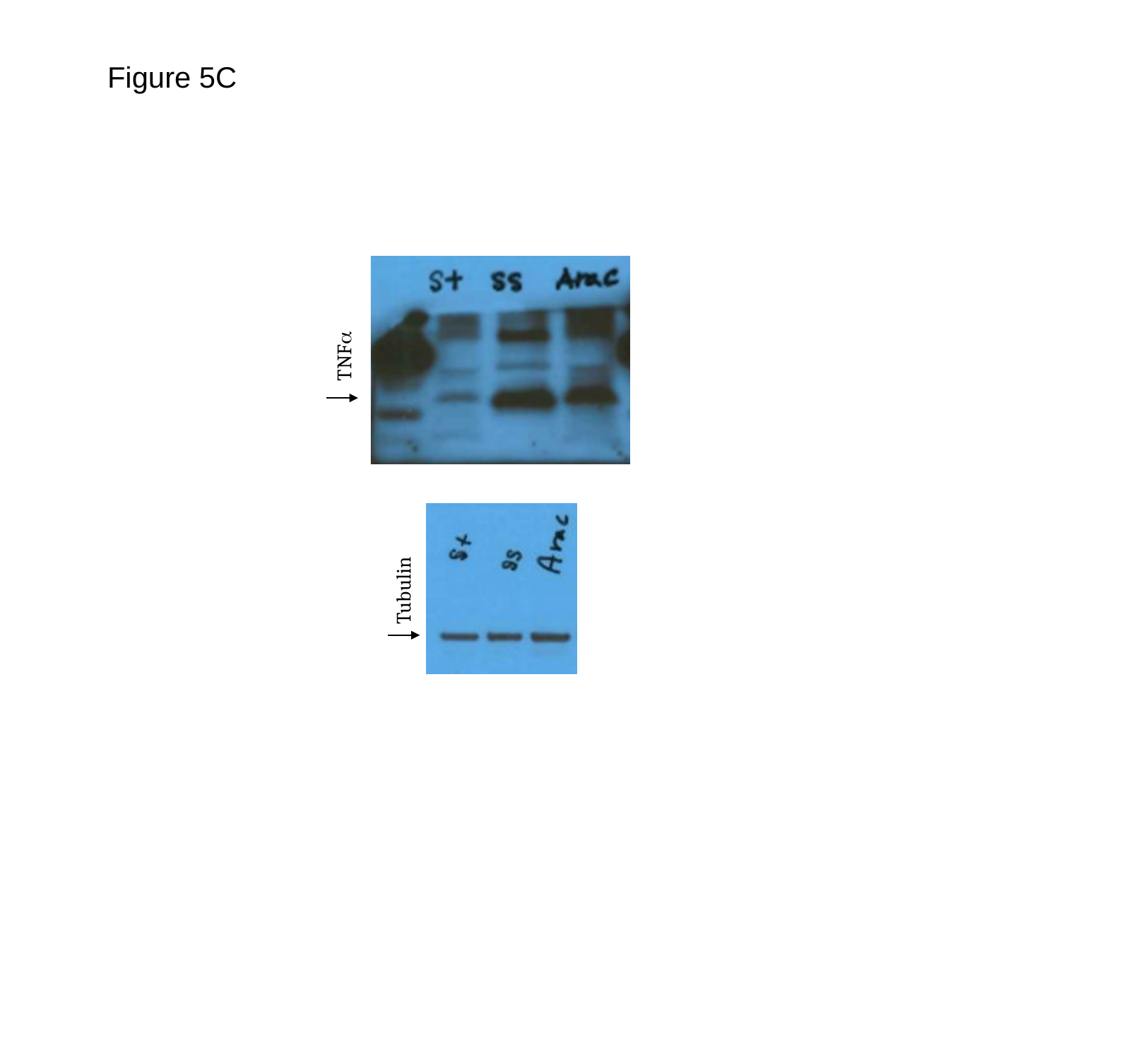

Figure 5C
TNFa
Tubulin

## Slide 11
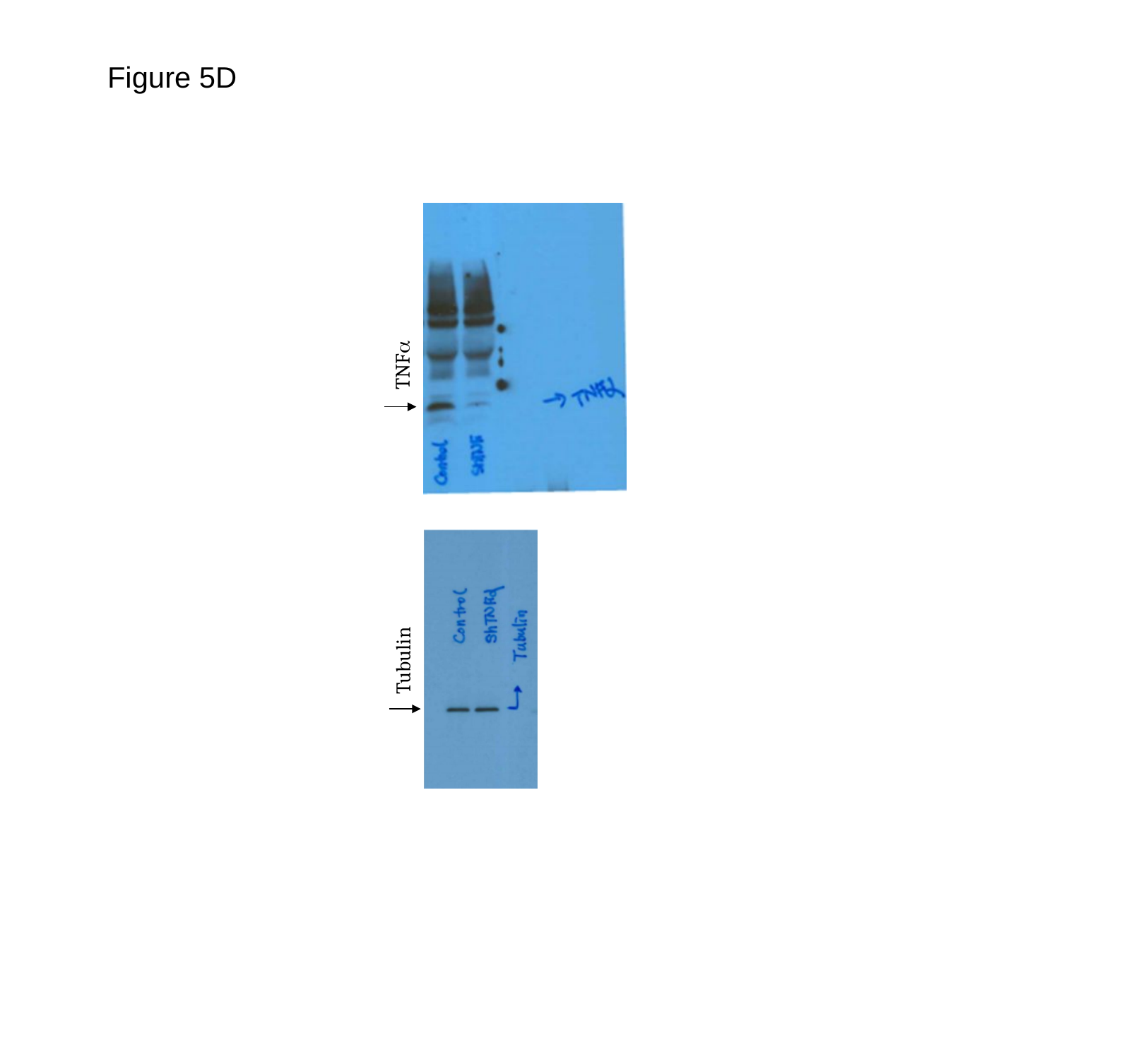

Figure 5D
TNFa
Tubulin

## Slide 12
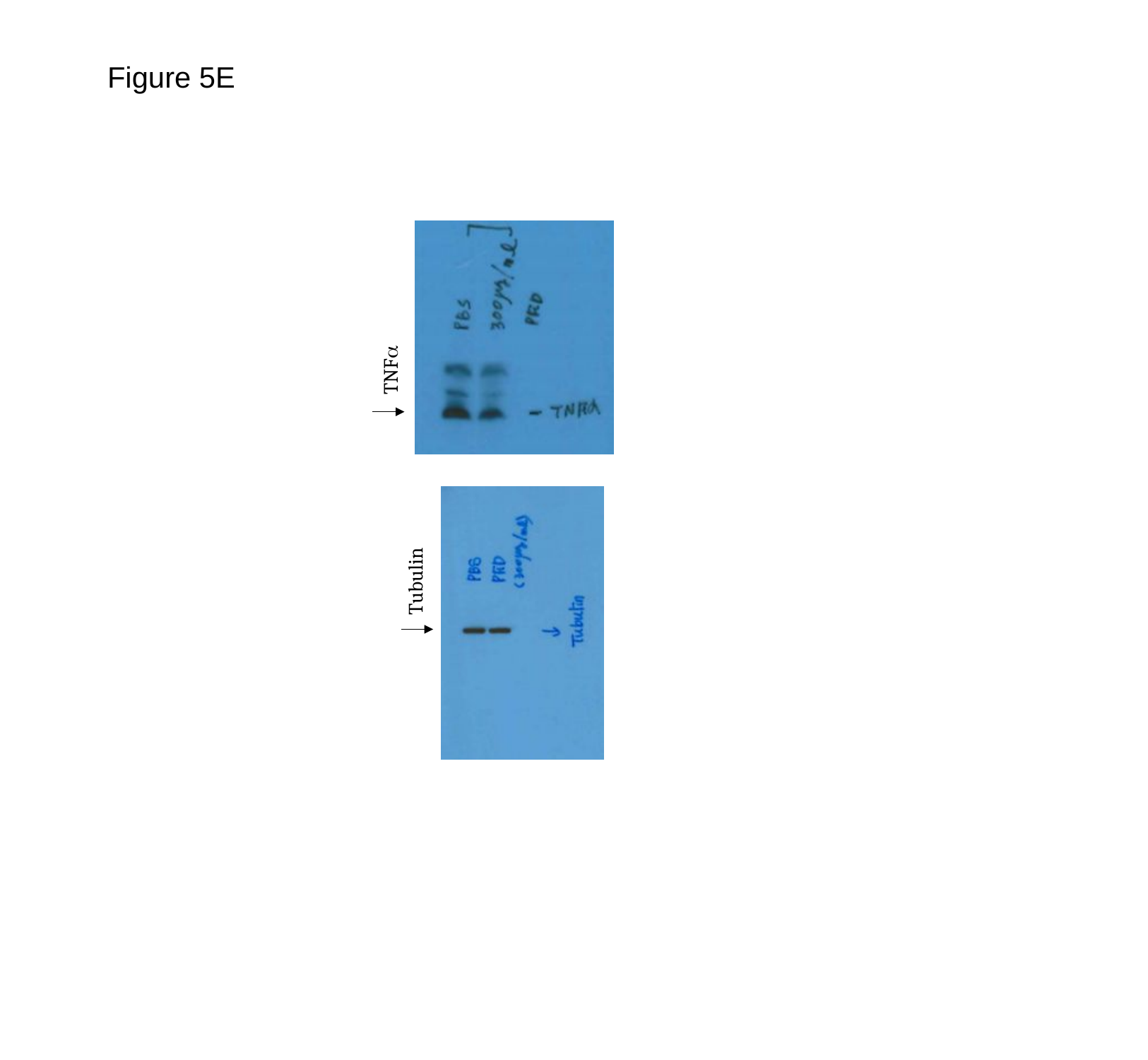

Figure 5E
TNFa
Tubulin

## Slide 13
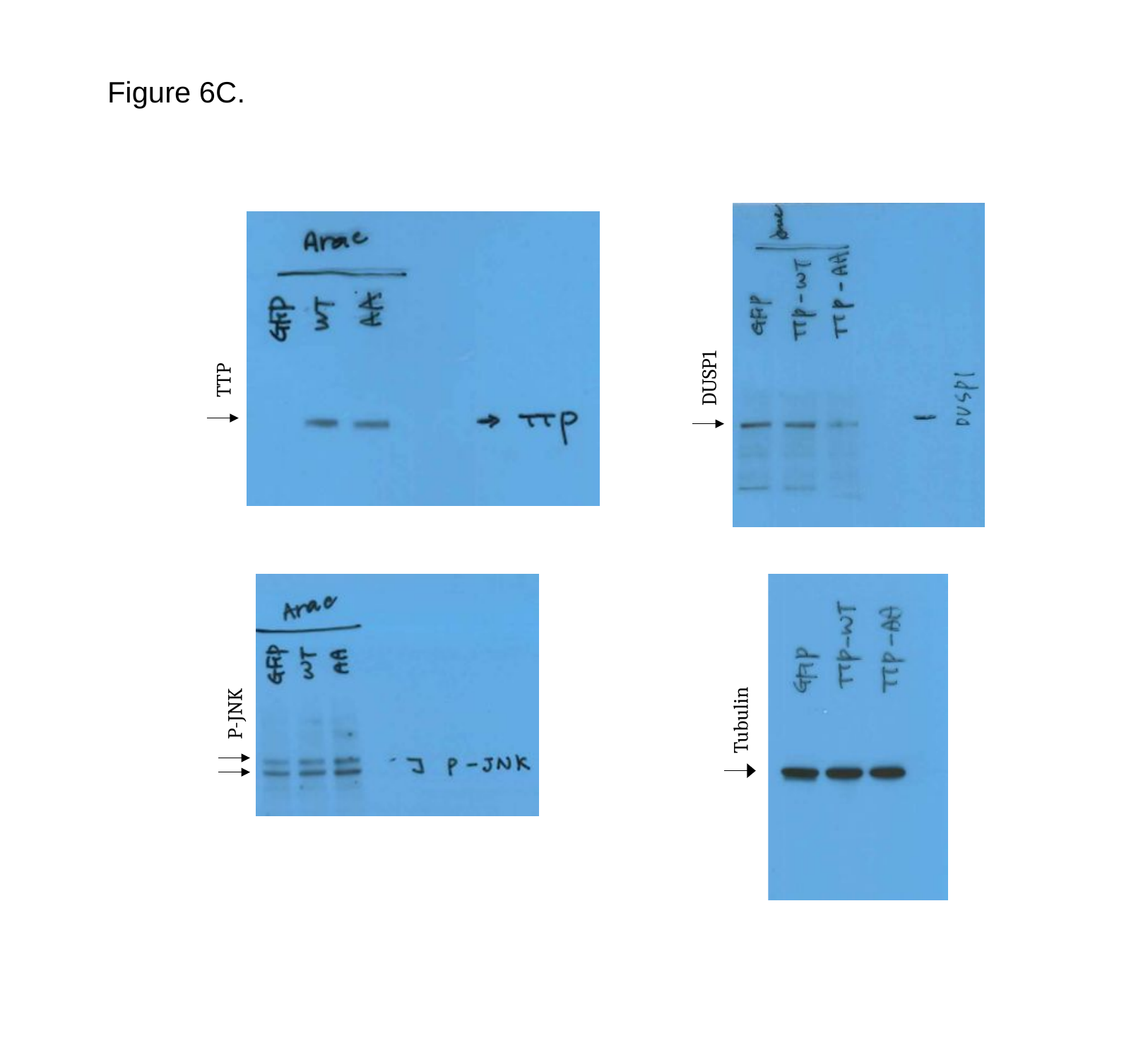

Figure 6C.
DUSP1
TTP
P-JNK
Tubulin

## Slide 14
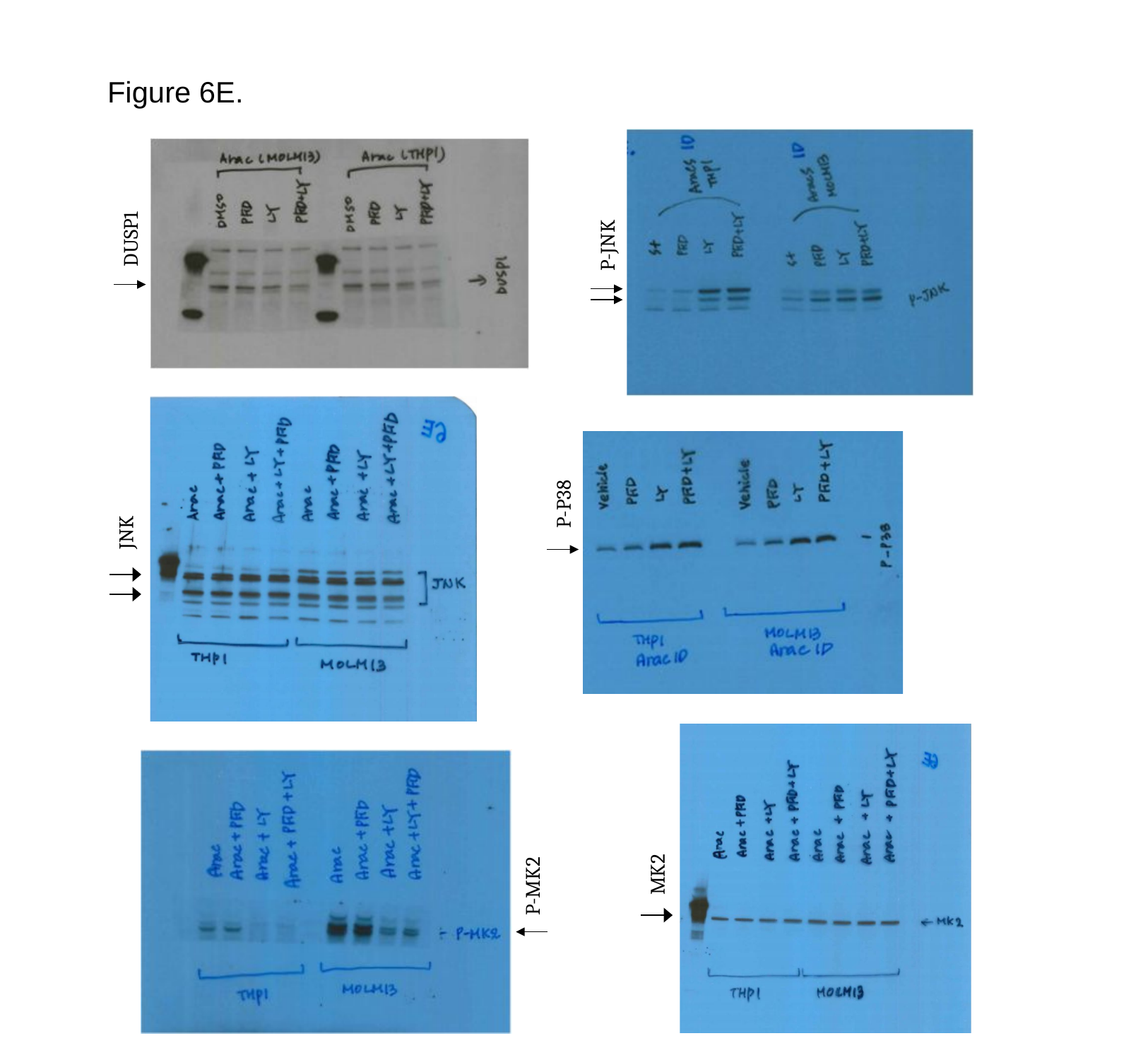

Figure 6E.
DUSP1
P-JNK
P-P38
JNK
MK2
P-MK2

## Slide 15
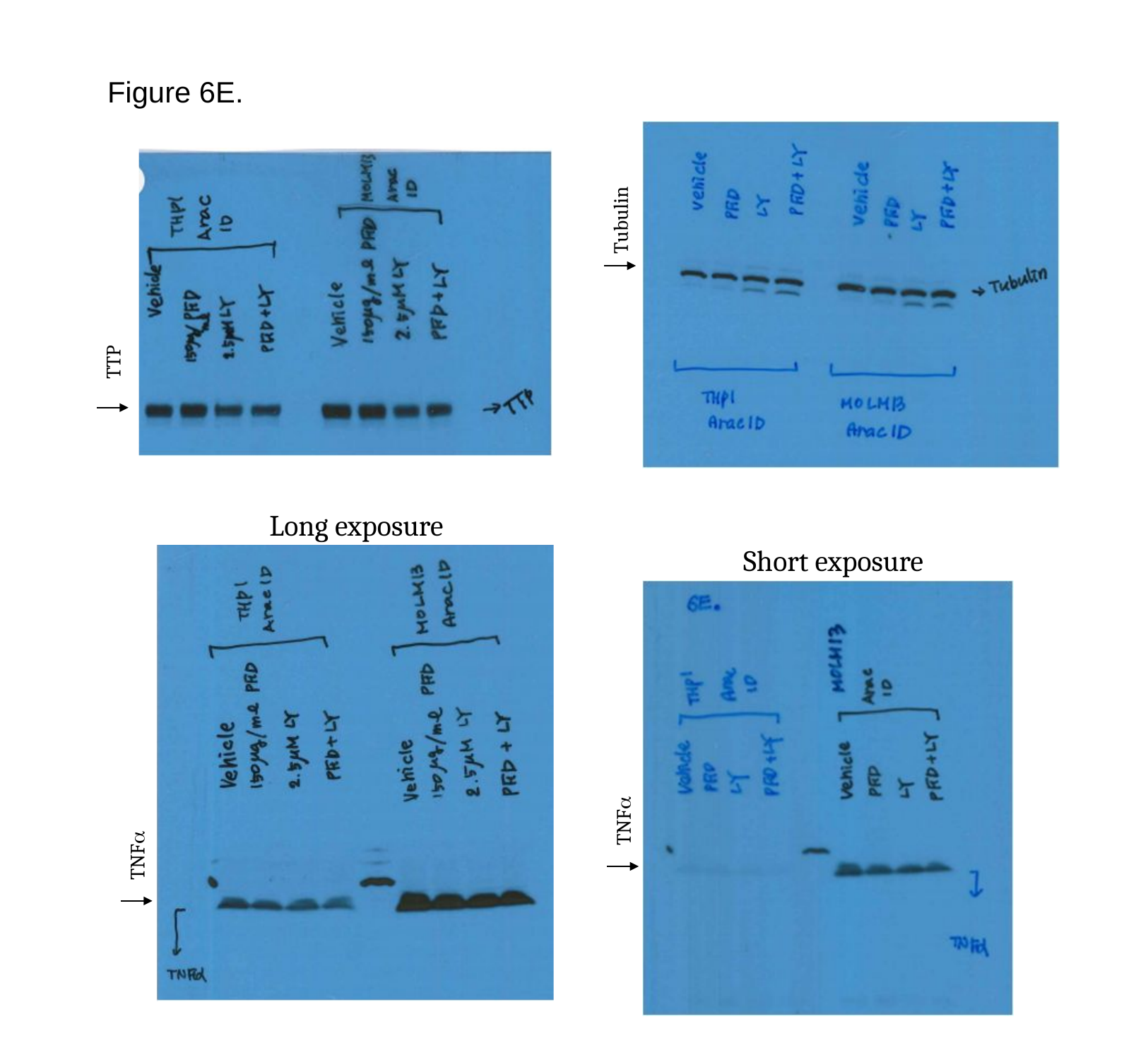

Figure 6E.
Tubulin
TTP
Long exposure
Short exposure
TNFa
TNFa

## Slide 16
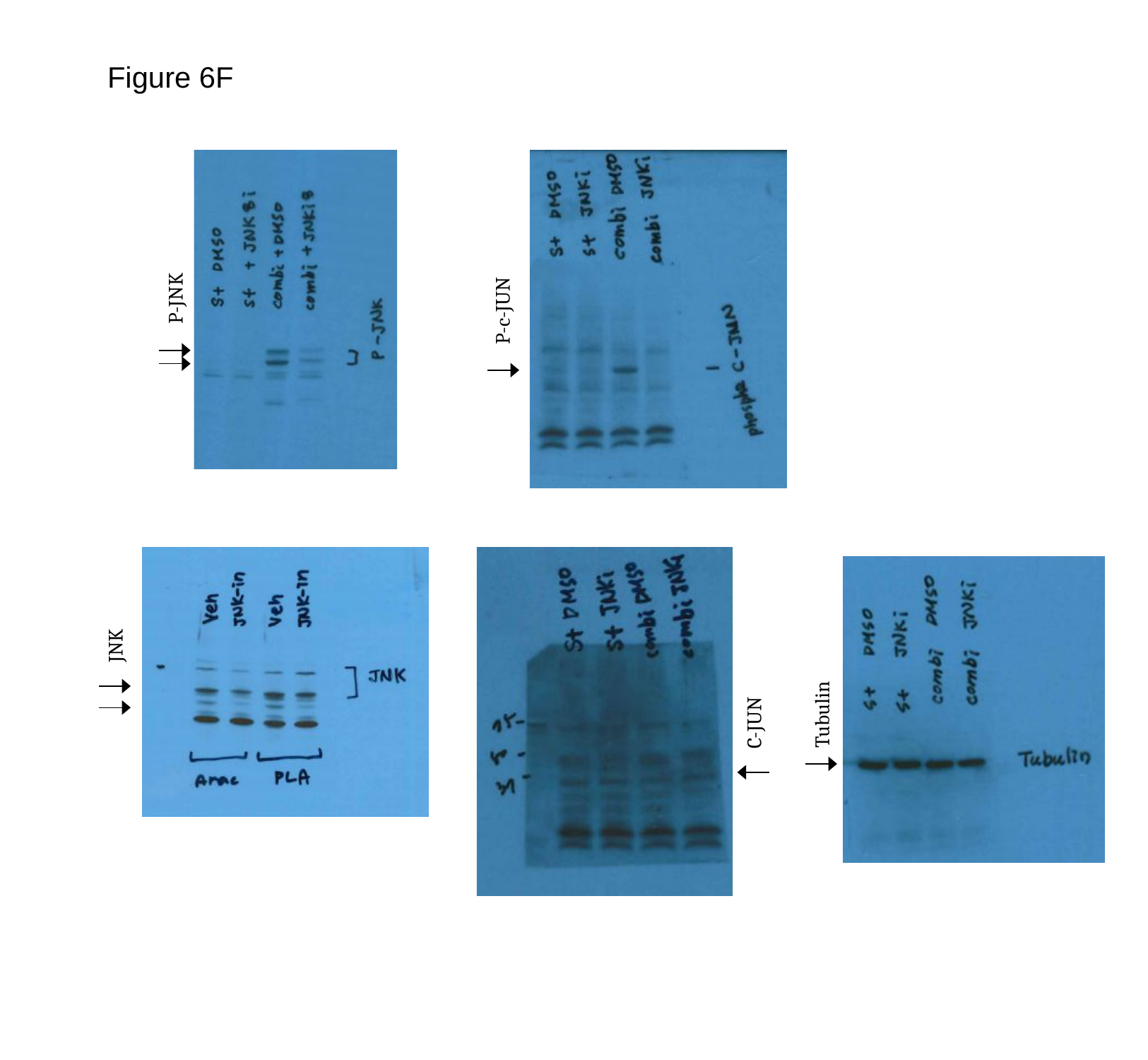

Figure 6F
P-JNK
P-c-JUN
JNK
Tubulin
C-JUN

## Slide 17
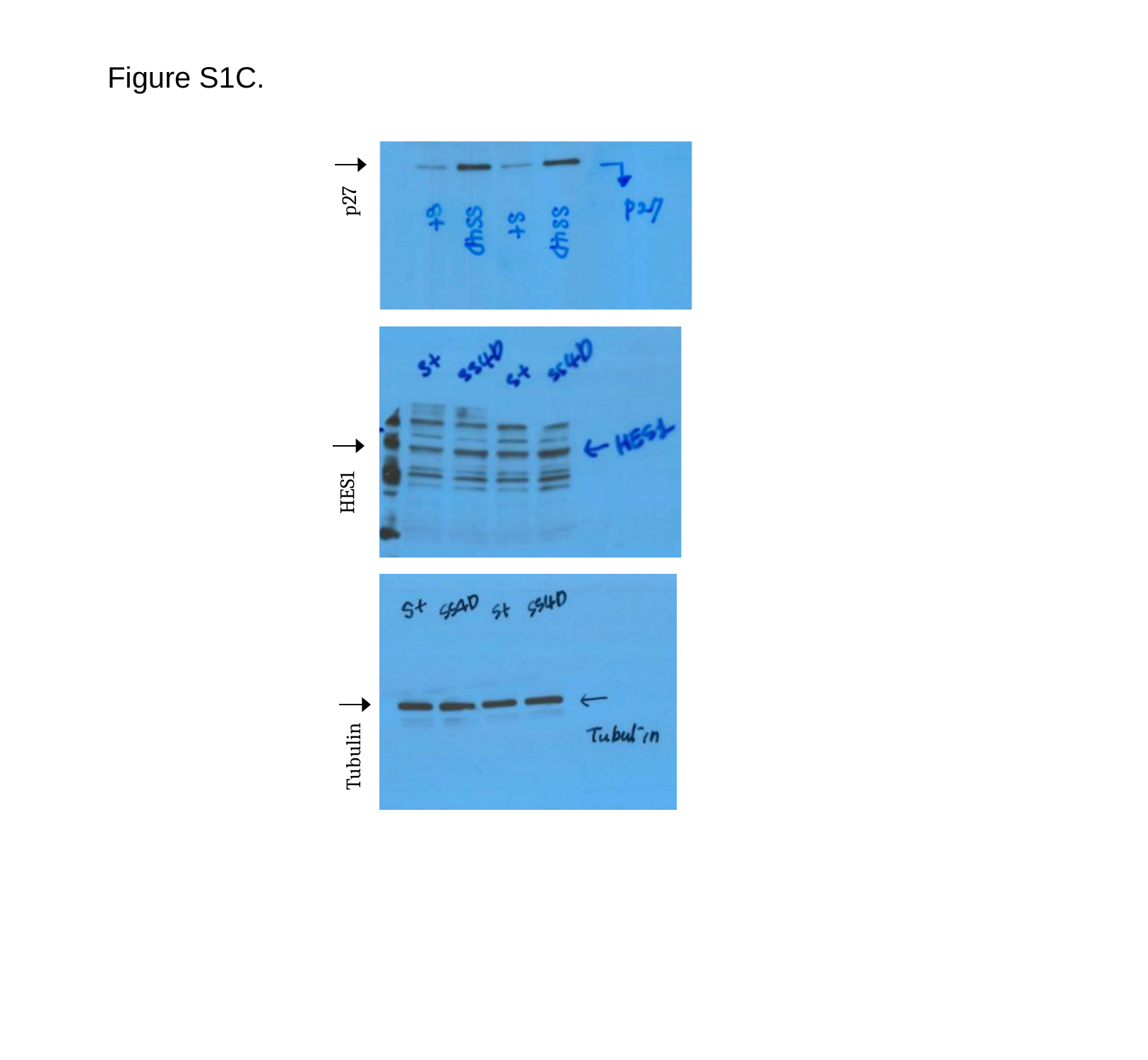

Figure S1C.
p27
HES1
Tubulin

## Slide 18
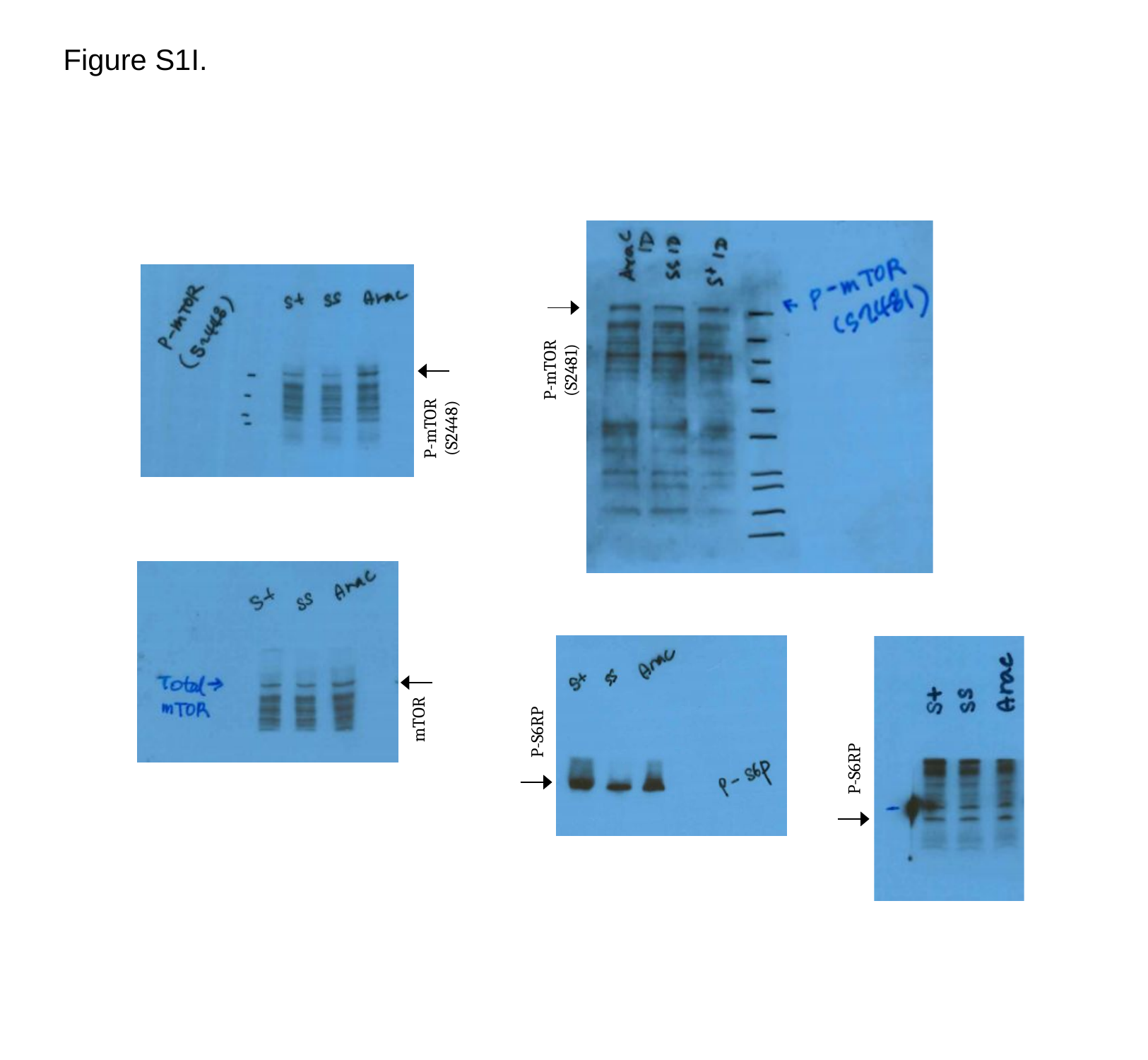

Figure S1I.
P-mTOR (S2481)
P-mTOR (S2448)
mTOR
P-S6RP
P-S6RP

## Slide 19
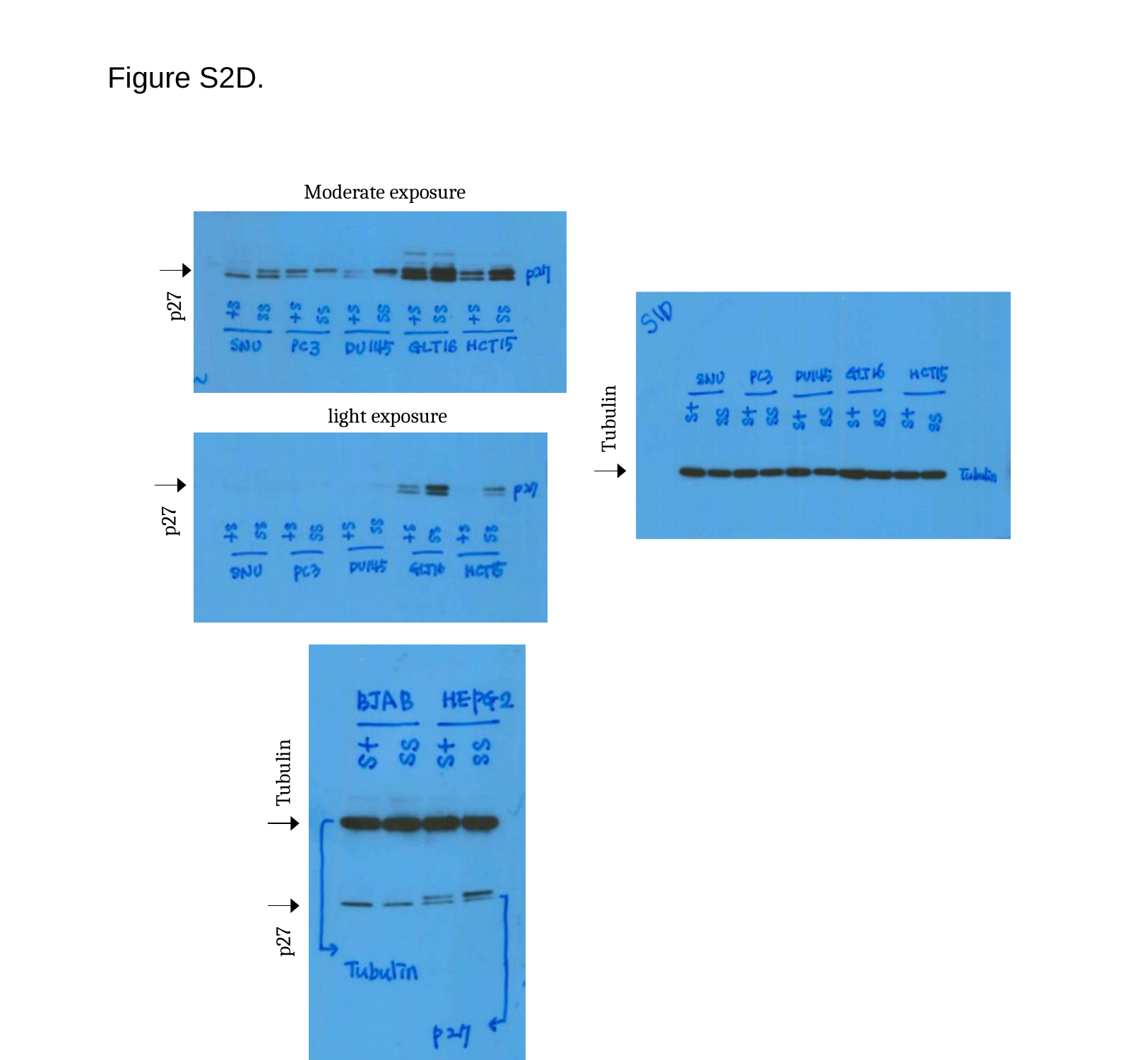

Figure S2D.
Moderate exposure
p27
light exposure
Tubulin
p27
Tubulin
p27

## Slide 20
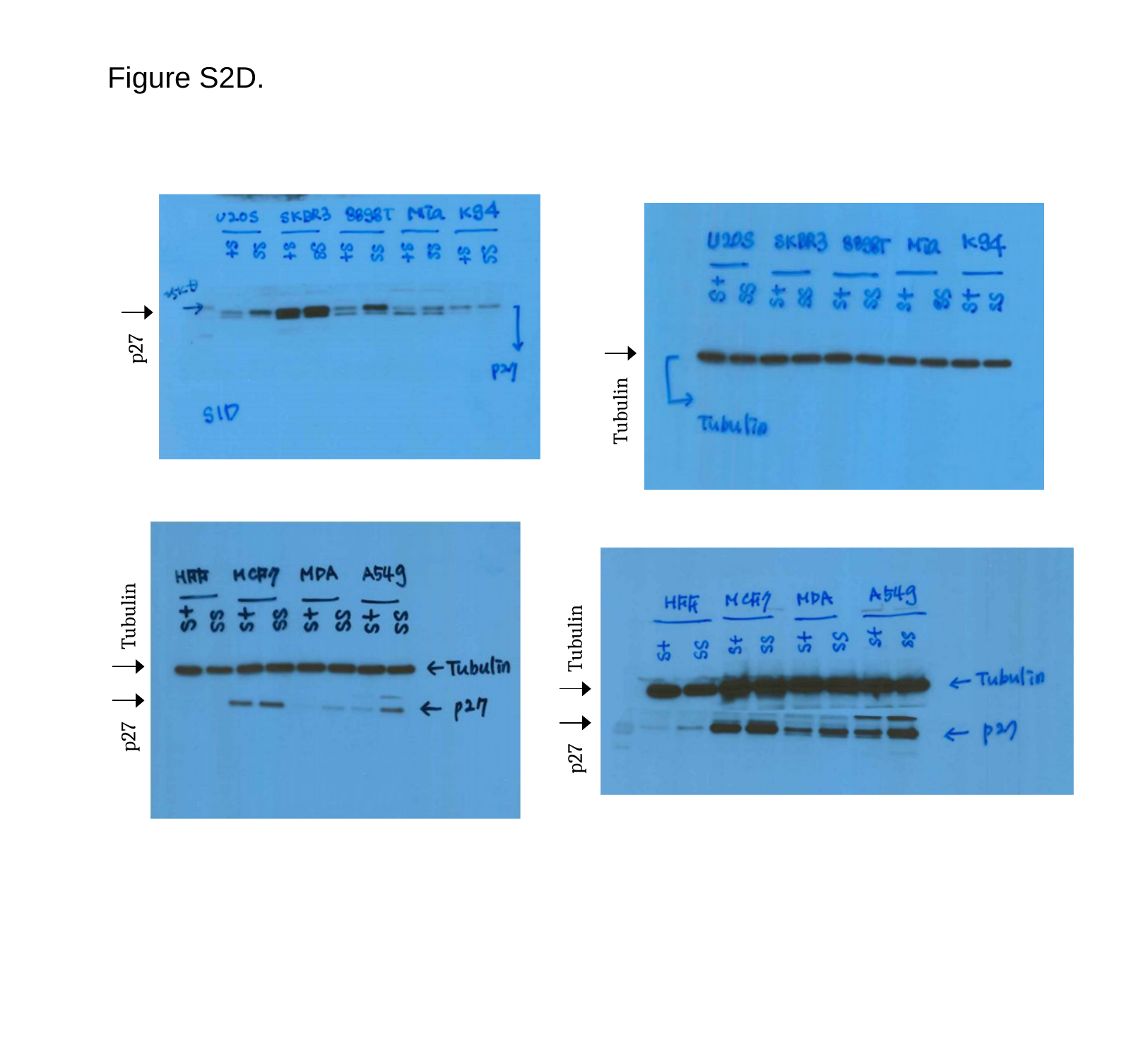

Figure S2D.
p27
Tubulin
Tubulin
Tubulin
p27
p27

## Slide 21
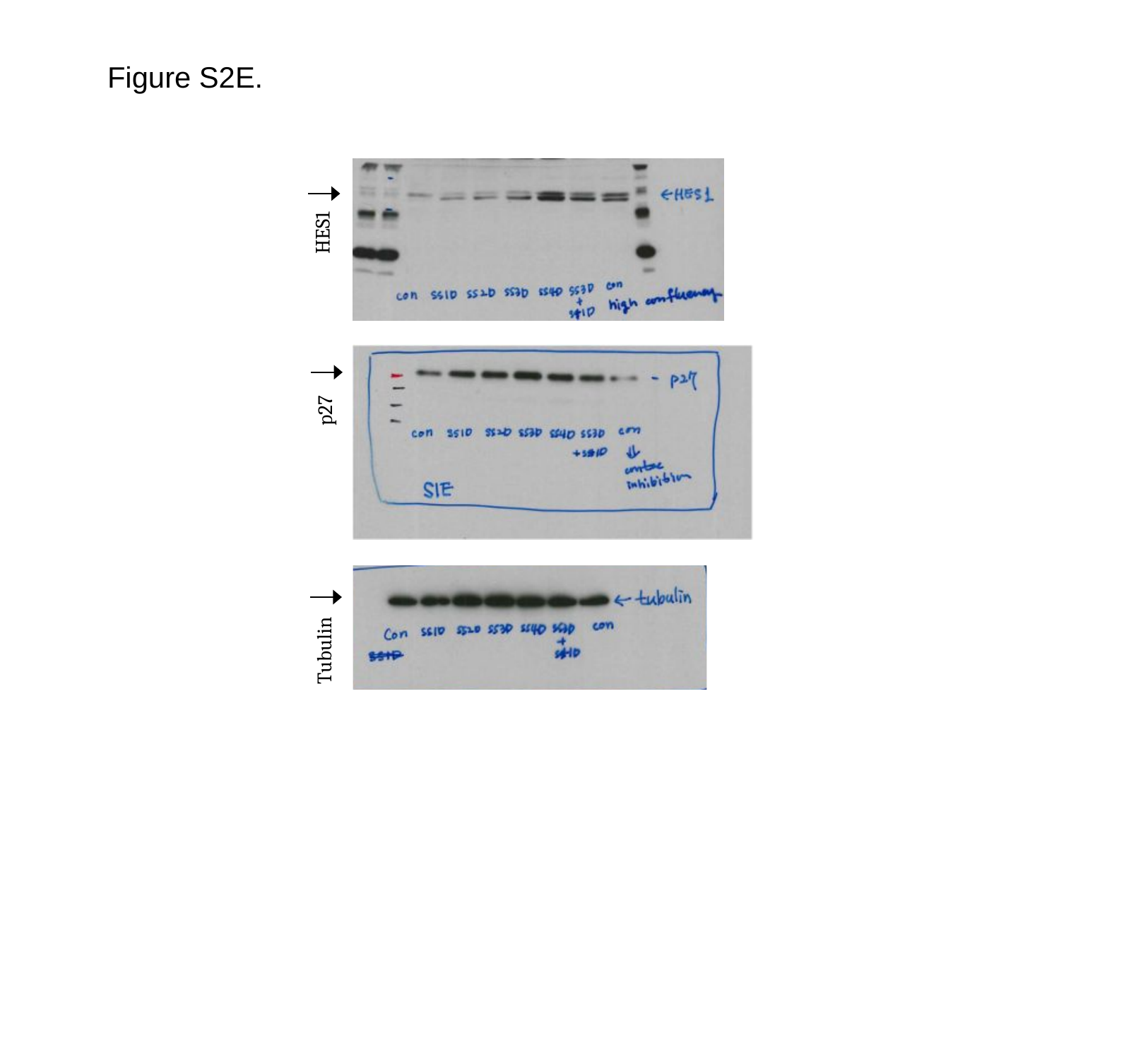

Figure S2E.
HES1
p27
Tubulin

## Slide 22
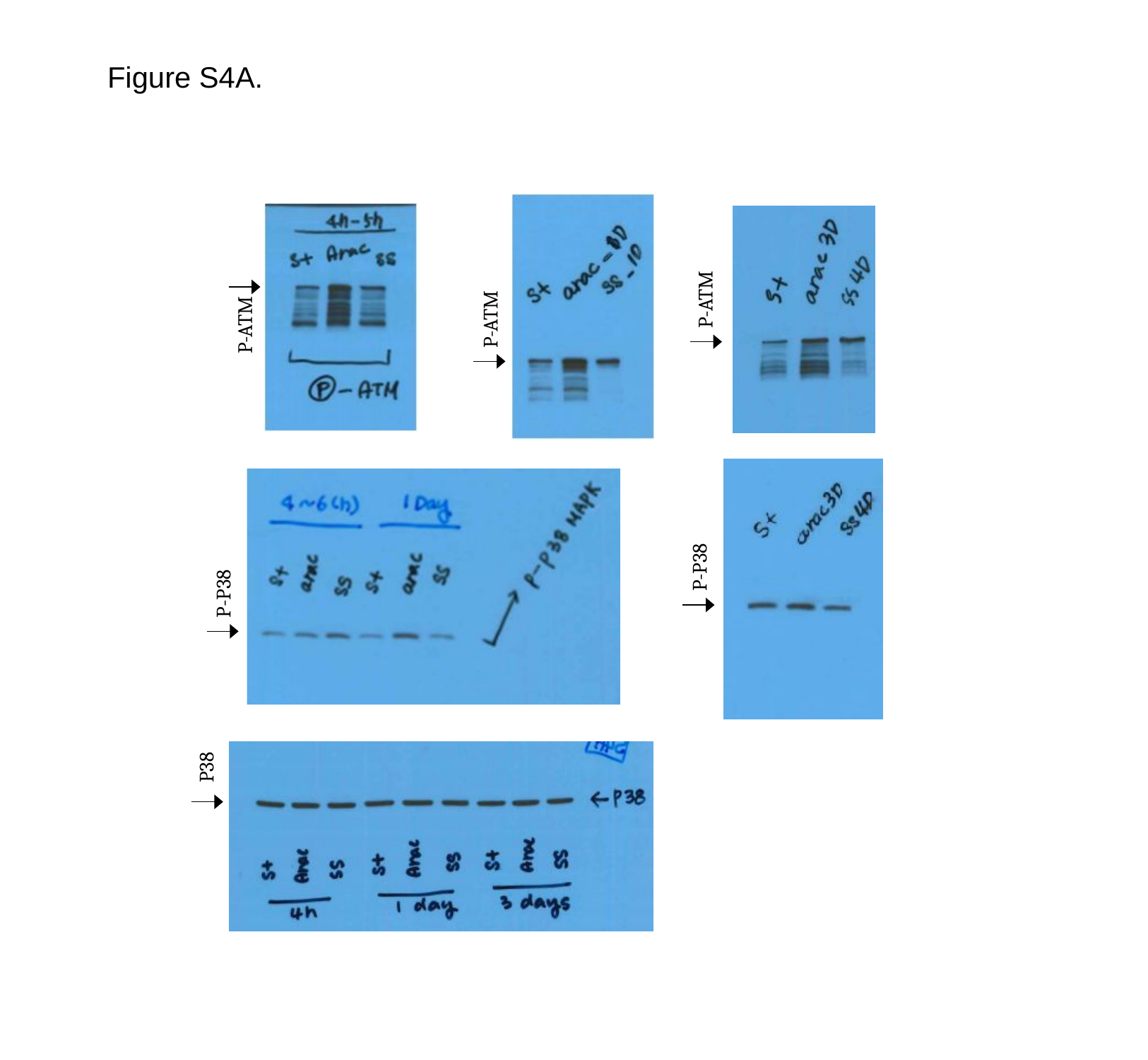

Figure S4A.
P-ATM
P-ATM
P-ATM
P-P38
P-P38
P38

## Slide 23
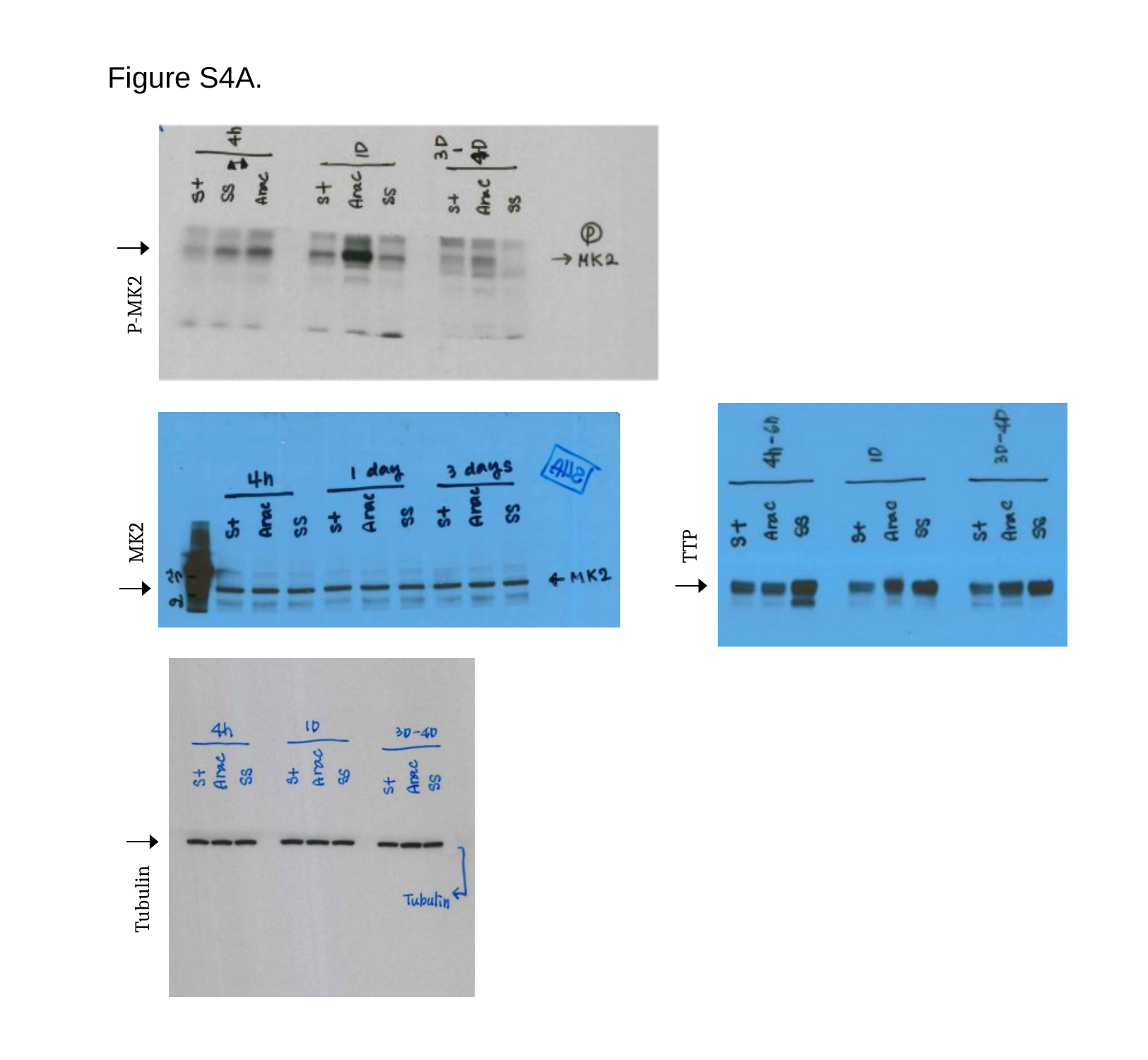

Figure S4A.
P-MK2
MK2
TTP
Tubulin

## Slide 24
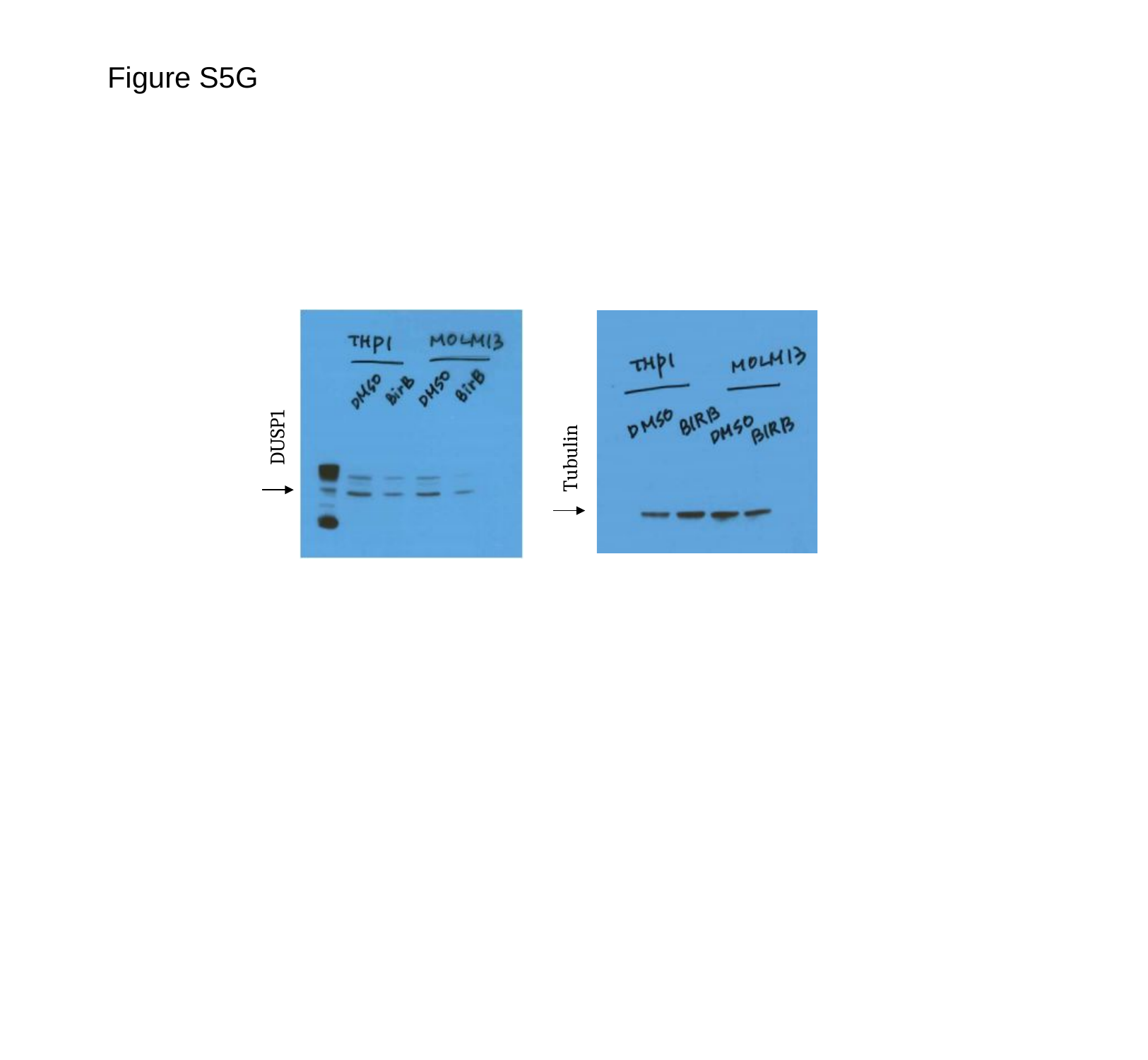

Figure S5G
DUSP1
Tubulin
