# Supplementary material for: A post-transcriptional program of chemoresistance by AU-rich elements and TTP in quiescent leukemic cells
Source: Genome Biol. 2020 Feb 10;21:33. doi: 10.1186/s13059-020-1936-4 (PMC7011231; doi:10.1186/s13059-020-1936-4)
Supplement: Supplementary file 5 — Review history. [file 13059_2020_1936_MOESM5_ESM.docx]

Review History

**First round of review**

**Reviewer 1**

**Comments to author:**

The authors have developed a model in which changes in translation rate occur when leukemic cells are arrested by serum withdrawal or upon treatment with a chemotherapy agents.  The model involves activation of an RNA binding protein that stabilizes particular transcripts. They further demonstrate that specific stabilized transcripts are important for the ability of the chemotherapy-treated cells to resist death.  Treatment with small molecules reverses the chemotherapy resistance in cell culture and when leukemic cells are introduced into mice.

Overall this paper is both mechanistic and immediately clinically relevant.  The results are quite solid and convincing and the logic is mostly strong.  There are specific questions below and when these are addressed, the manuscript should be suitable for publication in Genome Biology.


Comments

1. There seems to be no mention of the data in Figure 1B before the rest of Figure 1, and the data are quantified in Figure 2B. The authors might consider moving Figure 1B to Figure 2.

2. Can the authors please quantify the western blots in Figure 2C to help the reader to understand the extent to which these proteins change.

3. If the authors would like to argue that TOR activity changes, then some westerns showing mTORC1 phosphorylation state would be helpful for this assessment.

4. In terms of TOP genes, the serum starved and AraCS profiles look different.  For serum starvation, there seems to be a selective failure to translate these genes, while for AraCS, ther seems to be a reduction in their RNA expression.  Is TOR activity responsible for both of these mechanisms?  The results make it more important to look at TOR phosphorylation state in the different conditions.

5. Figure legend 2H has words stricken out.

6. In Figure 2F, what is going on with the genes that are upregulated at the translatome level, but not the transcriptome levels and don't have an increase in ribosome occupancy of 1.5-fold? Do they have an increase in ribosome occupancy but it is less than 1.5-fold?

7. It would be helpful to have the Immune Response category added to Figure S2E.

8. On page 11, the authors state "indicating a specific role in chemoresistant cancer cells." For observations in cancer cells but not primary fibroblasts.  To draw this conclusion, it would be helpful to have additional normal cells better matched for cell type to the leukemic cells being studied for comparison.

9. The title: A version of this same manuscript is on Bioarchive and the title includes the word "in cancer quiescence." I think it would be helpful for the field to include a qualification such as in quiescent leukemic cells.

10. The authors argue that TTP is high in the quiescent cells, but phosphorylated, and that the phosphorylation of TTP prevents it from performing its functions. They further seem to be arguing that phosphorylated TTP can somehow act as a dominant negative and prevent non-phosphorylated TTP from causing mRNA decay. The "test" seems to be whether overexpressing TTP that is not phosphorylatable reduces the levels of TTP targets, and it does. But I would expect that overexpressing large amounts of TTP would reduce the levels of TTP targets regardless of whether TTP is or is not an important contributor to the stabilization of some transcripts during quiescence. Therefore, I can't agree with the authors' conclusions based on the data presented.  It seems that the best way to test this would be to make the endogenous TTP unphosphorylatable and test whether those transcripts no longer accumulate. This is a challenging experiment. An alternative would be to knockdown or knockout TTP and see what happens to the transcripts.

11. In Fig 4F, the authors found that treatment with p38 inhibitors affect cell survival if the treatment is early, but not after AraC treatment.  This is a little surprising because there should be new TTP molecules being made that need to be phosphorylated?  Why don't these inhibitors affect the maintenance of a pool of phosphorylated state of TTP and ultimately cell viability?

12. In Fig 4J, there are many different types of cells and data are shown for one "control." What type of cell is this control? Are the bars the ratio of treated to untreated? What do the asterisks indicate? (what is statistically different from what)?

13. The data in Figures S4A and S4B seem to undermine the authors' argument to some extent.  For the serum starved cells, the increase in phosphorylated MK2 is quite modest, and gone by 3 days, while phosphorylated TTP levels rise substantially. A reader is left wondering whether there are also additional contributors to the phosphorylation of TTP besides MK2, especially with serum starvation.

14. In Figure S4D, the authors show the fraction of cells positive for BrdU in the different populations.  The argument had been that AraC treatment results in a state similar to serum withdrawal, but there are a lot of BrdU positive cells here.  How long were the cells treated with BrdU before being analyzed? What would these data look like if the cells were analyzed without AraC treatment? Is AraC making the cells quiescent?

15. Is the legend to Figure S5B correct? Should it be S+/SS or SS/S+?

16. The fold induction of caspase in Ara C-treated cells in response to NF-kB inhibition seems to be lower than the increase in response to TNF-α inhibition (Figure 5H).  Could TNF- α have additional roles?

17. In Figure 6E, why doesn't the large different in phospho-TTP lead to a bigger difference in TNF-α, given the previous figures?

18. In Figure 6F, the authors show the levels of apoptosis with a JNK inhibitor change upon AraC treatment. What if there is no AraC treatment? What if they don't also treat with LY and PFD? Does JNK inhibition affect caspase activity?

19. On page 18, the authors state that the decreased growth of the leukemia cells reflected a loss of "stem cell capacity." Making claims about stemness would require additional assays demonstrating that these cells have self-renewal capacity and the capacity to give rise to other types of cells, none of which are performed.  If these growth assays are to be used, the suggestion is to not make claims about "stem cells."

20. In Figure 8A, what does "control" mean?

21. In Fig 8C-F, is 8th and 22th the days after injection? Are there two groups of mice but each has two photographs of different numbers of mice?

22. Fig 8C, what is plotted in the bar graph?

22. What is the difference between the experiments in Fig 8E and Fig8F?

23. What is "tumor growth" in the bar graphs in Fig 8E and 8F? What are the numbers plotted?

23. Why is Figure 8H with the MLL-AF9 cells shown as a survival curve when Fig8E-H is showing bioluminescence and tumor growth?

**Reviewer 2**

**Comments to author:**

The manuscript showed an interesting post-transcriptional mechanism of chemo-resistance mediated by AU-rich elements and TTP.  The authors reveal a specific proteomic and translation profile that is induced commonly between G0 cells and chemo-resistant leukemic cells. These studies showed how important the post-transcriptional and translational regulation of immune and signaling modulators in chemo-resistance. They further developed a new combination therapy to effectively reduce resistance in cancer cell lines, in vivo and ex vivo models, and AML patient samples, without affecting normal cells. Overall their study has high clinical relevance. However, the manuscript needs further editing to increase the readability and also there are several minor and major questions shall be addressed.
1.      Minor:
a.     Figures should be mentioned in the text following the alphabetic order. However, in the current manuscript, many figures and supplemental figures are cited out of order. This disorder significantly decreased the readability and clarity of the text. To give a few examples:
i.     Fig. 1E -> Fig. 1A-B;
ii.     Fig. 2B -> Fig. 2A;
iii.     Fig. S3F -> Fig. S3C-E;
iv.     Fig. 5B-C -> Fig. 5A;
v.     Fig. 8H -> Fig. 8G.
b.     Figure 7E-F legend is missing.
c.     Figure 2 legend: "in other G0 cells" should be removed.
d.     LPS was not defined at the first occurrence (page 12).
2.     Major:
a.     What is the platform-specific reproducibility between replicates in transcriptome, translatome, and proteome profiles? And what is the data quality and reproducibility of the replicates for each group? Also, the author stated in the supplemental methods that "Two-tailed unpaired t-test or Wilcoxon rank sum test" were used to assess statistical significance. However, if it is microarray data, to better assess differential gene expression data, more specific statistic methods were developed for better performance such as limma package. But it is not clear what was used by the authors.
b.     In Fig 1G, the authors showed that published transcriptome signatures were both upregulated in the SS and AraCS cells compared to SS. Typical GSEA analysis uses transcriptome (mRNA) profiles. It is not clear what the full spectrum of gene set enrichment analysis looks like when using the database such as MSigDB. Showing both different and convergent gene sets enriched in these cells are critical to assess the relevance of these models to study the post-transcriptional regulation in chemo résistance.
c.     Fig. 2D: In the text, the authors stated that both ArcCS and SS cells have lower mTOR activity shown by reduced translation of TOP mRNAs. However, the figure does not support the text, in that only SS show a significant decrease in 2D, while AraCS shows no change. What are the mRNAs measured here? Is it the same between transcriptome and translatome compared in Fig. 2D?
d.     The authors showed that "inflammatory response genes were commonly up-regulated in cancer G0 cells but not normal G0 fibroblasts and do not significantly overlap with senescence-associated secretory pathway (SASP) (Fig. 2I, S2G)". How does the author interpret that in Fig. 2i, the immune response are enriched in THP1, HFF, HEPG2, U2OS, but not MCF7?
e.     What is the % of ARE-bearing up-regulated genes were down-regulated in TTP-AA conditions? How many inflammatory response/chemoresistance genes with ARE in the 3'UTR are stabilized by phosphorylation of TTP? The author only showed TNFα and DUSP1.
f.     What is the exact impact of BIRB on the TNFα protein level? Figure 4C showed the impact of two inhibitor of P38 MAPK, however, the protein list measured by the western blot is different between LY and BIRB. The authors stated that "Treatment with … LY … and … BIRB … reduces TNFα in AraCS cells" when cite Fig. 4C. However, only LY panel showed the results of TNFα, not the BIRB panel. Similarly, how does the BIRB impact on the DUSP1 levels?
g.     The author showed that co-inhibition of p38 MAPK and TNFα sensitizes resistant leukemic cells to AraC treatment in three cell lines: MONOMAC6, MOLM13, and MV4:11. How about THP1?
h.     On page 18, the author stated that "When either p38 MAPK or TNFα was inhibited prior to AraC treatment, moderate apoptosis of chemoresistant cells was observed in primary AML cells (Fig. 8A-B)". However, in AML patients, the inhibition of p38 MAPK by LY + AraC treatment was not shown in Fig. 8A.

**Authors Response**

**Point-by-point responses to the reviewers’ comments:**

Reviewer #1: The authors have developed a model in which changes in translation rate occur when leukemic cells are arrested by serum withdrawal or upon treatment with a chemotherapy agents. The model involves activation of an RNA binding protein that stabilizes particular transcripts. They further demonstrate that specific stabilized transcripts are important for the ability of the chemotherapy-treated cells to resist death. Treatment with small molecules reverses the chemotherapy resistance in cell culture and when leukemic cells are introduced into mice.

Overall this paper is both mechanistic and immediately clinically relevant. The results are quite solid and convincing and the logic is mostly strong. There are specific questions below and when these are addressed, the manuscript should be suitable for publication in Genome Biology.

*Thank you very much for the positive review and very helpful comments. We have now clarified all issues thoroughly with additional data and analyses, in accordance with these comments and suggestions to strengthen the manuscript.*

Comments

1. There seems to be no mention of the data in Figure 1B before the rest of Figure 1, and the data are quantified in Figure 2B. The authors might consider moving Figure 1B to Figure 2.

*We apologize for this & have now rectified it & moved the figure as advised.*

2. Can the authors please quantify the western blots in Figure 2C to help the reader to understand the extent to which these proteins change.

*We have now quantitated Fig. 2C (see figure below), added below the panels, as advised by the reviewer, and discussed them in the main text on pages 7-8 of the results section and page 22 of the discussion section.*

3. If the authors would like to argue that TOR activity changes, then some westerns showing mTORC1 phosphorylation state would be helpful for this assessment.

*We agree with the reviewer that the text misleadingly emphasized mTOR activity, which was incorrectly stated. We have now clarified the text to focus on translation inhibition and not mTOR. As advised by the reviewer, we have now also added data evaluating changes at multiple levels of mTOR pathway: we now show the phosphorylation state on standard markers of mTOR activity, 4EBP and S6, as well as on mTOR, using at least two sites on 4EBP and mTOR.*

*The emphasis was meant to be on reduced canonical translation that is shown by inhibition of the eIF2 pathway, which is commonly and strongly observed in both SS and AraCS cells (Fig. 2C, 4.2 fold increase in phosphorylation of eIF2α in AraCS and 5.3 fold in SS cells, shown above). This blocks one of the two rate limiting steps of canonical translation initiation, and is sufficient to reduce canonical translation. The other rate limiting step of canonical translation initiation is regulated by 4EBP (1); we find that 4EBP is moderately dephosphorylated in these conditions (Fig. 2D), and can partially contribute to reduced translation. Consistent with literature on cell cycle arrested and stress conditions, these steps of translational regulation can be induced by such DNA damage conditions (2) and is transient, such as shown in hypoxia (1, 3).*

*mTOR has many phosphorylation sites that can be transiently regulated by downstream effectors of mTOR, including S6K and others as a feedback mechanism; therefore, mTOR activity is marked by phosphorylation of its downstream targets, which include 4EBP and S6 (1, 3-8). We find that 4EBP shows decreased phosphorylation in both SS and AraCS in Fig. 2D at two critical regulatory sites (50% and 33% decrease in SS and AraCS cells for Thr37/40; 59% and 31% decrease in SS and AraCS cells for Ser65), which can inhibit canonical translation. The dephosphorylation is more pronounced in SS than in AraCS cells, which could account for differences in TOP mRNA regulation; TOP mRNA translation is regulated in SS cells but not in AraCS cells (Fig. S1H). This indicates differences in SS and AraCS cells in mTOR and other signals, which could differentially affect their many downstream effectors, including 4EBP (9- 11) and other effectors (S6, LARP1, TIA1, TIAR) that are implicated in regulating TOP mRNAs and vary in distinct conditions (12-15). This was incorrectly stated and has now been clarified.*

*Regulation of another mTOR target, S6, is also observed (36% and 27% decrease in SS and AraCS cells) in Fig. S1I, although it is more moderate than 4EBP as there are multiple kinases and feedback regulation involved. mTOR itself is affected by feedback from S6K and other kinases downstream of TNFα and other pathways (1, 3-8) that are activated in SS and AraCS cells. Thus, we find that SS or AraCS cells do not significantly reduce mTOR phosphorylation (Fig. S1I), even though the translation inhibitor, 4EBP that is downstream of mTOR signaling, is dephosphorylated at multiple key sites that are normally tested for mTOR regulation (Fig. 2D), and our datasets (Figs. 2I, S2A) show decreased mTOR effectors. Thus, mTOR, unlike the eIF2 pathway, is not fully altered in these conditions, and is not similarly altered for translation regulation in SS and AraCS cells. These differences in mTOR versus 4EBP regulation are observed in cancers and attributed to feedback on mTOR, or other kinases affecting 4EBP in an mTOR independent manner (16-19). These data suggest that we need to be careful to report the regulation of the rate limiting step of translation at the level of 4EBP rather than mTOR regulation. We have revised the text to ensure this is clarified.*

*We have now clarified the text to report that the translation effector, 4EBP, is regulated in SS and AraCS cells, although more moderately in AraCS cells. We have corrected the previous misstatement on TOP mRNAs to carefully report that TOP mRNA translation regulation is observed—as is 4EBP regulation—in SS cells, but not in AraCS cells, where 4EBP regulation is more moderate than in SS cells. Other TOP mRNA effectors (12-15) may also be differentially regulated in SS versus AraCS cells. We also report that coordinate regulation of mTOR with the translation effector, 4EBP, is not significantly observed in SS (15% reduction at one site) and AraCS cells, indicating that the mTOR pathway shows differences at different levels in SS and AraCS cells. Thus, while the eIF2 pathway is strongly inhibited in SS and AraCS cells at similar levels (Fig. 2C), reducing canonical translation—and 4EBP is also moderately regulated with more dephosphorylation in SS than in AraCS cells, which could partially affect translation (Fig. 2D)—other levels of the mTOR pathway are not coordinately modulated or similarly altered in SS and AraCS cells (Fig. 2C-D, S1H-I, main text: pages 7-8 of results, page 22-23 discussion).*

4. In terms of TOP genes, the serum starved and AraCS profiles look different. For serum starvation, there seems to be a selective failure to translate these genes, while for AraCS, ther seems to be a reduction in their RNA expression. Is TOR activity responsible for both of these mechanisms? The results make it more important to look at TOR phosphorylation state in the different conditions.

*We agree with the reviewer that the text misleadingly emphasized TOR activity and incorrectly stated about TOP mRNA regulation. We have now clarified this in the text and added data as discussed above. The emphasis is on reduced canonical translation due to inhibition of the eIF2 pathway (Fig. 2C) that is observed commonly and strongly in SS and AraCS cells. Regulation of eIF2 and of 4EBP and their control over translation were also previously observed in SS cells (20, 21), and either of these two rate limiting steps is sufficient to inhibit canonical translation (1). We observe moderate dephosphorylation of 4EBP that can partially reduce canonical translation (Fig. 2D); this effect is more pronounced in SS cells compared to AraCS cells (50- 59% versus 31-33% at two critical sites). As suggested by the reviewer, although a downstream mTOR target, 4EBP, is regulated in SS and AraCS cells (Figs. 2D), the effect is reduced in AraCS cells. This could account for the differences in TOP mRNA regulation (Fig. S1H), where it is translationally suppressed in SS cells but not in AraCS cells. This indicates differences in SS and AraCS cells in mTOR and other signals, which could differentially affect their many downstream effectors including 4EBP (9-11) and other effectors (S6, LARP1, TIA1, TIAR) that are implicated in regulating TOP mRNAs and vary in distinct conditions (12-15). This was incorrectly stated and has now been clarified. We now focus the text on translation regulation by eIF2 and 4EBP, which is reflected in our past studies (20, 21), and not on mTOR. We have corrected the previous incorrect statement on TOP mRNAs to carefully report that TOP mRNA translation regulation is observed—as is 4EBP regulation—in SS but not in AraCS cells where 4EBP regulation is more moderate than in SS cells. Other TOP mRNA effectors (12-15) may also be differentially regulated in SS versus AraCS cells.*

*As suggested by the reviewer, we have also added data on mTOR pathway phosphorylation of S6 as well as at two sites on mTOR. Regulation of S6, is observed more moderately than 4EBP as there are multiple kinases and feedback regulation involved (Fig. S1I). mTOR itself is affected by feedback from S6K and other kinases downstream of TNFα and other pathways (1, 3-8) that are activated in SS and AraCS cells; thus, we find that SS or AraCS cells do not significantly reduce mTOR phosphorylation (Fig. S1I), even though the downstream target, 4EBP, is clearly dephosphorylated at multiple key sites that are normally tested for mTOR regulation (Fig. 2D), and our datasets (Figs. 2I, S2A) show decreased mTOR effectors. These differences in mTOR versus 4EBP regulation are observed in cancers and attributed to feedback on mTOR, or other kinases affecting 4EBP in an mTOR independent manner (16-19); these data suggest that we need to be careful to report the regulation of the rate limiting step of translation at the level of 4EBP rather than mTOR regulation. We have revised the text to ensure this is clarified. Thus, while the eIF2 pathway is strongly inhibited in SS and AraCS cells at similar levels (Fig. 2C), reducing canonical translation—and 4EBP is also moderately regulated with more dephosphorylation in SS than in AraCS cells, which could partially affect translation (Fig. 2D)— other levels of the mTOR pathway are not coordinately modulated or similarly altered in SS and AraCS cells (Fig. 2C-D, S1H-I, main text:* *pages 7-8 of the results, page 22-23 discussion).*

5. Figure legend 2H has words stricken out.

*We apologize for this typo, this has now been rectified.*

6. In Figure 2F, what is going on with the genes that are upregulated at the translatome level, but not the transcriptome levels and don't have an increase in ribosome occupancy of 1.5-fold? Do they have an increase in ribosome occupancy but it is less than 1.5-fold?

*As the reviewer advised, some of these genes are translationally increased but below our cutoff of 1.5 fold. These are now fully included with all values in Table S1. For example, some genes are increased at the RNA and translatome levels by 1.4-fold and 1.7 fold respectively; so, these have a slight increase in RO. We agree that RO in the venn diagram in Fig. 2F is not fully informative. So, we now show all changes of RO for each gene in Table S1 and have deleted the unclear RO in the venn diagram in Fig. 2F.*

7. It would be helpful to have the Immune Response category added to Figure S2E

*As advised, we have now added this as well as additional categories to Fig. S2A (former Fig. S2E).*

8. On page 11, the authors state "indicating a specific role in chemoresistant cancer cells." For observations in cancer cells but not primary fibroblasts. To draw this conclusion, it would be helpful to have additional normal cells better matched for cell type to the leukemic cells being studied for comparison

*We apologize for the misstatement and have deleted it. We fully agree with the reviewer. We have added data from normal primary human CD34+ alongside cell lines or patient samples that are tested with inhibitors (Fig. 4J, S5I). We agree with the reviewer on global profiling of primary human CD34+ cells. While it was feasible to measure drug effects in normal CD34+ cells as a control, in parallel with our cell lines and patient samples (Fig. 4J, S5I), polysome profiling was technically challenging, needing unfeasible amounts of unfrozen, fresh cells from patient samples that are hard to obtain. As advised, we have deleted this overstatement.*

9. The title: A version of this same manuscript is on Bioarchive and the title includes the word "in cancer quiescence." I think it would be helpful for the field to include a qualification such as in quiescent leukemic cells.

*As advised by the reviewer, we have changed the title to include this qualification.*

10. The authors argue that TTP is high in the quiescent cells, but phosphorylated, and that the phosphorylation of TTP prevents it from performing its functions. They further seem to be arguing that phosphorylated TTP can somehow act as a dominant negative and prevent nonphosphorylated TTP from causing mRNA decay. The "test" seems to be whether overexpressing TTP that is not phosphorylatable reduces the levels of TTP targets, and it does. But I would expect that overexpressing large amounts of TTP would reduce the levels of TTP targets regardless of whether TTP is or is not an important contributor to the stabilization of some transcripts during quiescence. Therefore, I can't agree with the authors' conclusions based on the data presented. It seems that the best way to test this would be to make the endogenous TTP unphosphorylatable and test whether those transcripts no longer accumulate. This is a challenging experiment. An alternative would be to knockdown or knockout TTP and see what happens to the transcripts.

*As advised by the reviewer, these suggested experiments were done and now added (See above figures). As suggested by the reviewer, we used cells where TTP is knocked out, and TTP-AA or TTP-WT (wildtype) are put back (on pages 11-13 and pages 17 in the results section of main text, Figs 3G-H, 4C, 6B-E, S3G-H).*

*Our model figures and wording misleadingly suggested that TTP is playing an active role in stabilization as a phosphorylated form. We have corrected the figure and text to clarify that phosphorylation of TTP prevents its ability to cause decay. The revised models in Figs. 4A, 5A, 6A and S6 now show that TTP causes active ARE mRNA decay but TTP phosphorylation blocks TTP function, allowing ARE mRNAs to be stabilized; this is not due to an active TTP role but due to its inactivation. The previous model figures misleadingly suggested that phospho-TTP prevents non-phosphorylated TTP from causing mRNA decay; this is now corrected: the endogenous TTP is phosphorylated and prevented from causing decay while the overexpressed unphosphorylatable TTP cannot be inactivated and causes decay.*

*To verify this, we did the experiments advised by the reviewer, as shown in Fig. 3G and 6B. (shown above). TTP-deficient cells were transduced with plasmids that express GFP vector, TTP wild-type or the unphosphorylatable form, TTP-AA mutant (22). In Fig. 3G and 6B, expression of TTP-AA mutant more significantly reduced the half-life of TNFα mRNA and total mRNA level of DUSP1 than with expression of TTP-WT in AraC-treated TTP-deficient cells. Furthermore, pharmacological inhibition of TTP phosphorylation with LY2228820 reduced TNFα and DUSP1 levels in Fig. 4C and 6E. Therefore, these data suggest that phosphorylation of TTP contributes to increased level of TTP targets in AraC-treated cells by blocking the decay function of TTP.*

*In addition, we determined genes that are globally affected by TTP phosphorylation, by profiling cells that express TTP-AA and TTP-WT in AraC-treated cells that lack TTP, using microarray (Fig. S3H). Expression of TTP-AA mutant down-regulated 58 genes at the mRNA level compared to TTP-deficient cells. TTP-CLIP (22) data suggest that 40% of mRNAs affected by TTP-AA (23 out of 58, p < 3.585e-05) are associated with TTP protein. The other genes may be due to differences in conditions or are indirectly affected. Furthermore, these mRNAs are stabilized by phosphorylation of TTP; expression of TTP-WT that allows TTP phosphorylation on AraC treatment, up-regulates 53% of genes that are down-regulated by TTP-AA (31 out of 58, p < 0.05, fold change > 1.5). (On pages 11-13 and pages 17 in the results section of main text, Figs 3G-H, 4C, 6B-E, S3G-H).*

11. In Fig 4F, the authors found that treatment with p38 inhibitors affect cell survival if the treatment is early, but not after AraC treatment. This is a little surprising because there should be new TTP molecules being made that need to be phosphorylated? Why don't these inhibitors affect the maintenance of a pool of phosphorylated state of TTP and ultimately cell viability?

*We agree with the reviewer that p38 MAPK inhibitors after AraC treatment should be able to block phosphorylation of new TTP molecules. Within one day of AraC treatment, p38 MAPKMK2 pathway was rapidly activated to phosphorylate TTP (Fig. 4B, S4A-B), stabilizing TNFα and DUSP1 levels, and importantly, promoting downstream survival pathways such as NFkB (Fig. 5B), and turning on survival effectors (Fig. S5F). As expected, early inhibition (prior to AraC) of p38 MAPK for just one day activated pro-apoptotic JNK pathway and reduced TNFα and DUSP1 levels (Fig. 6E). In contrast, as shown in Fig 4F and 4G, treatment with p38 MAPK inhibitor one day after AraC treatment killed only a small population of 10-15% of chemoresistant cells, as the downstream survival pathways that are expressed early, are already turned on (Fig. S5F) and cannot be blocked by the upstream inhibitor targeting p38 MAPK.*

*Thus, while later treatment can inhibit phosphorylation of TTP, in order to overcome the early survival pathway induction and thus block AraC resistance effectively, p38 MAPK needs to be targeted at early time points. Therefore, at later times, targeting TTP and TNFα is not effective in affecting cell viability, as the downstream survival effectors are already turned on and cannot be blocked by these inhibitors of upstream regulator, TNFα (discussed on pages 13-17 in Results, and page 21 in Discussion).*

12. In Fig 4J, there are many different types of cells and data are shown for one "control." What type of cell is this control? Are the bars the ratio of treated to untreated? What do the asterisks indicate? (what is statistically different from what)?

*We have revised our figure legends to clarify this, as advised by the reviewer. As noted by the reviewer, the bars are the ratios of treated to untreated for each cell line or primary cell. To determine the effect of p38 MAPK inhibitor, cells from five different AML cell lines and primary CD34+ were treated with LY2228820 or DMSO vehicle, followed by treatment with AraC (AraC set of bar graphs) or vehicle (DDW/water, S+ set of bar graphs). After these treatments, cell counts, cell viability, and caspase activity were measured. As the reviewer noted, green bar graphs indicate LY2228820+AraC treated cells normalized to DMSO+AraC treated cells for each cell line. Gray bar graphs indicate LY2228820+DDW treated cells normalized to DMSO+DDW treated cells for each cell line. Statistical tests were done between LY2228820 treated and DMSO treated cells in each cell line, and are marked by the asterisks (*=p<0.05). "Control" means DMSO+AraC treated cells in the upper AraC panel, or DMSO+DDW treated cells in the lower S+ panel, and is set to 1 as the reference for each cell line (represented as a white bar). We have clarified this now in the figure legend (Fig. legend 4J, main text page 32).*

13. The data in Figures S4A and S4B seem to undermine the authors' argument to some extent. For the serum starved cells, the increase in phosphorylated MK2 is quite modest, and gone by 3 days, while phosphorylated TTP levels rise substantially. A reader is left wondering whether there are also additional contributors to the phosphorylation of TTP besides MK2, especially with serum starvation.

*We agree with the reviewer that there may be additional contributors to TTP besides MK2 over continued periods of serum-starvation, and have now addressed this, as suggested. In most cases, MK2 is phosphorylated and activated by p38 MAPK, and in turn phosphorylates TTP. However, several reports show that p38 MAPK also directly phosphorylates TTP in macrophages (23, 24). Furthermore, MEKK1 acts as a TTP kinase, together with TNF receptor-associated factor 2 (TRAF2) (25). As phosphorylation of p38 MAPK is reduced 1 day after serum-starvation, MEKK1 can be involved on prolonged serum-starvation to phosphorylate TTP. In addition, protein phosphatase (PP2A) is known to dephosphorylate TTP; if PP2A activity is reduced in prolonged serum-starvation, TTP can remain phosphorylated. This is now discussed in the main text: discussion, page 24.*

14. In Figure S4D, the authors show the fraction of cells positive for BrdU in the different populations. The argument had been that AraC treatment results in a state similar to serum withdrawal, but there are a lot of BrdU positive cells here. How long were the cells treated with BrdU before being analyzed? What would these data look like if the cells were analyzed without AraC treatment? Is AraC making the cells quiescent?

*As the reviewer suggested, AraC and other chemotherapies can select or enrich for quiescent cells (26).We see this with AraC treatment for 3 days in Fig. 1A. In solid tumors, p38 MAPKMK2 induces resistance by arresting the cell cycle. To assess the effect of p38 MAPK on the cell cycle in AraC-treated cells, the number of BrdU-positive cells was measured just 1 day after treatment with AraC and p38 MAPK inhibitor in Fig. S4D. As noted by the reviewer, there are still a lot of BrdU positive cells after only one day of AraC treatment. However, if these leukemic cells were treated with AraC for 3 days, BrdU-positive cells dramatically reduced from 53% to 10% and G0/G1 cells increased from 24% to 51% (Fig. 1A, S1B). Without AraC treatment, we measured cell viability upon inhibition of only p38 MAPK, using multiple assays in Fig. 4F and 4G. We find that p38 MAPK inhibition over three days does not affect cell growth and viability in AML cell lines as shown in the top panel of Fig. 4F and Fig. 4G. We have now clarified this in the legends (supplemental text: figure legend S4, page 13, S1B page 11; main text: figure legend 1 page 29).*

15. Is the legend to Figure S5B correct? Should it be S+/SS or SS/S+?

*We apologize for this and have corrected it. It should be SS/S+.*

16. The fold induction of caspase in AraC-treated cells in response to NF-kB inhibition seems to be lower than the increase in response to TNF-α inhibition (Figure 5H). Could TNF- α have additional roles?

*In most cases, TNFα induces expression of survival genes via NFkB; this was affirmed in our conditions with an NFKB inhibitor (Fig. 5H). As the reviewer suggested, TNFα inhibition induced caspase 3/7 activity that marks apoptosis, more severely than NFkB inhibition when comparing Fig. 5D and 5H. However, the effects on cell viability (Figs. 5D, 5H) of chemoresistant cells were similar between TNFα and NFkB inhibition. So, TNFα inhibition is as effective as NFkB inhibition in reducing survival of resistant cells. Thus, it is unlikely that TNFα activates others apart from NFkB for resistance, although it cannot be ruled out, as there are other pathways downstream of TNFα that could indirectly impinge on survival and on apoptosis (27, 28).*

*There may be a differential effect between pharmacological and genetic inhibition on caspase activity in Figs 5D and 5H: TNFα was genetically inhibited using shRNA in Fig. 5D and NFkB was pharmacologically inhibited using BAY 11-7082 in Fig. 5H. The differences with PFD inhibition of TNFα in Fig. 5F, versus BAY 11-7082 of NFκB in Fig. 5H, may be due to differential drug effects as BAY 11-7082 can affect other pathways that are independent of NFκB (29), leading to differential cell death. The reduced viability in Figs. 5D, 5F and 5H may not be through only one common caspase 3/7 apoptosis pathway as NFkB inhibition with BAY 11-7082 can mediate NFkB-independent cell death that involves other cell death mechanisms such as necrosis (30)—which is not detected by caspase 3/7 that marks apoptosis only, but its impact is seen in viability assays. We have discussed this in the results on page 16, that drug inhibition of NFκB and depletion of TNFα with shRNA are both effective in reducing viability.*

*But caspase activities vary between shRNA depletion and NFκB inhibition, likely due to differences between using drugs for inhibition compared to shRNA, and due to other pathways affected by these drugs, which lead to induction of different, non-apoptotic, cell death mechanisms. Now discussed in the results section of the main text file, page 16.*

17. In Figure 6E, why doesn't the large different in phospho-TTP lead to a bigger difference in TNF-α, given the previous figures?

*Treatment with 300 μg/ml PFD and 5 μM LY2228820 for three days dramatically reduced TNFα in AraC-treated cells in Fig. 4C and 5E. In the triple combination therapy of Fig. 6E, leukemic cells were treated with reduced doses of drugs (150 μg/ml PFD and 2.5 μM LY2228820; half the doses of the drugs used Fig. 4C and 5E) and only for one day to study the early, transiently activated signaling; hence, TNFα was mildly decreased. It requires more time or increased dosage of LY2228820 than used in Fig. 6E, to see the drastic effect of TTP on TNF-α as seen in Figs. 4C and 5E. But, the combination of LY and PFD still reduced TNFα by at least 2-fold upon one day of drug treatment in Fig. 6E. This is now clarified in the results pages 17-18, fig. legends 4-7, pages 31-34 of the main text for Figs. 4C, 5E, 6E, 7B.*

18. In Figure 6F, the authors show the levels of apoptosis with a JNK inhibitor change upon AraC treatment. What if there is no AraC treatment? What if they don't also treat with LY and PFD? Does JNK inhibition affect caspase activity?

*As the reviewer recommended, we have now measured the effect of JNK inhibition in these individual conditions (See above results). JNK inhibition did not have any effect on leukemic cells treated with vehicle or PFD+LY alone. However, JNK inhibition rescued viability and reduced apoptosis mediated by AraC alone as well as by PLA combination (above graph). AraC treatment alone induced phosphorylation of JNK pathway (above blot). Furthermore, PFD+LY treatments prior to AraC greatly enhanced phosphorylation of JNK pathway as shown in Fig. 6E. As expected, JNK inhibition more significantly prevented apoptosis or caspase 3/7 activity of PLA-treated cells compared to AraC-treated cells (above graph): JNK inhibition increased cell viability by 1.6-fold in AraC alone and by 7.6-fold in PLA and decreased caspase activity by 2.7-fold in AraC alone and by 7.5-fold in PLA (above graph). Together, these results suggest that PLA induced apoptosis of chemoresistant cells via JNK activation (in the main text: on page 16- 17 in results, page 23 in discussion, fig legend 6 on pages 33-34).*

19. On page 18, the authors state that the decreased growth of the leukemia cells reflected a loss of "stem cell capacity." Making claims about stemness would require additional assays demonstrating that these cells have self-renewal capacity and the capacity to give rise to other types of cells, none of which are performed. If these growth assays are to be used, the suggestion is to not make claims about "stem cells."

*We fully agree and apologize for this erroneous overstatement; it should have been about survival and growth, as corrected by the reviewer. As advised by the reviewer, we have clarified this sentence and removed the overstatement.*

20. In Figure 8A, what does "control" mean?

*"Control" refers to AraC+vehicle treated cells and is set to 1 for each patient sample (indicated with a white bar, now in Fig. S5I; PFD is dissolved in water, LY222820 is dissolved in DMSO; so, vehicle is water+DMSO). The current Fig. 8A shows individual patient samples where the white bar control in each bar graph represents AraC+vehicle treated cells. AraC+PFD, AraC+LY2228820 or PLA (PFD+LY222820+AraC) treated cells are normalized to AraC+vehicle treated cells (control white bar set to 1 for each sample) for each patient sample. Each bar graph is the ratio of treated (AraC+PFD, AraC+LY2228820 or PLA treated cells) over control (AraC+vehicle treated cells) for each patient sample. We have clarified this now for figure legend 8A, page 35 in the main text and in the supplemental text, Supp. figure legend S5I, page 14.*

21. In Fig 8C-F, is 8th and 22th the days after injection? Are there two groups of mice but each has two photographs of different numbers of mice?

*We have now clarified this in the revised figure legend on page 35. In Fig 8C-G, these numbers indicate days after engraftment with leukemic cells. Five mice were intravenously injected with leukemic cells with luciferase for each group followed by AraC or PLA treatment. Tumor growth was measured at indicated time points using bioluminescence images. Mice that failed to express luciferase (failed tumor engraftment) were discarded. The maximum number of mice for one IVIS image at once is three and so, two images were needed to be taken for each group.*

22. What is the difference between the experiments in Fig 8E and Fig8F?

*We have now clarified these in the revised figure legend on page 35. The number of drug injection is different between the experiments in Fig 8E and Fig 8F. As now shown on the top of each figure, drugs were injected into mice eight times (8X) over four days in Fig. 8E, and six times (6X) over two weeks in Fig. 8F.*

22. Fig 8C, what is plotted in the bar graph?

23. What is "tumor growth" in the bar graphs in Fig 8E and 8F? What are the numbers plotted?

*We have now clarified these in the revised figure legend on page 35. In Fig. 8C, 8E, 8F and 8G, 12 two groups of mice were treated with AraC or PLA therapy. Bioluminescence images were taken 15 days or 22 days after engraftment. Luciferase activity was quantitated, and the relative luciferase activity was plotted as bar graphs to indicate viable leukemic cells. As suggested by the reviewer, "Tumor growth" in this bar graph is misleading, and is replaced by "luciferase activity" in the revision to simplify the figure.*

23. Why is Figure 8H with the MLL-AF9 cells shown as a survival curve when Fig8E-H is showing bioluminescence and tumor growth?

*We measured survival curves for both MLL-AF9 mice in Fig. 8H, and Hoxa9/Meis1 mice in Fig. S5J whose bioluminescence is shown in Fig. 8E-G. We have used HoxA9/Meis1 cells that are transduced with the luciferase gene (31) and measured bioluminescence for tumor growth after PLA drug treatment in Fig. 8E-G. The survival curve for these mice was also measured and shown in Fig. S5J. To confirm the PLA drug effects in a second mouse AML model, we used MLL-AF9 cells that do not contain a luciferase gene (31) and measured survival of mice in Fig. 8H, as it lacks luciferase for bioluminescence.*

Reviewer #2:

The manuscript showed an interesting post-transcriptional mechanism of chemoresistance mediated by AU-rich elements and TTP. The authors reveal a specific proteomic and translation profile that is induced commonly between G0 cells and chemo-resistant leukemic cells. These studies showed how important the post-transcriptional and translational regulation of immune and signaling modulators in chemo-resistance. They further developed a new combination therapy to effectively reduce resistance in cancer cell lines, in vivo and ex vivo models, and AML patient samples, without affecting normal cells. Overall their study has high clinical relevance. However, the manuscript needs further editing to increase the readability and also there are several minor and major questions shall be addressed.

1. Minor: a. Figures should be mentioned in the text following the alphabetic order. However, in the current manuscript, many figures and supplemental figures are cited out of order. This disorder significantly decreased the readability and clarity of the text. To give a few examples:

i. Fig. 1E -> Fig. 1A-B; ii. Fig. 2B -> Fig. 2A; iii. Fig. S3F -> Fig. S3C-E; iv. Fig. 5B-C -> Fig. 5A; v. Fig. 8H -> Fig. 8G. b. Figure 7E-F legend is missing. c. Figure 2 legend: "in other G0 cells" should be removed. d. LPS was not defined at the first occurrence (page 12).

*We apologize for these issues. As advised by the reviewer, we have now thoroughly edited the text to improve clarity. We have re-arranged the text & figures to avoid the figure order issue. Each one of these has been fully rectified and we have proofed the manuscript thoroughly to avoid errors.*

2. Major:

a. What is the platform-specific reproducibility between replicates in transcriptome, translatome, and proteome profiles? And what is the data quality and reproducibility of the replicates for each group? Also, the author stated in the supplemental methods that "Two-tailed unpaired t-test or Wilcoxon rank sum test" were used to assess statistical significance. However, if it is microarray data, to better assess differential gene expression data, more specific statistic methods were developed for better performance such as limma package. But it is not clear what was used by the authors.

*As advised, we have now added the correlations between replicates (See below figures). We obtained good correlations between replicates in the microarray (RNA and translatome) and mass spectrometry data as shown in Fig. S1E. To assess the data reproducibility, Pearson’s correlation coefficients between biological replicates were calculated. As advised by the reviewer, we have now also used limma (32, 33) to identify differentially expressed genes in Figs 2E-G.*

*Each replicate is highly correlated in the microarray datasets (RNA and translatome), with correlation coefficient between 0.978 and 0.993 in Fig. S1E. Correlation coefficients between replicates in the proteomic data are moderate (0.489 in AraCS and 0.720 in SS) compared to the microarray data. However, normalization of the proteome replicate of AraCS or SS by the corresponding S+ replicate, gives good correlation coefficients: 0.717 in AraCS/S+ and 0.812 in SS/S+ (Fig. S1E, bottom panels).*

*As advised by the reviewer, to better assess differentially expressed genes in the microarray data, we used the limma package (32, 33). In Figs. 2E-F, numbers of differentially expressed genes were re-calculated (p-value ≤ 0.05 and fold-change ≥ 1.5). DAVID gene ontology analysis has been done using these new sets of differentially expressed genes in Fig. 2G (Described in main text fig. legend 2E-G, page 30; Supplemental Methods, pages 9-10, Supp. fig. legend S1E, page 11).*

b. In Fig 1G, the authors showed that published transcriptome signatures were both upregulated in the SS and AraCS cells compared to SS. Typical GSEA analysis uses transcriptome (mRNA) profiles. It is not clear what the full spectrum of gene set enrichment analysis looks like when using the database such as MSigDB. Showing both different and convergent gene sets enriched in these cells are critical to assess the relevance of these models to study the post-transcriptional regulation in chemo résistance.

*In Fig 1F (previously 1G), we used transcriptome signatures from chemoresistant cells to address the clinical relevance of our models. As advised by the reviewer, to systematically identify gene sets enriched in G0 cells, we performed GSEA analysis using the full spectrum of hallmark gene sets, as well as gene sets of GO biological process, KEGG, and reactome pathways from the Molecular Signatures Database (MSigDB) (34, 35), to find biological pathways enriched in the translatome datasets of G0 cells from five different cell types compared to corresponding S+ cells, as shown in Fig. S2A. Furthermore, normalized enrichment scores (NES) of selected gene sets that are implicated with quiescent states and cell survival are also shown as a heatmap in Fig. 2I. These results suggested that genes related to cell proliferation are down-regulated and genes related to inflammatory response (pro-inflammatory cytokines and other inflammation regulators) and immune response (interferon-stimulated genes, non-pro-inflammatory immune modulators including immune receptors, antigen presentation and processing regulators) are upregulated. (Described in supplemental text: methods page 9-10, fig. legend S2, page 11-12; main text: results page 9-10, fig. legend 1F, page 29, fig. legend 2I, page 30).*

c. Fig. 2D: In the text, the authors stated that both AraCS and SS cells have lower mTOR activity shown by reduced translation of TOP mRNAs. However, the figure does not support the text, in that only SS show a significant decrease in 2D, while AraCS shows no change. What are the mRNAs measured here? Is it the same between transcriptome and translatome compared in Fig. 2D?

*The text was incorrect and has now been rectified. The same mRNAs are being measured in Fig. S1H (previously Fig. 2D). We agree with the reviewer that the text misleadingly emphasized TOR activity and incorrectly stated about TOP mRNA regulation, which we have now clarified in the text and added data. We have now clarified the text to focus on translation inhibition and not mTOR. As advised by the reviewer, we have now also added data at multiple levels of mTOR pathway (See above figures): we now show the phosphorylation state on standard markers of mTOR activity, 4EBP and S6, and on mTOR, using antibodies against two different phospho-sites on 4EBP and mTOR.*

*The emphasis was meant to be on reduced canonical translation that is shown by inhibition of the eIF2 pathway, which is commonly and strongly observed in both SS and AraCS cells (Fig. 2C, 4.2 fold increase in phosphorylation of eIF2α in AraCS and 5.3 fold in SS cells, shown above). This blocks one of the two rate limiting steps of canonical translation initiation, and is sufficient to reduce canonical translation. The other rate limiting step of canonical translation initiation is regulated by 4EBP (1); we find that 4EBP is moderately phosphorylated in these conditions (Fig. 2D), and can partially contribute to reduced translation. Consistent with literature on cell cycle arrested and stress conditions, these steps of translational regulation can be induced by such DNA damage conditions (2) and is transient, such as shown in hypoxia (1, 3).*

*mTOR has many phosphorylation sites that can be transiently regulated by downstream effectors of mTOR, including S6K and others as a feedback mechanism; therefore, mTOR activity is marked by phosphorylation of its downstream targets, which include 4EBP and S6 (1, 3-8). We find that 4EBP shows decreased phosphorylation in both SS and AraCS in Fig. 2D at two critical regulatory sites (50% and 33% decrease in SS and AraCS cells for Thr37/40; 59% and 31% decrease in SS and AraCS cells for Ser65), which can inhibit canonical translation. The effect is more pronounced in SS than in AraCS cells, which could account for differences in TOP mRNA regulation; TOP mRNA translation is regulated in SS cells but not in AraCS cells (Fig. S1H). This indicates differences in SS and AraCS cells in mTOR and other signals, which could differentially affect their many downstream effectors including 4EBP (9-11) and other effectors (S6, LARP1, TIA1, TIAR) that are implicated in regulating TOP mRNAs and vary in distinct conditions (12-15). This was incorrectly stated and has now been clarified.*

*Regulation of another mTOR target, S6, is also observed (36% and 27% decrease in SS and AraCS cells) in Fig. S1I, although it is more moderate than 4EBP as there are multiple kinases and feedback regulation involved. mTOR itself is affected by feedback from S6K and other kinases downstream of TNFα and other pathways (1, 3-8) that are activated in SS and AraCS cells. Thus, we find that SS or AraCS cells do not significantly reduce mTOR phosphorylation (Fig. S1I), even though the downstream target, 4EBP, is dephosphorylated at multiple key sites that are normally tested for mTOR regulation (Fig. 2D) and our datasets (Figs. 2I, S2A) show decreased mTOR effectors. Thus, mTOR, unlike the eIF2 pathway, is not fully altered in these conditions, and is not similarly altered for translation regulation in SS and AraCS cells. These differences in mTOR versus 4EBP regulation are observed in cancers and attributed to feedback on mTOR, or other kinases affecting 4EBP in an mTOR independent manner (16-19). These data suggest that we need to be careful to report the regulation of the rate limiting step of translation at the level of 4EBP rather than mTOR regulation. We have revised the text to ensure this is clarified.*

*We have now clarified the text to report that the translation effector, 4EBP, is regulated in SS and AraCS cells, although more moderately in AraCS cells. We have corrected the previous misstatement on TOP mRNAs to carefully report that TOP mRNA translation regulation is observed—as is 4EBP regulation—in SS cells, but not in AraCS cells, where 4EBP regulation is more moderate than in SS cells. Other TOP mRNA effectors (12-15) may also be differentially regulated in SS versus AraCS cells. We also report that coordinate regulation of mTOR with the translation effector, 4EBP, is not significantly observed in SS (15% reduction at one site) and AraCS cells, indicating that the mTOR pathway shows differences at different levels in SS and AraCS cells. Thus, while the eIF2 pathway is strongly inhibited in SS and AraCS cells at similar levels (Fig. 2C), reducing canonical translation—and 4EBP is also moderately regulated with 18 more dephosphorylation in SS than in AraCS cells, which could partially affect translation (Fig. 2D)—other levels of the mTOR pathway are not coordinately modulated or similarly altered in SS and AraCS cells (Fig. 2C-D, S1H-I, main text: pages 7-8 of results, pages 22-23 discussion).*

d. The authors showed that "inflammatory response genes were commonly up-regulated in cancer G0 cells but not normal G0 fibroblasts and do not significantly overlap with senescenceassociated secretory pathway (SASP) (Fig. 2I, S2G)". How does the author interpret that in Fig. 2i, the immune response are enriched in THP1, HFF, HEPG2, U2OS, but not MCF7?

*We apologize for the confusion regarding immune response and inflammatory response genes. The heatmaps in Fig. S2A (previously S2G) and Fig. 2I show two separate categories of genes: inflammatory response genes are distinct from the category marked immune genes. These immune response genes include interferon-stimulated genes, which have roles in host defense against viral or bacterial infections, as well as immune receptors, antigen presentation and processing genes and other immune modulators that are not pro-inflammatory cytokines or inflammation regulators. However, the category marked inflammatory response genes lack these interferon-stimulated genes and instead, include pro-inflammatory cytokines and other inflammation regulators.*

*Inflammatory response genes (pro-inflammatory cytokines and other inflammation regulators) are increased in G0 of all the tested cancer cell lines but not in non-cancerous G0 fibroblasts (HFF). In contrast, immune response (interferon-stimulated genes, immune receptors, antigen presentation and processing genes) are up-regulated in THP1, HFF, HEPG2, U2OS, but not in MCF7; as expected, the categories marked interferon-stimulated genes are co-regulated similarly with this immune response gene category that includes interferon-stimulated genes. We clarified this on page 9-10 in the main text results.*

*The inflammatory response genes that include pro-inflammatory cytokines were compared with known SASP genes (that includes many inflammatory response cytokines like IL6 and IL8) and were found to have some overlapping but also many distinct genes with differences in expression, and lack many of those that are critical senescence markers (36). In addition, other known senescence markers were not observed (36-38), suggesting that the gene expression in G0 is not identical to SASP. We have now clarified this in Fig. 2I, S2A, and in main text, pages 9- 10.*

e. What is the % of ARE-bearing up-regulated genes were down-regulated in TTP-AA conditions? How many inflammatory response/chemoresistance genes with ARE in the 3'UTR are stabilized by phosphorylation of TTP? The author only showed TNFα and DUSP1.

*As advised by the reviewer, we addressed how many genes are targeted by TTP and have now added these data in Fig. S3G-H (See above figures) and discussed them in the main text: results page 11-12; supplement text fig. legend S3G-H on pages 12-13. In addition, we have now also shown that TNFα and DUSP1 mRNAs that are regulated by TTP phosphorylation (Fig. 3G, 6B), associate with TTP (Fig. 3H, 6D), using in vivo crosslinking coupled immunoprecipitation (39, 40).*

*First, to address how many G0 genes can be targeted by TTP, we compared TTP target mRNAs, identified by cross-linking and immunoprecipitation (CLIP), with the up-regulated translatome in G0 leukemic cells (22). Importantly, 34% of the up-regulated translatome in G0 leukemic cells (166 out of 490 RNAs) are associated with TTP (Fig. S3G). Furthermore, the overlap between the two groups of genes is statistically very significant (p < 2.71e-21). Additionally, 49% of the ARE-bearing genes of the translatome up-regulated in G0 (59 out of 121 genes; p<7.302e-16 for statistical significance of overlap) are associated with TTP (shown above and in Fig. S3G).*

*Second, to determine the genes regulated by TTP phosphorylation, we stably expressed TTP-AA and TTP-WT in AraC-treated cells that lack TTP (22), followed by transcriptome profiling using microarray. Expression of TTP-AA mutant down-regulated 58 genes at the mRNA level compared to TTP-deficient cells (shown above and in Fig. S3H). Defense response and inflammatory response genes are significantly enriched in these 58 genes (p-value of 3.8E-6 and p-value of 9.6E-5, respectively). TTP-CLIP data suggest that 40% of mRNAs affected by TTPAA (23 out of 58, p < 3.6E-05) are associated with TTP protein (Fig. S3H). The other genes may be due to differences in conditions or are indirectly affected by TTP. Furthermore, these mRNAs are stabilized by phosphorylation of TTP; expression of TTP-WT that allows TTP phosphorylation, up-regulates 53% of genes that are down-regulated by TTP-AA (31 out of 58, p < 0.05, fold change > 1.5). Of these 58, 18 genes have AREs and are stabilized by phosphorylation of TTP (Fig. S3H). These are shown in Fig. S3G-H (above figures), & discussed them in the main text: results page 11-12; supplement text fig. legend S3G-H on pages 12-13.*

f. What is the exact impact of BIRB on the TNFα protein level? Figure 4C showed the impact of two inhibitor of P38 MAPK, however, the protein list measured by the western blot is different between LY and BIRB. The authors stated that "Treatment with … LY … and … BIRB … reduces TNFα in AraCS cells" when cite Fig. 4C. However, only LY panel showed the results of TNFα, not the BIRB panel. Similarly, how does the BIRB impact on the DUSP1 levels?

*As advised by the reviewer, the effect of BIRB on TNFα has now been added to Fig. 4C (shown left). As with TNFα, a similar effect of BIRB was also seen on DUSP1 in Fig. S5G. These are discussed in the main text: pages12-13, 16-17 in the results, page 22-24 of the discussion, Fig. legend 4, page 31; and in supplemental text, supp fig. legend S5G, page 14.*

g. The author showed that co-inhibition of p38 MAPK and TNFα sensitizes resistant leukemic cells to AraC treatment in three cell lines: MONOMAC6, MOLM13, and MV4:11. How about THP1 ?

*As suggested by the reviewer, we also measured the effect of co-inhibition of p38 MAPK and TNFα on survival of chemoresistant cells in the THP1 cell line (See above figures), which was erroneously left out. Consistent with the other cell lines, as now shown in Fig. 7B, co-inhibition eliminated about 80% of AraC-resistant cells in THP1. We have now rectified the text (page 17- 18 of the results section, fig. legend 7B on page 34, discussed on page 23 of discussion) and figure 7B to ensure that the THP1 data is also clear alongside these other cell lines. This figure is to show that it works in other cell lines as well as in THP1 cells.*

h. On page 18, the author stated that "When either p38 MAPK or TNFα was inhibited prior to AraC treatment, moderate apoptosis of chemoresistant cells was observed in primary AML cells (Fig. 8A-B)". However, in AML patients, the inhibition of p38 MAPK by LY + AraC treatment was not shown in Fig. 8A.

*As advised by the reviewer, primary cells from AML patients were treated with LY or PFD prior to AraC, followed by assessment of cell viability (see above figures). In Fig. 8A, we compared 22 each drug combination in the same primary cells. In Fig. S5I, combination of LY or PFD with AraC was compared with AraC. Treatment with either LY or PFD prior to AraC induced only moderate apoptosis of chemoresistant cells, compared to treatment with PFD+LY prior to AraC in four different primary cells as shown in Fig. 8A and in Fig. S5I. Normal CD34+ cells are not affected by the drugs (indicated with red asterisk in , lower panel above, Fig. S5I). These results are also consistent with our observation in primary cells from two murine AML models in Fig. 8B (discussed on pages 18-19 in the results and page 23 in the discussion, fig. legend 8 on page 35 of the main text; supplemental text, supp fig. legend S5I on page 14).*

References

1. Sonenberg N, Hinnebusch AG. Regulation of translation initiation in eukaryotes: mechanisms and biological targets. Cell. 2009;136(4):731-45.

2. Tee AR, Proud CG. DNA-damaging agents cause inactivation of translational regulators linked to mTOR signalling. Oncogene. 2000;19(26):3021-31.

3. Koritzinsky M, Magagnin MG, van den BT, Seigneuric R, Savelkouls K, Dostie J, et al. Gene expression during acute and prolonged hypoxia is regulated by distinct mechanisms of translational control. EMBO J. 2006;25(5):1114-25.

4. Bodur C, Kazyken D, Huang K, Ekim Ustunel B, Siroky KA, Tooley AS, et al. The IKKrelated kinase TBK1 activates mTORC1 directly in response to growth factors and innate immune agonists. Embo j. 2018;37(1):19-38.

5. Caron E, Ghosh S, Matsuoka Y, Ashton-Beaucage D, Therrien M, Lemieux S, et al. A comprehensive map of the mTOR signaling network. Mol Syst Biol. 2010;6:453.

6. Foster KG, Fingar DC. Mammalian target of rapamycin (mTOR): conducting the cellular signaling symphony. J Biol Chem. 2010;285(19):14071-7.

7. Levin VA, Panchabhai SC, Shen L, Kornblau SM, Qiu Y, Baggerly KA. Different changes in protein and phosphoprotein levels result from serum starvation of high-grade glioma and adenocarcinoma cell lines. Journal of proteome research. 2010;9(1):179-91.

8. Pirkmajer S, Chibalin AV. Serum starvation: caveat emptor. American journal of physiology Cell physiology. 2011;301(2):C272-9.

9. Thoreen CC, Chantranupong L, Keys HR, Wang T, Gray NS, Sabatini DM. A unifying model for mTORC1-mediated regulation of mRNA translation. Nature. 2012;485(7396):109-13.

10. Hsieh AC, Liu Y, Edlind MP, Ingolia NT, Janes MR, Sher A, et al. The translational landscape of mTOR signalling steers cancer initiation and metastasis. Nature. 2012;485(7396):55-61.

11. Alain T, Morita M, Fonseca BD, Yanagiya A, Siddiqui N, Bhat M, et al. eIF4E/4E-BP ratio predicts the efficacy of mTOR targeted therapies. Cancer Res. 2012;72(24):6468-76.

12. Damgaard CK, Lykke-Andersen J. Translational coregulation of 5'TOP mRNAs by TIA1 and TIAR. Genes Dev. 2011;25(19):2057-68.

13. Tcherkezian J, Cargnello M, Romeo Y, Huttlin EL, Lavoie G, Gygi SP, et al. Proteomic analysis of cap-dependent translation identifies LARP1 as a key regulator of 5'TOP mRNA translation. Genes & development. 2014;28(4):357-71.

14. Miloslavski R, Cohen E, Avraham A, Iluz Y, Hayouka Z, Kasir J, et al. Oxygen sufficiency controls TOP mRNA translation via the TSC-Rheb-mTOR pathway in a 4E-BPindependent manner. J Mol Cell Biol. 2014;6(3):255-6

15. Fonseca BD, Zakaria C, Jia J-J, Graber TE, Svitkin Y, Tahmasebi S, et al. La-related Protein 1 (LARP1) Represses Terminal Oligopyrimidine (TOP) mRNA Translation Downstream of mTOR Complex 1 (mTORC1). The Journal of biological chemistry. 2015;290(26):15996- 6020.

16. Hay N, Sonenberg N. Upstream and downstream of mTOR. Genes Dev. 2004;18(16):1926-45.

17. Villalonga P, Fernandez de Mattos S, Ridley AJ. RhoE inhibits 4E-BP1 phosphorylation and eIF4E function impairing cap-dependent translation. J Biol Chem. 2009;284(51):35287-96.

18. Nawroth R, Stellwagen F, Schulz WA, Stoehr R, Hartmann A, Krause BJ, et al. S6K1 and 4E-BP1 Are Independent Regulated and Control Cellular Growth in Bladder Cancer. PLOS ONE. 2011;6(11):e27509.

19. Batool A, Aashaq S, Andrabi KI. Reappraisal to the study of 4E-BP1 as an mTOR substrate - A normative critique. Eur J Cell Biol. 2017;96(4):325-36.

20. Lee S, Truesdell SS, Bukhari SI, Lee JH, Letonqueze O, Vasudevan S. Upregulation of eIF5B controls cell-cycle arrest and specific developmental stages. Proc Natl Acad Sci U S A. 2014;111(41):E4315-E22.

21. Bukhari SI, Truesdell SS, Lee S, Kollu S, Classon A, Boukhali M, et al. A Specialized Mechanism of Translation Mediated by FXR1a-Associated MicroRNP in Cellular Quiescence. Mol Cell. 2016;61(5):760-73.

22. Tiedje C, Diaz-Munoz MD, Trulley P, Ahlfors H, Laaß K, Blackshear PJ, et al. The RNA-binding protein TTP is a global post-transcriptional regulator of feedback control in inflammation. Nucleic Acids Research. 2016;44(15):7418-40.

23. Carballo E, Cao H, Lai WS, Kennington EA, Campbell D, Blackshear PJ. Decreased sensitivity of tristetraprolin-deficient cells to p38 inhibitors suggests the involvement of tristetraprolin in the p38 signaling pathway. J Biol Chem. 2001;276(45):42580-7.

24. Chrestensen CA, Schroeder MJ, Shabanowitz J, Hunt DF, Pelo JW, Worthington MT, et al. MAPKAP kinase 2 phosphorylates tristetraprolin on in vivo sites including Ser178, a site required for 14-3-3 binding. J Biol Chem. 2004;279(11):10176-84.

25. Schichl YM, Resch U, Lemberger CE, Stichlberger D, de Martin R. Novel phosphorylation-dependent ubiquitination of tristetraprolin by mitogen-activated protein kinase/extracellular signal-regulated kinase kinase kinase 1 (MEKK1) and tumor necrosis factor receptor-associated factor 2 (TRAF2). J Biol Chem. 2011;286(44):38466-77.

26. Tyson DR, Garbett SP, Frick PL, Quaranta V. Fractional proliferation: a method to deconvolve cell population dynamics from single-cell data. Nat Methods. 2012;9(9):923-8.

27. Hsu H, Shu HB, Pan MG, Goeddel DV. TRADD-TRAF2 and TRADD-FADD interactions define two distinct TNF receptor 1 signal transduction pathways. Cell. 1996;84(2):299-308.

28. Annibaldi A, Meier P. Checkpoints in TNF-Induced Cell Death: Implications in Inflammation and Cancer. Trends Mol Med. 2018;24(1):49-65.

29. Strickson S, Campbell D, Emmerich C, A K, L P, Ritorto MS, et al. The antiinflammatory drug BAY 11-7082 suppresses the MyD88-dependent signaling network by targeting the ubiquitin system. Biochemical Journal. 2013.

30. Rauert-Wunderlich H, Siegmund D, Maier E, Giner T, Bargou RC, Wajant H, et al. The IKK inhibitor Bay 11-7082 induces cell death independent from inhibition of activation of NFkappaB transcription factors. PLoS One. 2013;8(3):e59292

31. Sykes DB, Kfoury YS, Mercier FE, Wawer MJ, Law JM, Haynes MK, et al. Inhibition of Dihydroorotate Dehydrogenase Overcomes Differentiation Blockade in Acute Myeloid Leukemia. Cell. 2016;167(1):171-86.

32. Ritchie ME, Phipson B, Wu D, Hu Y, Law CW, Shi W, et al. limma powers differential expression analyses for RNA-sequencing and microarray studies. Nucleic Acids Res. 2015;43(7):e47.

33. Gautier L, Cope L, Bolstad BM, Irizarry RA. affy--analysis of Affymetrix GeneChip data at the probe level. Bioinformatics (Oxford, England). 2004;20(3):307-15.

34. Mootha VK, Lindgren CM, Eriksson K-F, Subramanian A, Sihag S, Lehar J, et al. PGC1α-responsive genes involved in oxidative phosphorylation are coordinately downregulated in human diabetes. Nature Genetics. 2003;34(3):267-73.

35. Subramanian A, Tamayo P, Mootha VK, Mukherjee S, Ebert BL, Gillette MA, et al. Gene set enrichment analysis: A knowledge-based approach for interpreting genome-wide expression profiles. Proceedings of the National Academy of Sciences. 2005;102(43):15545.

36. Coppe JP, Desprez PY, Krtolica A, Campisi J. The senescence-associated secretory phenotype: the dark side of tumor suppression. Annu Rev Pathol. 2010;5:99-118. doi: 10.1146/annurev-pathol-121808-102144.:99-118.

37. Serrano M, Lin AW, McCurrach ME, Beach D, Lowe SW. Oncogenic ras provokes premature cell senescence associated with accumulation of p53 and p16INK4a. Cell. 1997;88(5):593-602.

38. Loayza-Puch F, Drost J, Rooijers K, Lopes R, Elkon R, Agami R. p53 induces transcriptional and translational programs to suppress cell proliferation and growth. Genome Biol. 2013;14(4):R32.

39. Chen AJ, Paik JH, Zhang H, Shukla SA, Mortensen R, Hu J, et al. STAR RNA-binding protein Quaking suppresses cancer via stabilization of specific miRNA. Genes Dev. 2012;26(13):1459-72.

40. Ule J, Jensen K, Mele A, Darnell RB. CLIP: a method for identifying protein-RNA interaction sites in living cells. Methods. 2005;37(4):376-86.

**Second round of review**

**Reviewer 1**

The authors did an excellent job responding to my comments. Their careful explanations and changes are very much appreciated. The manuscript looks good to me now. I believe there are some changes in the discussion that weren't highlighted in yellow, but the changes look good and that's not a problem.

**Reviewer 2**

The authors responded to all my comments well. Only comment c and e need some clarfication:

To answer my question, the authors updated Figure 2C. On Page 7, the authors stated: “We find that two eIF2 kinases, PKR and PERK, are activated and significantly increase eIF2α phosphorylation in SS and AraCS G0 leukemic cells (Fig. 2C, 5.3 fold in SS and 4.2 fold in AraCS cells),”. However, it is not clear how the authors derived the “5.3 fold” and “4.2 fold” from the updated Figure 2C. Please explain.

To respond comment e, the authors stated: "Furthermore, the overlap between
the two groups of genes is statistically very significant (p < 2.71e-21). Additionally, 49% of the
ARE-bearing genes of the translatome up-regulated in G0 (59 out of 121 genes; p <7.302e-16 for
statistical significance of overlap) are associated with TTP (shown above and in Fig. S3G)."
Please provide the specific statistical test used for deriving the p-values in the text.

**Authors Response**

**Point-by-point responses to the reviewers’ comments:**

Reviewer #2: The authors responded to all my comments well. Only comment c and e need some clarfication:

*We thank the reviewers for their positive and helpful comments and guidance on our manuscript.*

To answer my question, the authors updated Figure 2C. On Page 7, the authors stated: “We find that two eIF2 kinases, PKR and PERK, are activated and significantly increase eIF2 phosphorylation in SS and AraCS G0 leukemic cells (Fig. 2C, 5.3 fold in SS and 4.2 fold in AraCS cells),”. However, it is not clear how the authors derived the “5.3 fold” and “4.2 fold” from the updated Figure 2C. Please explain.

*Apart from quantitation of levels in SS and AraCS compared to S+ cells (Fig. 2C, below blots), we calculated fold change of phosphorylated eIF2α over total eIF2α, based on the quantitation below the blots, but did not describe it clearly. The ratio of phospho-eIF2α over total eIF2α increased by 5.3 fold (3.9/0.74) in SS and 4.2 fold (3.2/0.75) in AraCS cells. As advised, we now clarified this in the Results section of the main text on page 7, where this figure is described.*

To respond comment e, the authors stated: "Furthermore, the overlap between the two groups of genes is statistically very significant (p < 2.71e-21). Additionally, 49% of the ARE-bearing genes of the translatome up-regulated in G0 (59 out of 121 genes; p <7.302e-16 for statistical significance of overlap) are associated with TTP (shown above and in Fig. S3G)." Please provide the specific statistical test used for deriving the p-values in the text.

*The statistical significance of overlaps between two different groups of genes was assessed using hypergeometric probability test (http://nemates.org/MA/progs/overlap_stats.cgi), with the total number of proteins being 26809, based on our arrays. As advised by the reviewer, we have now clarified this in Methods: statistical tests on page 35, and in Results on page 12 in the main text.*
